# A Mixed-Valent and High-Spin Vanadium Phosphide

**DOI:** 10.1021/jacs.5c20647

**Published:** 2026-02-27

**Authors:** Aswin Chandran, Christian Sandoval-Pauker, Balazs Pinter, Tom Vosch, Fabrice Wilhelm, Andrei Rogalev, Kasper S. Pedersen, Anders Reinholdt

**Affiliations:** † Department of Chemistry, 5193 Lund University, Naturvetarvägen 22, 22100 Lund, Sweden; ‡ Department of Chemical and Biomolecular Engineering, 3990 Rice University, 6100 Main Street, Houston, Texas 770052, United States; § Department of Chemistry and Biochemistry, 12337 University of Texas at El Paso, El Paso, Texas 79968, United States; ∥ Department of Chemistry, University of Copenhagen, Universitetsparken 5, DK-2100 Copenhagen, Denmark; ⊥ 55553 ESRF—The European Synchrotron Radiation Facility, CS 40220, 38043 Grenoble, Cedex 9, France; # Department of Chemistry, Technical University of Denmark, Kemitorvet, DK-2800 Kgs. Lyngby, Denmark

## Abstract

Accessing high-spin configurations of transition metal phosphides defines a dividing line that prevents common properties of solid-state materials from being replicated within multiple-bonded molecular analogs. Here, we report the synthesis of a V^III^ phosphaethynolate complex, [(pyrNdipp)_2_V­(PCO)] (**2**) in a halide metathesis with Na­(OCP). Exposure of **2** to Lewis-basic ligands induces a one-electron reductive elimination of the PCO^–^ moiety, generating V^II^ complexes [(pyrNdipp)_2_V­(L)_2_] (L = THF, DMAP; **3** ^**THF**^, **3** ^**DMAP**^). When **2** is instead photolyzed, a cascade of reduction, decarbonylation, and multiple-bond formation steps affords a high-spin and mixed-valent vanadium phosphide, [(pyrNdipp)_2_V=P=V­(pyrNdipp)_2_] (**4**) comprising formal [V_2_^III, IV^] nodes. Structural characterization coupled with vibrational, UV–visible, and X-ray spectroscopic studies reveals an *S*_4_ symmetrical [V=P=V] centered architecture conforming to a fully delocalized, mixed-valency description. Theoretical studies demonstrate that **4** evades spin-pairing by leveraging the weak ligand-field splitting at the vanadium nodes, leading to a high-spin, *S*_T_ = 3/2 ground state of this multiple-bonded, weakly Jahn–Teller distorted system.

## Graphical abstract

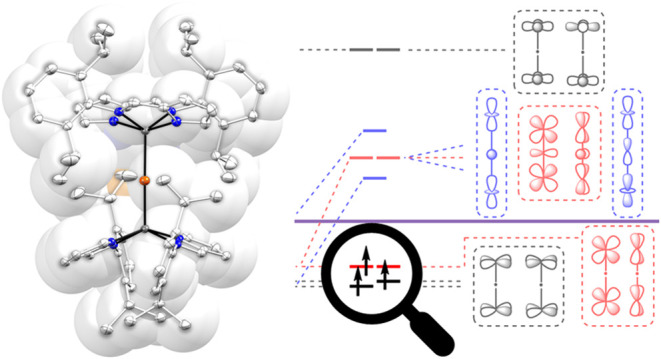

## Introduction

Multiple-bonded ligands almost invariably perturb the energetic ordering of metal d-orbitals in such a way as to enforce low-spin configurations. Still, molecular systems that overcome this situation could display electronic and magnetic phenomena that are otherwise unique to their solid-state analogs.^1^ As an illustration, the partially filled d-levels of vanadium­(III) phosphide (VP) render this compound paramagnetic.^2,3^ More generally, solid-state transition metal phosphides are a topical and widely occurring class of materials with applications in magnetism,^4,5^ semiconductors,^6^ catalysis,^7–11^ and battery technologies.^12–17^ In striking contrast, the chemistry of molecular phosphide complexes remains much more constrained. The difficulty of stabilizing the highly charged and oxidation-sensitive phosphide ligand (P^3–^) outside of purely inorganic, ionic lattices reflects both a limited selection of effectual phosphorus-atom transfer reagents as well as the requirement for the metal complex scaffolds to support kinetically stable phosphido multiple bonding, for instance by sterically suppressing degradation involving oligomerization, and other bond-activation pathways. Addressing both economic and long-term availability concerns, development of phosphide architectures based on Earth-abundant elements is desirable. Even so, the spatially contracted 3d orbitals of first-row transition metals generally result in poor π-overlap with the 3p valence orbitals of phosphorus, especially in comparison to the situation for 4d and 5d systems. In accord, second and third-row transition metal phosphide complexes are much more prevalent^18–51^ than their first-row counterparts (Figure 1),^52–57^ which, until recently, were largely limited to carbonyl cluster systems.^58–68^ For early transition metals, the low stability of [M≡P] and [M=P] multiple-bonds is further exacerbated by the inherent disparity between these hard Lewis acids and the soft phosphide ligand. On the other hand, this dissimilarity could give rise to exotic orbital interactions and electronic phenomena.

**Figure 1:**
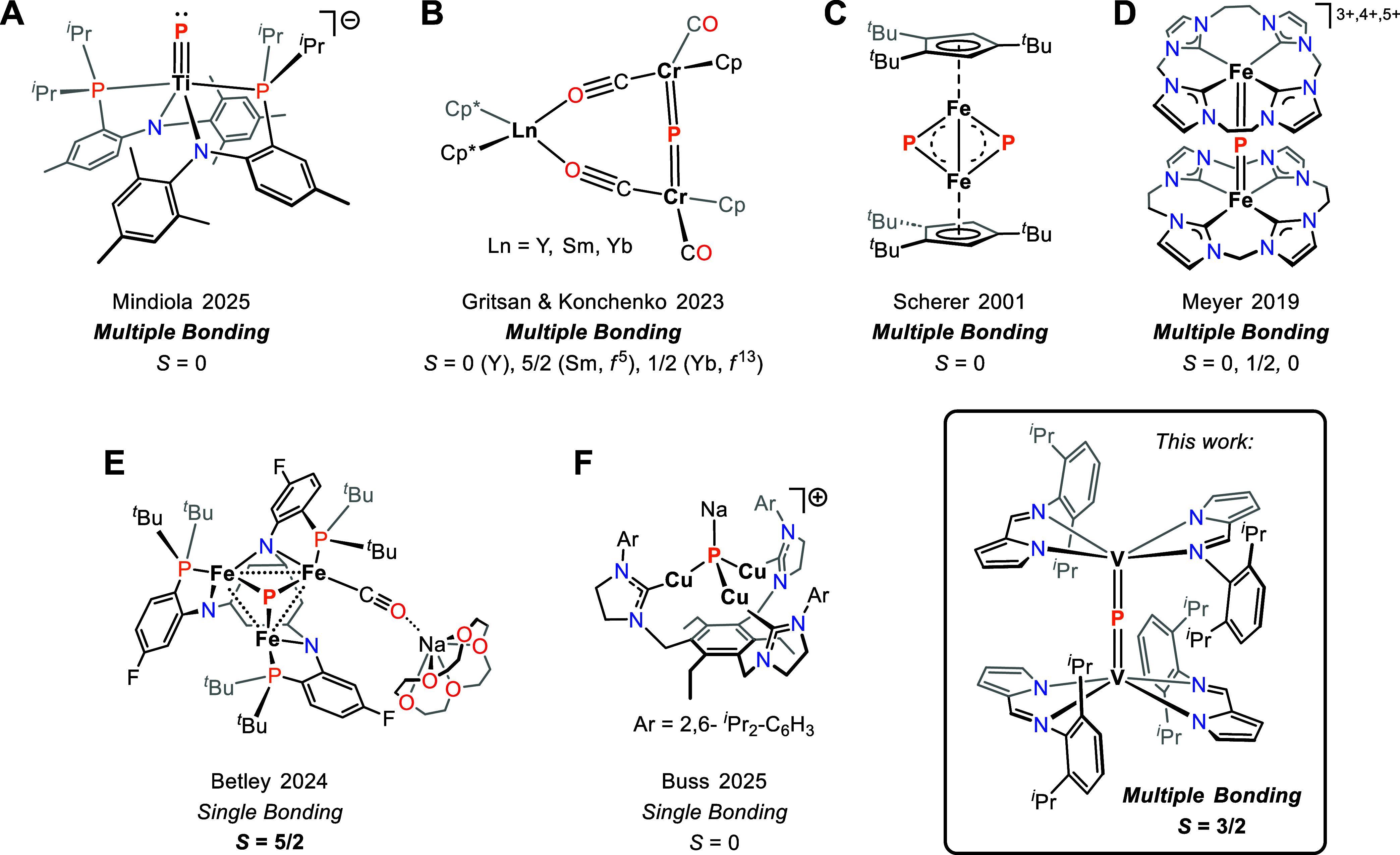
Selected examples of 3d phosphide complexes bearing terminal (A), μ_2_-bridging (B–D), and μ_3_-bridging (E–F) phosphide ligands along with our work describing a mixed-valent and high-spin [V=P=V] core.

Herein, we describe how an early transition metal complex based on vanadium exchanges a chloro ligand with Na­(OCP) to form a metastable [V^III^–PCO] fragment. Coordination of σ-donor ligands such as THF and DMAP induces a one-electron reductive elimination, transforming this precursor to V^II^ derivatives. When instead photolyzing the phosphaethynolate complex, decarbonylation and multiple-bond formation coupled with a similar reductive step lead to a mixed-valent [V=P=V] core comprising formal V^III^ and V^IV^ nodes. We probe the bonding and electronic structure of the cumulenic [V=P=V] core by combining Raman, UV–visible, and X-ray spectroscopies with magnetic studies. We scrutinize the underlying origin of the high-spin nature of the mixed-valent phosphide from first-principles theoretical arguments coupled with quantum chemical calculations, which uncover how a suitable balance between molecular symmetry, d-orbital occupation, and a sufficiently weak ligand field prompt the π-bonded complex to assume a high-spin electronic configuration with an *S*_T_ = 3/2 ground state.

## Results and Discussion

### Isolation of a [V^III^–PCO] Complex (**2**)

Decarbonylation of phosphaethynolate^69–72^ has become a versatile strategy to introduce a P atom in main group^73–86^ and transition metal systems,^87^ leading to various functional groups,^88–98^ closed-shell phosphide, and open-shell phosphinidene systems.^99–103^ In spite of these advances, well-defined molecular 3d metal phosphide functionalities remain uncommon. To obtain a platform for studying phosphide linkages in early transition metal chemistry, we prepared the vanadium precursor [(pyrNdipp)_2_VCl­(THF)] (**1**) from [VCl_3_(THF)_3_] and pyrrolylimine ligand Na­[pyrNdipp] (pyr = 2-pyrrolylmethylidene, dipp = 2,6-diisopropylphenyl).^104^ Upon metathesis of chloride precursor **1** with Na­(OCP)·2.5 dioxane in Et_2_O, we isolated phosphaethynolate complex, [(pyrNdipp)_2_V­(PCO)] (**2**) as dark red crystals in 48% yield after removal of volatiles and NaCl (Scheme 1). The choice of ether as solvent proved critical, due to the sensitivity of the V^III^ product toward Lewis bases (*vide infra*). The overall connectivity of **2** as well as the presence of a phosphorus-bound PCO^–^ ligand was unequivocally verified by single crystal X-ray diffraction (Figure 2). Complex **2** possesses a distorted five-coordinate structure, with an Addison geometry index of τ_5_ = 0.25, falling closer to an idealized square pyramidal geometry than a trigonal bipyramidal case.^105^ The PCO^–^ ligand is bound to vanadium at a right angle, whereas the V–P bond distance (2.3911(14) Å) is slightly shorter than in Mindiola’s connectively similar but four-coordinate [(nacnac)­V­(OAr)­(PCO)] complex (2.4502(4) Å, nacnac^–^ = {ArNC­(CH_3_)}_2_CH^–^; Ar = dipp).^106^ From IR spectroscopy, an intense resonance at 1911 cm^–1^ further confirmed the presence of a PCO^–^ ligand, vibrating at significantly higher energy than observed for typical PCO^–^ salts (1770 cm^–1^)^107^ as well as [(nacnac)­V­(OAr)­(PCO)] (1876 cm^–1^),^106^ suggesting a relatively strong interaction between the V^III^ node and the pseudohalide ligand. Furthermore, the paramagnetic nature of **2** is evident from paramagnetically shifted ^1^H NMR resonances (−13 to +9 ppm, FWHM = 10–430 Hz), an effective magnetic moment of μ_eff_ = 2.95 μ_B_ in solution (Evans’ method, toluene-*d*_8_, 298 K), as well as a computed spin-density distribution of 2.0 localized to the vanadium fragment (Figure S30).

**Figure 2:**
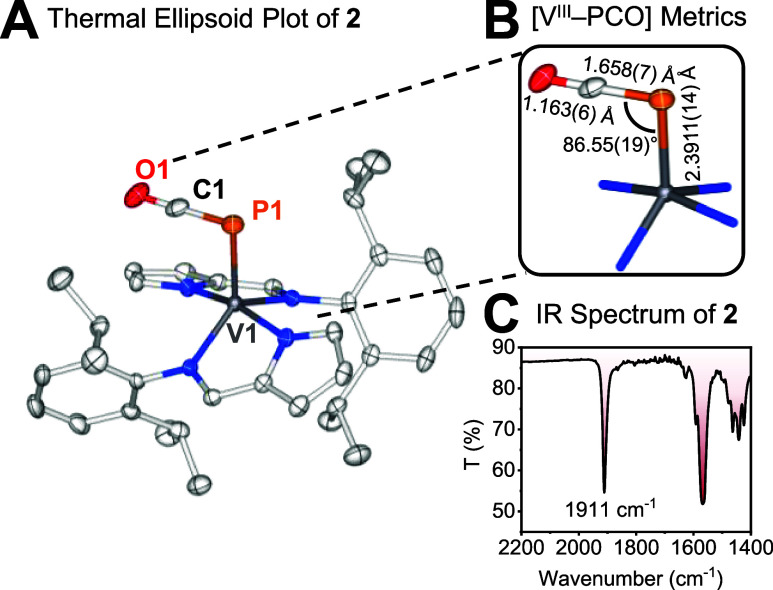
(A) X-ray crystallographic structure of **2** (50% probability, 100(2) K, H atoms omitted, orientational disorder of PCO^–^ group not displayed). (B) Salient metrics for [V^III^–PCO] fragment. (C) IR spectral data revealing PCO^–^ resonance.

**Scheme 1:**
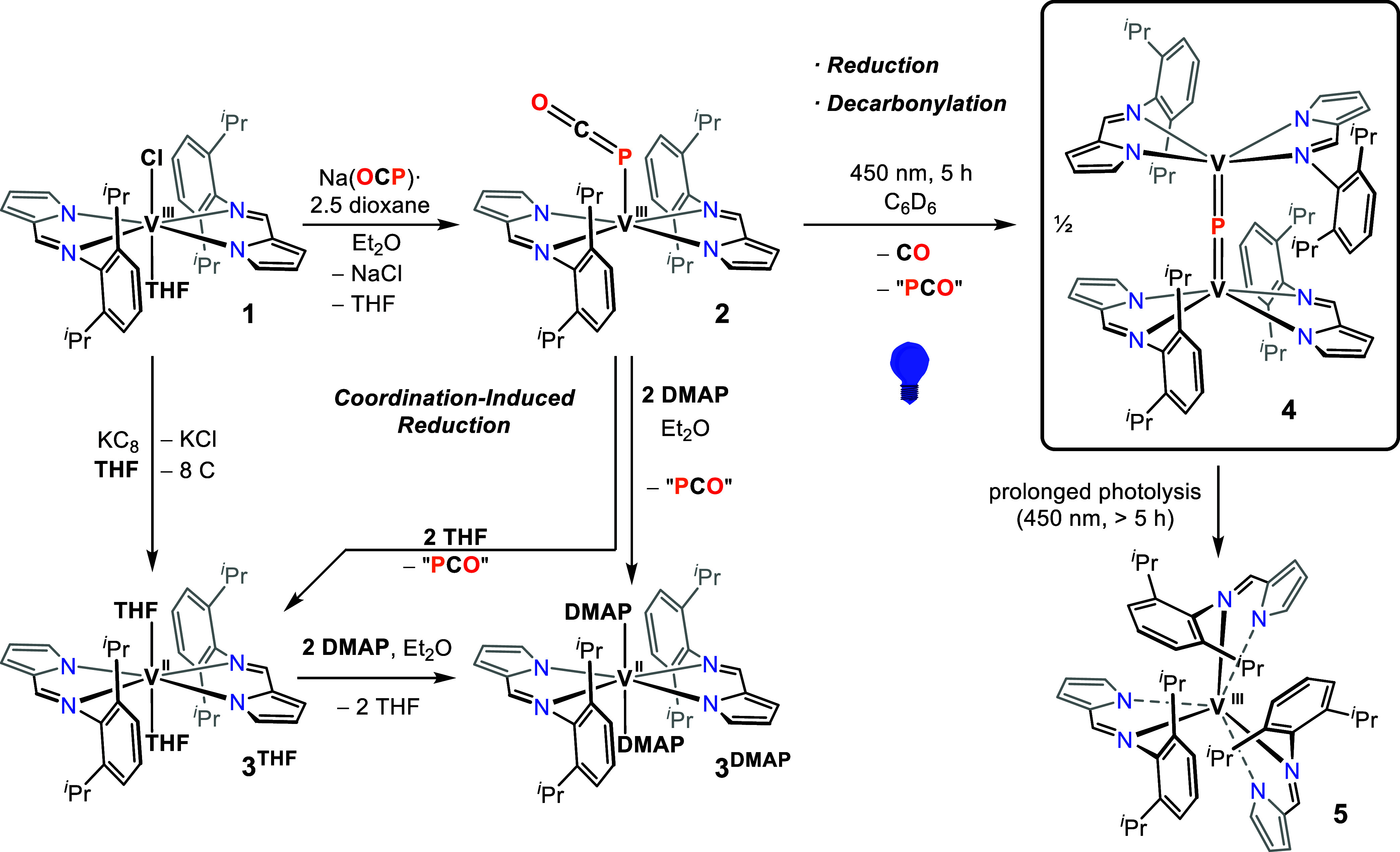
Na­(OCP) Converts **1** to a [V^III^–PCO] Complex **2**, THF and DMAP Convert **2** to V^II^ Complexes **3** ^**THF**^ **/3** ^**DMAP**^, Whereas Photolysis Converts **2** to a Mixed-Valent [V^III^=P=V^IV^] Phosphide **4**, and Prolonged Photolysis Converts **4** to V^III^ Complex **5**

### Coordination of Lewis Base Converts **2** to a V^II^ Complex (**3**)

Phosphaethynolate complex **2** readily undergoes one-electron reduction involving its V^III^ and PCO^–^ moieties. When dissolved in THF at room temperature, **2** transformed to the octahedral complex [(pyrNdipp)_2_V­(THF)_2_] (**3** ^**THF**^) within 5 min (Scheme 1), accompanied by formation of unidentified phosphorus products (Raman and ^31^P NMR analyses did not reveal red/black phosphorus or P_4_). In an independent synthetic route, **3** ^**THF**^ could be generated by reducing **1** with KC_8_ in THF. The overall connectivity of **3** ^**THF**^ was determined by X-ray crystallography, showing its *trans* geometry (Figure 3), but further characterization was hampered by the lability of the THF ligands. When instead dissolving **2** in Et_2_O and adding two equivalents of DMAP, formation of the analogous but more stable V^II^ *trans* complex [(pyrNdipp)_2_V­(DMAP)_2_] (**3** ^**DMAP**^) proceeded. Complex **3** ^**DMAP**^ exhibits broad ^1^H NMR resonances in the range +2 to +10 ppm, (FWHM = 220–1100 Hz), consistent with its paramagnetic nature.

**Figure 3:**
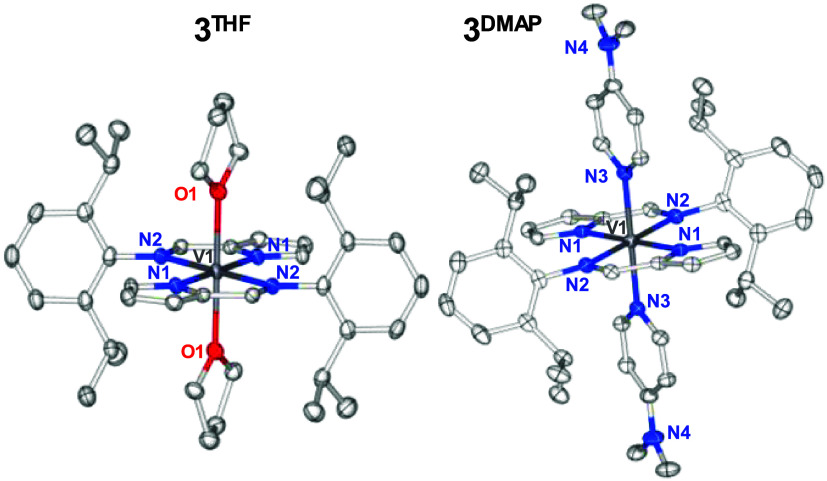
Molecular structures of **3** ^**THF**^ (left) and **3** ^**DMAP**^ (right) (50% probability, 100(2) K, H atoms omitted).

### Photolysis of **2** Generates a Mixed-Valent Vanadium Phosphide (**4**)

Considering the ability of **2** to undergo reduction to various V^II^ species, coupled with the possibility of a PCO^–^ ligand to undergo reductive decarbonylation to form a P^3–^ ligand, a range of different phosphide architectures could be accessed from the [V^III^–PCO] precursor. In view of its instability in coordinating solvents, we photolyzed a solution of **2** in C_6_D_6_ using 450 nm blue light (LED, 30 W, Scheme 1), matching an absorption maximum at 466 nm. This protocol generated the dinuclear and mixed-valent vanadium phosphide [(pyrNdipp)_2_V=P=V­(pyrNdipp)_2_] (**4**) as dark red crystals in 27% yield after fractional crystallization. Optimal irradiation times were found to be approximately 5 h, after which an octahedral byproduct, [V­(pyrNdipp)_3_] (**5**), began to dominate, hampering the isolation of pure samples of the kinetic product, **4**. The formation of **5** accelerates once the formation of **4** is complete, and hence carefully optimized irradiation times are essential. An X-ray crystallographic study unveiled the molecular structure of **4** (Figure 4A). Considering its molecular composition, **4** may tentatively be described as a mixed-valent species, comprising charge-separated [V_2_^II, V^] or [V_2_^III, IV^] nodes or alternatively a fully delocalized [V_2_^III1/2, III1/2^] system. Each of the vanadium centers exhibits qualitatively similar, distorted square pyramidal coordination (τ_5_ = 0.20, 0.23) with four nitrogen donor atoms forming a basal N_4_ plane and a bridging phosphide occupying the axial site along a linear V=P=V axis (Figure 4B). The vanadium centers are displaced away from the basal N_4_ plane (0.661 Å and 0.631 Å) toward the bridging phosphide, whereas the V=P bond distances are slightly dissimilar, 2.2118(10) and 2.2766(10) Å, likely reflecting crystal packing effects (Supporting Information, Figure S25) or a secondary electronic driving force (*vide infra*). The high symmetry of **4** is clearly visible when viewing the molecule along the V=P=V axis (Figure 4C); the conformation is essentially eclipsed, with pyrrole and imine nitrogens in opposing positions, resulting in an idealized *S*_4_ symmetry.

**Figure 4:**
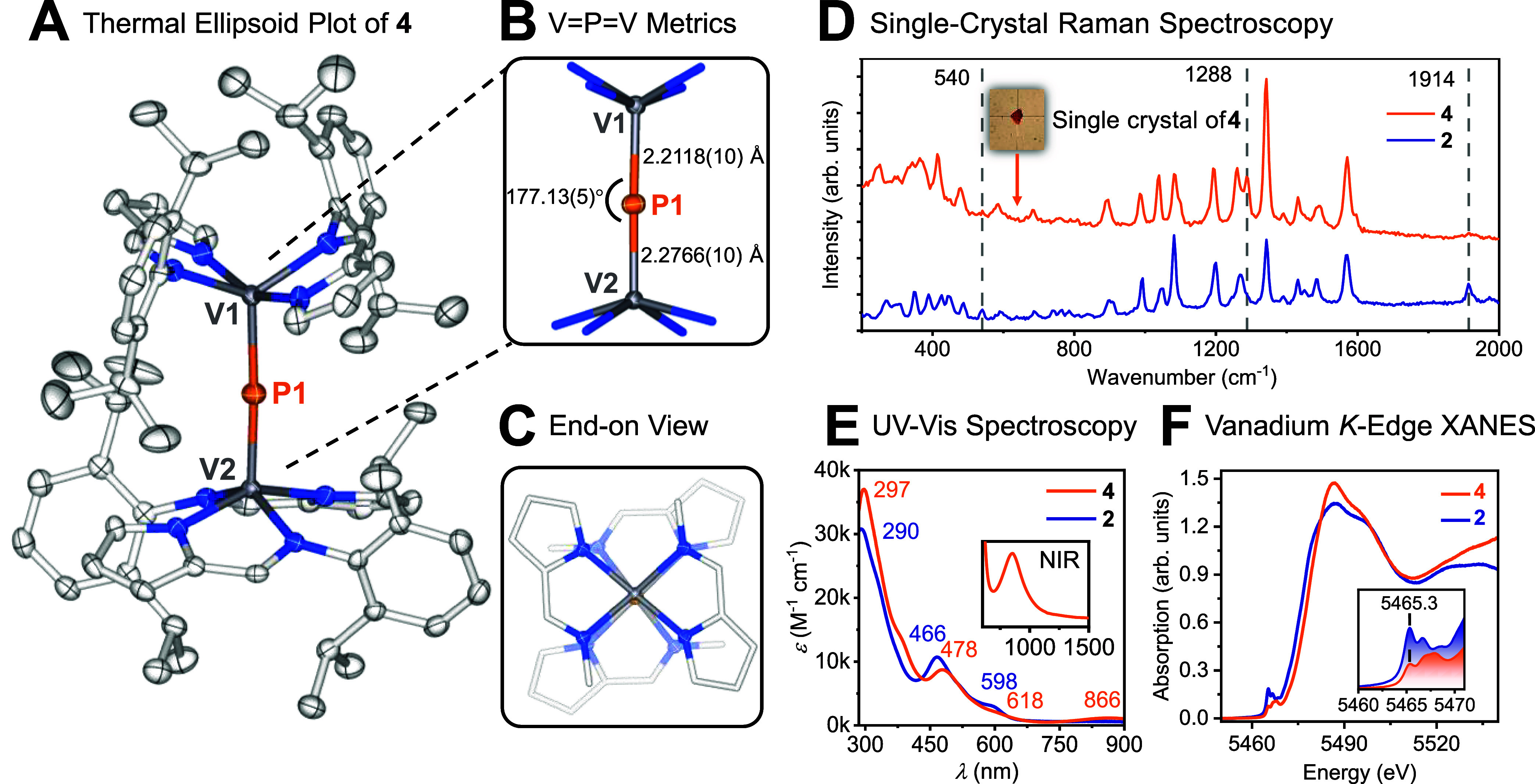
(A) Molecular structure of [V=P=V] complex **4** (50% probability, 150(2) K, H atoms omitted). (B) Metric data for the [V=P=V] moiety. (C) End-on view along [V=P=V] axis highlighting *S*_4_ symmetry (aromatic groups truncated for clarity). (D) Raman spectra of **2** and **4** recorded on single crystals (excitation at 488 nm). (E) UV–visible spectra of **2** (2.85 × 10^–5^ M), and **4** (6.14 × 10^–5^ M) in toluene; an intervalence charge-transfer band is present in **4** but not in **2** (inset). (F) Vanadium K-edge XANES spectra of **2** and **4**.

### Spectroscopic Studies of Phosphide Complex **4**

To gain more insight into the bonding and electronic structure of the V=P=V bridging core, we scrutinized complex **4** by various physical techniques. Initially, we conducted a Raman spectroscopic study on single crystals of **4** with excitation at 488 nm (Figure 4D). Considering the five-coordinate geometry about the vanadium centers in **4**, we used PCO^–^ complex **2** as a reference with a similar {VPN_4_} coordination. The complexes display essentially superimposable spectral features, except at 540 cm^–1^ (**2**), 1288 cm^–1^ (**4**), and 1914 cm^–1^ (**2**). We assign the unique resonances from **2** to a ν_(V–P)_ stretching mode as well as a ν_(PCO)_ mode close in energy to the IR-active vibration (Figure 2C), whereas the resonance at 1288 cm^–1^ identifies a ν_(V=P=V)_ breathing mode in **4**. Given their paramagnetic nature, neither **2** nor **4** displays an observable ^31^P NMR resonance. Turning to UV–visible spectroscopy, **2** and **4** exhibit strong absorptions at similar energy positions from 300–600 nm (ε > 2000 M^–1^ cm^–1^), ascribable to their similar ligand scaffolds (Figure 4E). However, the mixed-valent phosphide **4** displays a noticeable intervalence charge-transfer band at 866 nm (ε = 1700 M^–1^ cm^–1^), extending into the NIR, in line with a delocalized Robin-Day Class III description,^108^ indicative of a [V_2_^III1/2, III1/2^] valence form. Finally, to evaluate the oxidation state of the metal centers, we performed X-ray Absorption Near-Edge Structure (XANES) spectroscopy at the vanadium K-edge. The spectra display weak pre-edge features arising from quadrupole-allowed 1s → 3d transitions as well as a prominent rising edge due to more intense dipole-allowed 1s → 4p transitions (Figure 4F). The pre-edge region, involving directly the 3d orbitals, is sensitive to the oxidation state of vanadium.^109–115^ From the first derivatives of the spectra, the lowest-energy pre-edge features of **2** and **4** both occur at 5465.3 eV, suggesting a common spectroscopic oxidation state for these systems. On the other hand, the rising edge shifts to higher energy by nearly 2 eV when going from **2** to **4**, indicating a significantly stronger binding energy of the 1s electrons in the phosphide complex, **4**.

### Magnetic Data Reveal a High-Spin Configuration of **4**

The presence of formal d^1^ and d^2^ nodes in **4** could lead to either low-spin (*S*_T_ = 1/2) or high-spin (*S*_T_ = 3/2) electronic configurations. There is an apparent tendency for π-bonded phosphides (Figure 1) to induce spin-pairing, with chromium and iron phosphides **B** and **C** being diamagnetic,^53^ and the iron phosphide redox series **D** being either *S* = 0 or *S* = 1/2 spin systems.^55^ In a similar fashion, Cummins^18,42^ and Scheer^21^ reported that phosphide-bridged [(RR′N)_3_Mo=P=Mo­(NRR′)_3_] and [(RR′N)_3_W=P=W­(NRR′)_3_] complexes (RR′N^–^ = amido ligand), which contain three d-electrons, like **4**, possess magnetic moments consistent with a low-spin configuration and a single unpaired electron. In contrast, magnetization measurements on a polycrystalline sample of [V=P=V] complex **4** revealed an effective magnetic moment of 3.53 μ_B_ at 295 K (Figure 5). This value is significantly larger than the expected spin-only effective magnetic moment for a pair of magnetically uncoupled d^1^ (*S* = 1/2) and d^2^ (*S* = 1) ions of 3.32 μ_B_, necessitating physically unreasonable effective *g* factors exceeding 2.1. Upon cooling, the effective magnetic moment remains practically constant until 10 K, after which a steeper decrease results in a final value of 2.95 μ_B_ at 3 K. Given the temperature-insensitivity and the precedence of strong magnetic interactions across the phosphide bridge, the magnetic moment could be envisioned as originating from an energetically isolated *S*_T_ = 3/2 ground state. This interpretation necessitates *g*_eff_ = 1.82, which is well within the expected range for V^III^ systems.^116^ The low-temperature magnetization vs magnetic field data are well described by the *S*_T_ = 3/2 model, affording the best fit *g*_eff_ = 1.76(3) and axial zero-field splitting parameter, *D* = −2.7(8) cm^–1^. Collectively, the temperature- and magnetic field-dependence of the magnetization suggest the presence of a high-spin, isolated quartet ground state in **4**. Further spectroscopic evidence of an *S*_T_ = 3/2 species was obtained from an EPR study on a polycrystalline sample of **4**, showing a broad resonance below 14 K, which could be simulated using the values for *D* and *g*_eff_ from magnetometry (Supporting Information, Section 8).

**Figure 5:**
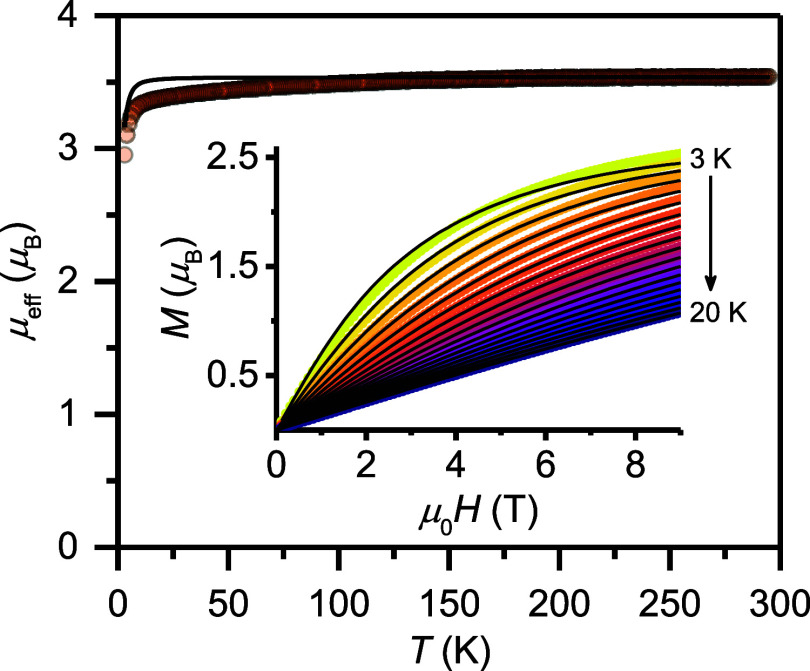
Temperature dependence of the effective magnetic moment, μ_eff_, obtained with μ_0_ *H* = 1.0 T on a polycrystalline sample of **4** in the temperature range from 3–295 K. The solid line depicts the calculated behavior for an isolated *S*_T_ = 3/2 state with *g*_eff_ = 1.82 and an axial zero-field splitting parameter, *D* = −2.7 cm^–1^. Inset: Magnetization vs magnetic field data obtained between 3 and 20 K in steps of 1 K. The black lines represent the best fit as described in the main text.

### Electronic Structure of Phosphide Complex **4**

To understand the electronic origin of the unique high-spin configuration of **4**, we dissect how d-orbitals split energetically in a tetragonal [M=P=M] unit (Figure 6A).^117–121^ The system may be constructed by initially placing two square-planar metal fragments along the *z*-axis. In this framework, the metal d_*x* ^2^–*y* ^2^_ orbitals are strongly antibonding, high in energy, and vacant, from interacting with the pyrNdipp^–^ ligands, and do not play a role in forming bonding interactions along the [V=P=V] core. On the other hand, the d_*z* ^2^_, d_*xz*_, d_*yz*_, and d_*xy*_ orbitals may be ordered based on their symmetry (σ, σ*, π, π*, δ, δ*, respectively). This does not imply any significant intermetallic overlap or interaction, but simply offers a useful grouping of these orbitals. When placing the bridging phosphide symmetrically between the metal nodes, the metal-based σ-, σ*-, and π-groups shift higher in energy as a result of interacting with filled 3s and 3p orbitals of phosphorus to form bonding and antibonding combinations (multiple-bond group), whereas the δ, δ*, and π* groups remain nonbonding (ligand field group). This leaves the native ligand-field splitting of the square planar fragments to determine the energetic separation within the metallocentric ligand-field group. In essence, π-donation within the *xy*-plane spanned by the four equatorial ligands shifts the δ and δ* groups higher in energy, whereas π-donation perpendicular to this plane affects the π* group. Considering the structure of the pyrNdipp^–^ ligands, the π* group should reside energetically above δ and δ*.

**Figure 6:**
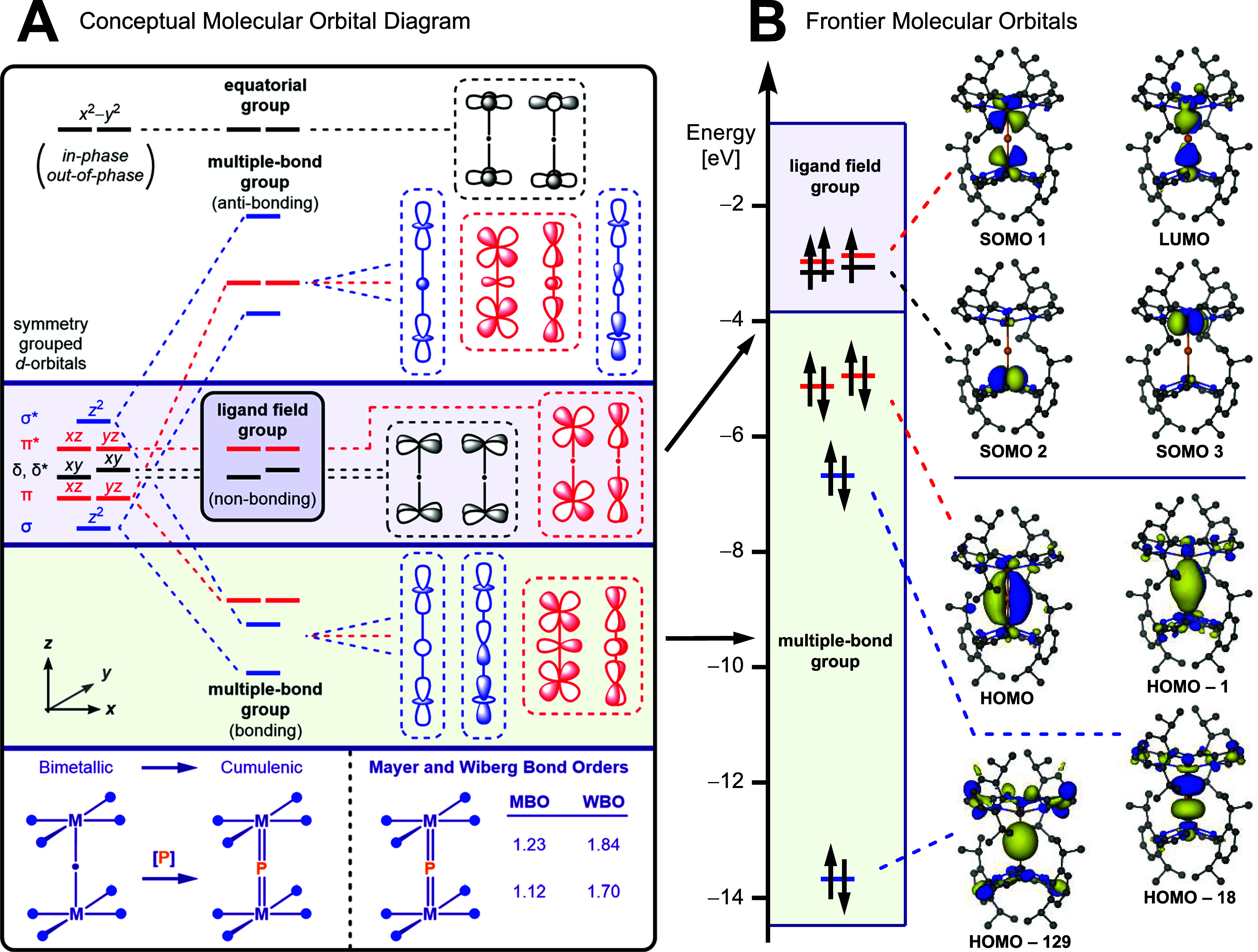
(A) Qualitative molecular orbital diagram for a μ_2_-bridging phosphide complex derived from two square-planar complexes. (B) Frontier quasi-restricted orbitals (QROs) of **4** depicting the nonbonding ligand field group housing three unpaired electrons as well as the bonding combinations of the multiple bond group forming the delocalized [V=P=V] σ- and π-manifolds. Orbitals are plotted at an isosurface of 0.03 au and generated at the TPSSh-D3/def2-TZVP level of theory.

To describe the electronic structure of **4** in more detail, we performed a density functional theory (DFT) study, using PBE0-D3/def2-TZVP­(-f) for geometry optimizations and TPSSh-D3/def2-TZVP for single-point calculations (Figure 6B). In the Supporting Information, Section 10, we also trace the interplay between the spin-state and the nature of the metal fragment for hypothetical model complexes. For complex **4**, the chosen level of theory reproduces the X-ray crystallographic metrics well for a high-spin, *S*_T_ = 3/2 quartet state (Table S3). In harmony with the magnetization data and interpretation, the quartet state is calculated to be 10.8 kcal mol^–1^ lower in energy as compared to a doublet configuration (Figure S27). When inspecting the calculated quasi-restricted orbitals (QROs), HOMO–129 and HOMO–18 depict the 3s and 3p_*z*_ orbitals of phosphorus forming σ-bonding combinations with the vanadium d_*z* ^2^_ orbitals. At higher energy, HOMO–1 and HOMO represent the 3p_*x*_ and 3p_*y*_ orbitals of phosphorus forming orthogonal and delocalized π-components of the [V=P=V] manifold, seen as the bonding combinations within the multiple-bond group in Figure 6A. Still higher in energy, the ligand field group is represented by the SOMO1, SOMO2, SOMO3, and LUMO. The exact energetic ordering of these four orbitals differs slightly from the qualitative picture, given the influence of the [pyrNdipp]^−^ ligands on the finer details of the ligand field splitting. In line with the cumulenic nature of the [V=P=V] linkage, Mayer Bond Orders (1.12–1.23) and Wiberg Bond Orders (1.70–1.84) portray slightly asymmetrical V=P multiple-bond character. The nonequivalence of these bonds reflects a Jahn–Teller distortion that lifts the degeneracy of the π* group of d-orbitals, which would otherwise result within a perfectly *S*_4_ symmetrical system. In line with this slight distortion, the d_*xy*_-type orbitals (SOMO2 and SOMO3) are each predominantly concentrated to a single metal center rather than being perfectly delocalized. Lastly, the equal distribution of SOMO1 across the two vanadium centers provides computational evidence for **4** being a fully delocalized, mixed-valent Robin-Day Class III species. In accordance, time-dependent DFT (Figure S38) reveals a dominant contribution from the transition SOMO3 → LUMO, suggesting the intervalence charge-transfer band (Figure 4E) to change the charge distribution between [V_2_^III1/2, III1/2^] and [V_2_^III, IV^] valence forms.

## Conclusions

In summary, we have synthesized a unique example of a high-spin and multiple-bonded transition metal phosphide **4**. Cumulenic phosphido ligation connects formal V^III^ and V^IV^ nodes into a mixed-valent [V=P=V] motif. Central to the formation of **4** was a multitude of mechanistic steps (reduction, decarbonylation, multiple-bond formation) proceeding when V^III^ phosphaethynolate precursor **2** was photolyzed. Notably, our study reveals how the PCO^–^ moiety not only serves as a phosphorus-atom transfer reagent, but also is able to act as a one-electron reductant for the formation of V^II^ species **3** ^**THF**^ and **3** ^**DMAP**^. Combining Raman, UV–visible, and vanadium K-edge XANES spectroscopy, we probe the bonding and electronic structure of **4**, unveiling a fully delocalized Robin-Day Class III d-manifold and an intermediate oxidation state of vanadium. Magnetometric measurements reveal a high-spin, *S*_T_ = 3/2 ground state of **4**. From theoretical studies, we trace this singular electronic situation to the weak-field nature of the vanadium 3d nodes, overcoming the spin-pairing energy that forces classical phosphide systems to assume low-spin configurations. Given its ^4^ *E*_g_ ground term, a Jahn–Teller distortion reduces the orbital degeneracy of **4**, resulting in a subtle deviation of this highly symmetrical molecule from idealized *S*_4_ symmetry. Overall, we not only present the first example of a molecular vanadium phosphide, but also lay out a strategy to maximize the magnetic moment of multiple-bonded transition metal complexes, leveraging ligand-field engineering and optimized d-orbital occupation: In essence, the low ligand-field splitting typical of a 3d transition metal coupled with the limited d-electron population of an early transition metal leads to optimal occupation of the d-states of a phosphide complex. Guided by this conceptual design principle, other rare high-spin systems are currently underway.

## Supplementary Material



## Notes

Synthetic procedures; NMR, IR, UV–vis, Raman, XANES spectroscopic data, magnetic data, crystallographic tables, and computational studies (PDF)

## ∇

European Research Council Executive Agency, 1049 Bruxelles/Brussel, Belgium

## Abbreviations

Ar: aryl
Cp: cyclopentadienyl
Cp*: pentamethylcyclopentadienyl
dipp: 2,6-diisopropylphenyl
DMAP: 4-dimethylaminopyridine
Et: ethyl
^*i*^Pr: isopropyl
pyr: 2-pyrrolylmethylidene
^*t*^Bu: *tert*-butyl
THF: tetrahydrofuran
