# Supplementary material for: A Mixed-Valent and High-Spin Vanadium Phosphide
Source: J Am Chem Soc. 2026 Feb 27;148(9):9629–39. doi: 10.1021/jacs.5c20647 (PMC12983316; doi:10.1021/jacs.5c20647)
Supplement: Supplementary file 1 [file ja5c20647_si_001.pdf]

Electronic Supplementary Information

For

A Mixed-Valent and High-Spin Vanadium Phosphide

Aswin Chandran,[a] Christian Sandoval-Pauker,[b] Balazs Pinter,[c,†] Tom Vosch,[d] Fabrice Wilhelm,[e] Andrei Rogalev,[e] Kasper S. Pedersen,[f] Anders Reinholdt[a,*]

[a] Department of Chemistry, Lund University, Naturvetarvägen 22, 22100 Lund, Sweden

[b] Department of Chemical and Biomolecular Engineering, Rice University, 6100 Main Street, Houston, TX 770052, USA

[c] Department of Chemistry and Biochemistry, University of Texas at El Paso, El Paso, TX 79968, USA

[d] Department of Chemistry, University of Copenhagen, Universitetsparken 5, DK-2100, Denmark

[e] ESRF - The European Synchrotron Radiation Facility, CS 40220, 38043 Grenoble, Cedex 9, France

[f] Department of Chemistry, Technical University of Denmark, Kemitorvet, DK-2800 Kgs. Lyngby, Denmark

Table of Contents

1. General considerations ***4***

2. Synthesis of complexes ***7***

*2.1 Synthesis of [(pyrNdipp)$_2$VCl(THF)] (**1**)* *7*

*2.2 Synthesis of [(pyrNdipp)$_2$V(PCO)] (**2**)* *8*

*2.3 Synthesis of [(pyrNdipp)$_2$V(DMAP)$_2$] (**3**^{DMAP})* *9*

*2.4 Synthesis of [(pyrNdipp)$_2$V(THF)$_2$] (**3**^{THF})* *10*

*2.5 Synthesis of [(pyrNdipp)$_2$V=P=V(pyrNdipp)$_2$] (**4**)* *11*

*2.6 Synthesis of [(pyrNdipp)$_3$V] (**5**)* *13*

3. NMR Spectroscopy ***14***

*3.1 ^{1}H NMR spectrum of [(pyrNdipp)$_2$VCl(THF)] (**1**)* *14*

*3.2 ^{1}H NMR spectrum of [(pyrNdipp)$_2$V(PCO)] (**2**)* *15*

*3.3 ^{1}H NMR spectrum of [(pyrNdipp)$_2$V(DMAP)$_2$] (**3**^{DMAP})* *16*

*3.4 ^{1}H NMR spectrum of [(pyrNdipp)$_2$V=P=V(pyrNdipp)$_2$] (**4**)* *17*

*3.5 ^{1}H NMR spectrum of [(pyrNdipp)$_3$V] (**5**)* *18*

4. IR Spectroscopy ***19***

*4.1 IR Spectrum of [(pyrNdipp)$_2$VCl(THF)] (**1**)* *19*

*4.2 IR Spectrum of [(pyrNdipp)$_2$V(PCO)] (**2**)* *20*

*4.3 IR Spectrum of [(pyrNdipp)$_2$V(DMAP)$_2$] (**3**^{DMAP})* *21*

*4.4 IR Spectrum of [(pyrNdipp)$_2$V=P=V(pyrNdipp)$_2$] (**4**)* *22*

*4.5 IR Spectrum of [(pyrNdipp)$_3$V] (**5**)* *23*

5. UV-Vis Spectroscopy ***24***

*5.1 UV-Vis spectral data for [(pyrNdipp)$_2$VCl(THF)] (**1**)* *24*

*5.2 UV-Vis spectral data for [(pyrNdipp)$_2$V(PCO)] (**2**)* *25*

*5.3 UV-Vis spectral data for [(pyrNdipp)$_2$V=P=V(pyrNdipp)$_2$] (**4**)* *26*

6. Raman Spectroscopy ***28***

*6.1 Raman spectral data for [(pyrNdipp)$_2$V(PCO)] (**2**)* *28*

*6.2 Raman spectral data for [(pyrNdipp)$_2$V=P=V(pyrNdipp)$_2$] (**4**)* *29*

*6.3 Raman spectra of [(pyrNdipp)$_2$V(PCO)] (**2**) compared with [(pyrNdipp)$_2$V=P=V(pyrNdipp)$_2$] (**4**)* *30*

7. X-ray Absorption Spectroscopy ***31***

8. EPR Spectroscopy ***33***

9. Crystallographic Data 34
9.1 Crystallographic Tables 34
9.2 X-ray Molecular Structures 36
*9.2.1 [(pyrNdipp)$_2$V(PCO)] (**2**) 36*
*9.2.2 [(pyrNdipp)$_2$V(DMAP)$_2$] (**3**^{DMAP}) 37*
*9.2.3 [(pyrNdipp)$_2$V(THF)$_2$] (**3**^{THF}) 38*
*9.2.4 [(pyrNdipp)$_2$V=P=V(pyrNdipp)$_2$] (**4**) 39*
*9.2.5 [(pyrNdipp)$_3$V] (**5**) 41*
10. Computational Studies 42
10.1 Computational methodology 42
10.2 Computational data 43
10.3 TD-DFT Calculations 53
10.4 Reaction Energies 53
*10.5 Relevant Quasi-restricted Orbitals of [(pyrNdipp)$_2$V=P=V(pyrNdipp)$_2$] (**4**) (S=3/2) together with their Mulliken orbital composition analysis. 54*
10.6 Relevant Quasi-restricted Orbitals of [(pyrNdipp)$_2$Nb=P=Nb(pyrNdipp)$_2$] (S = 3/2) 56
10.7 Relevant Quasi-restricted Orbitals of [(pyrNdipp)$_2$Ta=P=Ta(pyrNdipp)$_2$] (S = 3/2) 57
10.8 Relevant Quasi-restricted Orbitals of [(tBuPhN)$_3$Cr=P=Cr(NtBuPh)$_3$] (S = 1/2) 58
10.9 Relevant Quasi-restricted Orbitals of [(tBuPhN)$_3$Cr=P=Cr(NtBuPh)$_3$] (S = 3/2) 59
10.10 Relevant Quasi-restricted Orbitals of [(tBuPhN)$_3$Mo=P=Mo(NtBuPh)$_3$] (S = 1/2) 60
10.11 Relevant Quasi-restricted Orbitals of [(tBuPhN)$_3$Mo=P=Mo(NtBuPh)$_3$] (S = 3/2) 61
10.12 Relevant Quasi-restricted Orbitals of [(tBuPhN)$_3$W=P=W(NtBuPh)$_3$] (S = 1/2) 62
10.13 Relevant Quasi-restricted Orbitals of [(tBuPhN)$_3$W=P=W(NtBuPh)$_3$] (S = 3/2) 63
10.14 Relevant Quasi-restricted Orbitals of [(ArO)(nacnac)V=P=V(nacnac)(OAr)] (S = 3/2) 64
10.15 Cartesian coordinates 65
11. References 108

1. General considerations

All manipulations were performed under inert conditions using a N_2 filled Vigor glovebox (O_2, H_2O < 1 ppm). Hexane (Fisher Scientific) and toluene (Fisher Scientific) were collected from (MBraun SPS 800) solvent purifier system and stored over 4 Å molecular sieves (Fisher Scientific) to remove any residual water. Tetrahydrofuran (Sigma Aldrich), benzene (Sigma Aldrich) and diethyl ether (Sigma Aldrich) were distilled from sodium-benzophenone diketyl radical, degassed by freeze-pump-thaw cycles *in vacuo*, and stored over 4 Å molecular sieves (Fisher Scientific) prior to use. Pentane was subjected to freeze-pump-thaw cycles and subsequently stored over 4 Å molecular sieves. Benzene-d_6 (Sigma Aldrich) was dried over potassium mirror overnight, sublimed by trap-to-trap transfer *in vacuo*, and degassed by freeze-pump-thaw cycles. [$VCl_3(THF)_3$],[1] Na(OCP) · 2.5 dioxane,[2] and KC_8,[3] were prepared according to the reported procedures. [$(pyrNdipp)_2VCl(THF)$] was prepared following procedure described in literature,[4] with H(pyrNdipp) (N-(2,6-diisopropylphenyl)-1-(pyrrol-2-yl)methanimine) made using pyrrole-2-carboxaldehyde (98%, Sigma Aldrich), and 2,6-diisopropylaniline (90%, Sigma Aldrich). Sodium hydride (90%) was purchased from Sigma Aldrich and used as received.

^{1}H NMR spectroscopic studies were carried out using Bruker 400 MHz, 500 MHz spectrometers equipped with J. Young NMR tubes (complexes are paramagnetic in nature, broad signals). ^{1}H NMR chemical shifts are referenced to residual solvent signals (C_6D_6: ^{1}H: 7.16 ppm).

IR spectroscopic studies were carried out using a Bruker ALPHA II spectrometer with solid samples ground with KBr and pressed into pellets. To prevent decomposition, the analysis chamber was flushed with nitrogen during measurements.

Solution state magnetic susceptibility was measured by Evans' method on samples dissolved in suitable solvent with deuterated solvent being also used as external standard inside a capillary. Correction for diamagnetism were made using tabulated Pascal constants.[5]

X-band EPR studies were carried out using a Bruker Elexsys E500 EPR spectrometer. The experiments were carried out on flame-sealed capillaries containing [$(pyrNdipp)_2V{=}P{=}V(pyrNdipp)_2$] (**4**), stored inside quartz tubes, and cooled from 292 K to 5 K using a liquid helium cooled cryostat. Simulation of the spectral

data, based upon magnetometric data, was carried out using the program SIM written by H. Weihe, University of Copenhagen.

Crystallographic studies were carried out on single crystals, which were coated with NVH oil, mounted at the end of a MiTeGen Dual-Thickness Micromount, and placed in the nitrogen cold stream of the diffractometer. Data were collected and processed using Oxford Diffraction Xcalibur Eos diffractometer (Mo Kα radiation), operated via CrysAlisPro software.[6] The crystal structures were solved using SHELXT[7] (intrinsic phasing) and refined using SHELXL-2018[8] (least squares), with data processing carried out in Olex2.8.[9] Non-hydrogen atoms were refined anisotropically. Hydrogen atoms were placed at calculated positions and refined as riding atoms with isotropic displacement parameters (U_{iso} = 1.2 U_{eq} of the parent atom for CH and CH_2 groups, and U_{iso} = 1.5 U_{eq} of the parent atom for CH_3 groups). Severely disordered solvent regions had diffuse electron density modelled with the use solvent mask option in Olex2 (SQUEEZE).[10] Complex $\mathbf{3}^{\mathbf{DMAP}}$ displays solvent-accessible voids of 177.6 $Å^3$ with an estimated electron count of 48.4 (toluene, 50 electrons). Complex **4** displays solvent-accessible voids of 560 $Å^3$ with an estimated electron count of 92.0 (2×Et_2O, 84 electrons).

Elemental analyses were carried out by Mikroanalytisches Laboratorium Kolbe (Oberhausen, Germany). After being in transit (Sweden to Germany, several days), the samples were transferred directly into a glovebox at the analysis facility. The extremely air- and moisture-sensitive low-valent, early transition metal complexes reported herein generally proved incompatible with this best-practices protocol ($\mathbf{3}^{\mathbf{DMAP}}$, **4**, and **5**), resulting in unsatisfactory CHN analyses, after several attempts.

UV-vis spectroscopic studies were carried out using an Agilent Technologies Cary 60 Spectrometer or a Perkin Elmer lambda 1050 UV vis NIR Spectrometer equipped with 1.00 cm quartz cuvettes sealed with Teflon stoppers and electrical tape.

Raman spectroscopy studies were performed using a micro-Raman setup in back-scattering geometry. The samples were sealed in a glass capillary under an inert atmosphere. The 488 nm line of an Ar-ion laser (CVI Melles Griot 35MAP431-200) was used (11 µW on top of the objective). The beam was focused in an inverted confocal microscope (Olympus IX71) by an Olympus 100X, 1.4 N.A. oil immersion objective into a diffraction-limited spot. A LL01-488-12.5 and FF02-485/20-25 filter (Semrock) were used to clean the laser light in the excitation path. Raman spectra were recorded using a Princeton Instruments SPEC 10:100 B/LN- eXcelon CCD

detector and an SP 2356 spectrometer with a 600 grooves per millimetre grating. A 30:70 beam splitter (XF122 Omega Optical) was used instead of a dichroic mirror in the microscope. A LP02-488RU-25 filter (Semrock) was used to block the laser light in the detection path. X-axis calibration was performed with a Neon spectral calibration lamp (6032 Newport). No Y-axis corrections or background removal procedures were performed. Raman spectra are offset with a constant value for display purposes.

X-Ray absorption spectroscopic studies have been performed at the ID12 of the European Synchrotron Radiation Facility (ESRF).[11] For the Vanadium K-edge experiments, the x-ray source was the first harmonic of HELIOS-II-type helical undulator (HU-52) in pure helical mode. The advantage of using helical mode is that the content of the higher order harmonics is strongly reduced, since only the fundamental harmonic is emitted on the undulator axis. The x-ray beam was monochromatized using a fixed-exit double-crystal monochromator equipped with a pair of Si(111) crystals. Powdered samples were mounted inside argon filled glove-box into vacuum tight sample holders covered with 13-μm thick Kapton foil. The spectra were recorded at ambient temperature using total fluorescence yield detection mode. The x-ray fluorescence photons from the samples were collected in the back-scattering geometry, through the Kapton window, using Si photodiodes. Vanadium K-edge spectra of VCl_2 and VCl_3 were adapted from literature.[12,13] For the sake of comparison, the spectra were normalized using standard procedures and corrected for reabsorption effect.

Magnetization Data and Analysis - The magnetization measurements were performed using the vibrating sample magnetometry option on a Quantum Design Dynacool Physical Property Measurement System (PPMS) in the temperature range from 3 K to 295 K, in dc magnetic fields up to $\mu_0 H$ = 9 T. Polycrystalline samples were loaded in standard QuantumDesign powder capsules inside an argon-filled glovebox and attached to standard Quantum Design brass powder capsule sample holders. The modelling of the data employed the spin-Hamiltonian $\hat{\mathcal{H}} = \mu_B \mu_0 g\, \mathbf{H} \cdot \hat{\mathbf{S}}_T + D\,(\hat{S}_{T,z} - S_T(S_T + 1)/3)$ pertaining to the S_T = 3/2 state, and was conducted using MagProp[14] as included in the DAVE programme suite.[15]

2. Synthesis of complexes

2.1 Synthesis of [(pyrNdipp)$_2$VCl(THF)] (1)

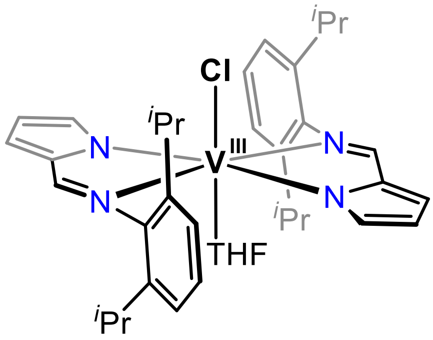

We prepared [(pyrNdipp)$_2$VCl(THF)] (**1**) according to a modified literature procedure.[4] Inside the glovebox, an excess of sodium hydride (70.7 mg, 2.94 mmol, 2.2 eq.) was added to a solution of H(pyrNdipp) (680.8 mg, 2.677 mmol, 2.0 eq.) in 10 mL THF in a glass vial. The heterogeneous mixture was stirred for 2 h at room temperature until no visible hydrogen gas bubbles were observed. The solution containing Na(pyrNdipp) was filtered through celite to remove excess sodium hydride and added to [VCl$_3$(THF)$_3$] (500.0 mg, 1.338 mmol, 1.0 eq.). A sudden color change from light red to dark red was observed. The reaction mixture was stirred at room temperature for 20 h and filtered through celite to remove NaCl. The solvent was removed under reduced pressure, leaving dark red solid product [(pyrNdipp)$_2$VCl(THF)] (**1**) which was recrystallized from a saturated solution of THF/hexane (1:3) mixture at -30 °C. The isolated crystals were washed with cold hexane and dried in vacuo. Yield of [(pyrNdipp)$_2$VCl(THF)] (**1**): 410 mg, 0.616 mmol, 46.1% based on [VCl$_3$(THF)$_3$]. **Elemental analysis**: Calculated – $C_{38}H_{50}ClN_4OV$ (665.24 g mol^{-1}). C, 68.61%; H, 7.58%; N, 8.42%, Found – C, 68.49%; H, 7.39%; N, 8.34%. **^{1}H NMR**: (400 MHz, C_6D_6) δ (ppm): 9.70 (FWHM = 34.6 Hz), 6.46 (FWHM = 71.5 Hz), 5.79 (FWHM = 63.6 Hz), 4.68 (FWHM = 371.1 Hz), 1.49 (FWHM = 29.1 Hz), 1.37 (FWHM = 20.5 Hz), -3.36 (FWHM = 594.1 Hz), -6.55 (FWHM = 348.3 Hz). **UV-Vis:** Toluene, λ_{max} [nm, ε (max/sh, M^{-1} cm^{-1})] 298 (max, 20000), 469 (max, 9600), 592 (max, 1900). **IR:** (KBr) ν (cm^{-1}) 1571 (ν_{imine}).

2.2 Synthesis of [(pyrNdipp)$_2$V(PCO)] (2)

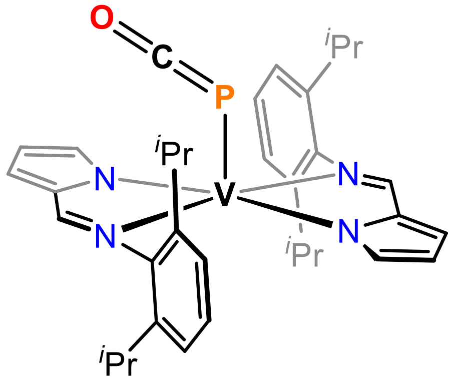

A suspension of Na(OCP) · 2.5 dioxane (100 mg, 0.331 mmol, 1.1 eq.) in 2 mL diethyl ether was added to [(pyrNdipp)$_2$VCl(THF)] (**1**) (200 mg, 0.301 mmol. 1.0 eq.) and stirred at room temperature in the absence of light over 20 h. A color change from red to dark red was observed. The solution was filtered through celite to remove the NaCl formed. The solvent and dioxane were then removed under vacuum. The product [(pyrNdipp)$_2$V(PCO)]·Et$_2$O (**2**) was crystallized as dark red block crystals by concentrating a diethyl ether solution of **2**, using toluene as sorbent (–30 °C, 2 days). The crystalline product was washed with cold pentane and dried in vacuo (Yield: 90 mg, 0.130 mmol, 43.3%, based on **1**). Single crystals suitable for X-ray diffraction were grown by the same procedure used for synthesizing **2**. **Elemental analysis**: [(pyrNdipp)$_2$V(PCO)]·Et$_2$O Calculated – $C_{39}H_{52}N_4O_2PV$ (690.79 g/mol). C, 67.81%; H, 7.59%; N, 8.11%; P, 4.48%; Found – C, 67.77%; H, 7.57%; N, 8.07%; P, 4.51%.**^{1}H NMR**: (400 MHz, C_6D_6) δ (ppm): 8.96 (FWHM = 44.1 Hz), 6.71 (FWHM = 80.7 Hz), 5.86 (FWHM = 85.2 Hz), 1.65 (FWHM = 237.6 Hz), 1.23 (FWHM = 9.6 Hz), 0.95 (FWHM = 281.6 Hz), -10.98 (FWHM = 270.8 Hz), -13.09 (FWHM = 428.0 Hz). **UV-Vis:** Toluene, λ_{max} [nm, ε (max/sh, M^{-1} cm^{-1})] 290 (max, 31000), 466 (max, 11000), 598 (sh, 2900). **Magnetic moment**, μ_{eff} (Evans' method, Toluene-d_8, 298 K): 2.95 μ_B. **IR:** (KBr) ν (cm^{-1}) 1911 (ν_{PCO}), 1567 (ν_{Imine}).

Stability of 2 toward CO. In a J Young NMR tube, complex **2** (6.7 mg, 11 µmol) was dissolved in 0.5 ml C_6D_6, subjected to three freeze-pump-thaw cycles, and finally backfilled with a 1 bar of CO (headspace 2.5 ml, 100 µmol gas). The reaction mixture was monitored by ^{1}H NMR spectroscopy before, and three hours after, addition of CO; both spectra showed characteristic resonances from **2**, and no indications of a reaction with CO.

2.3 Synthesis of [(pyrNdipp)$_2$V(DMAP)$_2$] (3^{DMAP})

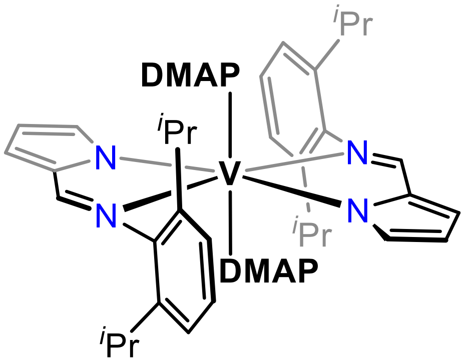

Method 1: Crystals of [(pyrNdipp)$_2$V(PCO)]·Et$_2$O (**2**) (10.1 mg, 0.015 mmol, 1.0 eq.) were dissolved in C_6D_6 in a vial, and DMAP (4.5 mg, 0.037 mmol, 2.5 eq.) was added to the solution at room temperature. The crude mixture was analyzed by ^{1}H NMR spectroscopy to identify the product. The solvent was removed under vacuum, and product (**3^{DMAP}**) was crystallized from saturated diethyl ether, using toluene as sorbent (–30 °C) as dark red block crystals, suitable for single crystal X-ray diffraction.

Method 2: A suspension of KC_8 (10 mg, 0.074 mmol, 2.5 eq.) in 2 mL THF was added to [(pyrNdipp)$_2$VCl(THF)] (**1**) (20 mg, 0.030 mmol. 1.0 eq.) and stirred at room temperature over 20 h. A color change from red to dark green was observed. The solution was filtered through celite to remove the solids and added to DMAP (7.4 mg, 0.061 mmol, 2.0 eq.) and a color change to dark red was observed. The solution was filtered through celite to remove the solids and volatiles were then removed under vacuum. Crystals of the product [(pyrNdipp)$_2$V(DMAP)$_2$] (**3^{DMAP}**) were grown as dark red blocks from concentrated diethyl ether solution of **3^{DMAP}**, using toluene as sorbent (–30 °C, 1 day). The crystalline product was washed with cold pentane and dried in vacuo (Yield: 15 mg, 0.019 mmol, 62.2%, based on **1**). **Elemental analysis**: [(pyrNdipp)$_2$V(DMAP)$_2$] Calculated – $C_{48}H_{62}N_8V$ (802.02 g/mol). C, 71.88%; H, 7.79%; N, 13.97%; Found – C, 71.19%; H, 7.71%; N, 13.81%. **^{1}H NMR**: (400 MHz, C_6D_6) δ (ppm): 9.80 (FWHM = 220 Hz), 4.25 (FWHM = 720 Hz), 2.22 (FWHM = 1100 Hz). **IR:** (KBr) ν (cm^{-1}) 1607 (ν_{imine}).

*Comment: Samples of **3^{DMAP}** under-analyze for carbon, but the relative CHN ratio is satisfactory. For clarity, we calculate the effect of inadvertent oxygenation, manipulative losses, or contamination with a non-CHN material such as glass (1%). For arithmetic reasons, the C-content (>70%) is most sensitive to such effects.*

C: 71.88% × 0.99 = 71.16%, H: 7.79% × 0.99 = 7.71%, N: 13.97% × 0.99 = 13.83%

Analysis of crude reaction mixture. When analyzing a crude reaction mixture by ^{31}P NMR spectroscopy (–800 to +3200 ppm), we did not observe any resonances from P_4. When filtering a crude reaction mixture through glass microfiber filter, washing with THF, and individually analyzing the insoluble particles in the micro-Raman setup, we did not observe red or black phosphorus.

2.4 Synthesis of [(pyrNdipp)$_2$V(THF)$_2$] ($\mathbf{3^{THF}}$)

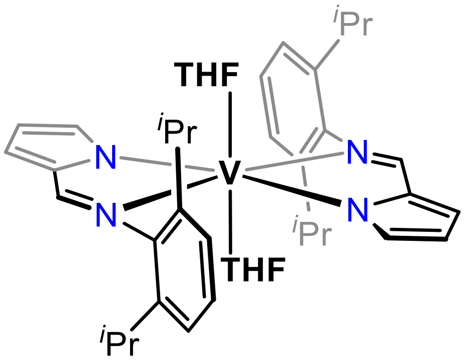

Crystals of [(pyrNdipp)$_2$V(PCO)]·Et_2O (**2**) (10.1 mg, 0.015 mmol, 1.0 eq.) were dissolved in 1 mL THF, and the solution was stirred at room temperature for 10 minutes. The solution was filtered to remove solids and the solvent was removed under vacuum, and dark red block crystals of product ($\mathbf{3^{THF}}$) suitable for single crystal X-ray diffraction were obtained from saturated diethyl ether solution using toluene as sorbent at –30 °C. Further characterization was hampered by the lability of the THF ligand and consequent instability of $\mathbf{3^{THF}}$.

Analysis of crude reaction mixture. When analyzing a crude reaction mixture by ^{31}P NMR spectroscopy (–800 to +3200 ppm), we did not observe any resonances from P_4. When filtering a crude reaction mixture through glass microfiber filter, washing with THF, and individually analyzing the insoluble particles in the micro-Raman setup, we did not observe red or black phosphorus.

2.5 Synthesis of [(pyrNdipp)$_2$V=P=V(pyrNdipp)$_2$] (4)

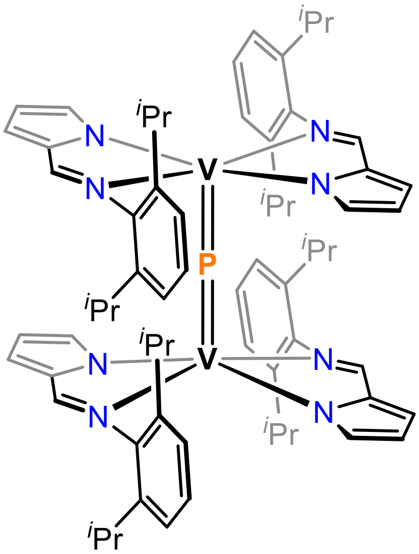

Method 1: Crystals of [(pyrNdipp)$_2$V(PCO)]·Et$_2$O (8.5 mg, 0.012 mmol) were dissolved in 1 mL C_6D_6 in a J Young NMR tube and irradiated with high intensity blue light (450 nm, EcoluChem, 30 W) for 5 h at room temperature. The irradiation time is critical for the reaction to go to completeness, but too long irradiation time led to the formation of [(pyrNdipp)$_3$V] (**6**). A color change from light to darker red was observed. The solvent was removed under vacuum, and the product [(pyrNdipp)$_2$V=P=V(pyrNdipp)$_2$]·Et$_2$O (**4**) was crystallized by concentrating a diethyl ether solution using toluene as sorbent at –30 °C. Dark red block crystals suitable for X-ray diffraction were obtained by this method and to obtain characterization data by elemental analysis, ^{1}H NMR, and IR spectroscopy. (Yield: 2 mg, 0.0016 mmol, 27% based on the vanadium content in **2**).

Method 2: [(pyrNdipp)$_2$VCl(THF)] (**1**) (100 mg, 0.150 mmol. 1.0 eq.) was added to a glass vial followed by Na(OCP) · 2.5 dioxane (46 mg, 0.152 mmol, 1.0 eq.) in 5 mL diethyl ether at room temperature and stirred over 2 h. The solution was filtered over celite to remove NaCl and washed through with additional diethyl ether (3 mL) until the washings were colorless. Diethyl ether was removed under vacuum followed by the dissolution in 10 mL benzene and irradiation with high intensity blue light (450 nm, EcoluChem, 30 W) for 5 h at room temperature. A color change from light to darker red was observed. The solvent was removed under vacuum, and the product [(pyrNdipp)$_2$V=P=V(pyrNdipp)$_2$]·Et$_2$O (**4**) was crystallized by concentrating a diethyl ether solution using toluene as sorbent at –30 °C. Dark red block crystals of **4** were obtained by this method. (Yield: 13 mg, 0.011 mmol, 7% based on the vanadium content in **2**)

Elemental Analysis – [(pyrNdipp)$_2$V=P=V(pyrNdipp)$_2$]·Et$_2$O Calculated – $C_{72}H_{94}N_8OPV_2$ (1220.46 g/mol). C, 70.86%; H, 7.76%; N, 9.18%; P, 2.54%; Found – C, 69.38%; H, 7.59%; N, 8.87%; P, 2.47%. **^{1}H NMR** (400 MHz, C_6D_6) δ (ppm): 8.63 (FWHM = 15.7 Hz), 6.42 (FWHM = 19.5 Hz), 5.94 (FWHM = 16.4 Hz), 1.61 (FWHM = 14.1 Hz), 1.28 (FWHM = 12.9 Hz), 0.95 (FWHM = 11.5 Hz), 0.64 (FWHM = 27.8 Hz), 0.28 (FWHM = 4.5 Hz), -0.14 (FWHM = 29.5 Hz), -3.74 (FWHM = 17.3 Hz). **UV-Vis:** Toluene, λ_{max} [nm, ε (max/sh, M^{-1} cm^{-1})] 297 (max, 37000), 387 (sh, 13000), 478 (max, 8700), 618 (sh, 1800), 866 (max, 1700). **IR:** (KBr) ν (cm^{-1}) 1573 (ν_{imine}).

*Comment: Samples of **4** under-analyze for carbon, but the relative CHNP ratio is satisfactory. For clarity, we calculate the effect of inadvertent oxygenation, manipulative losses, or contamination with a non-CHNP material such as glass (2%). For arithmetic reasons, the C-content (>70%) is most sensitive to such effects.*

C: 70.86% × 0.98 = 69.44%, H: 7.76% × 0.98 = 7.60%, N: 9.18% × 0.98 = 9.00%, P: 2.54% × 0.98 = 2.49%

Analysis of crude reaction mixture. When analyzing a crude reaction mixture by ^{31}P NMR spectroscopy (–800 to +3200 ppm), we did not observe any resonances from P_4. When filtering a crude reaction mixture through glass microfiber filter, washing with toluene, and individually analyzing the insoluble particles in the micro-Raman setup, we did not observe red or black phosphorus.

Cryogenic photolysis studies. In a J Young NMR tube, complex **2** (7.0 mg, 11 µmol) was dissolved in 0.5 ml toluene-d_8, transferred to the NMR spectrometer, cooled to –80 °C, and analyzed by ^{1}H NMR. The sample was then transferred to a glass Dewar cooled using acetone/dry ice, and subjected to photolysis with high-intensity blue light (450 nm, EcoluChem, 30 W) for 1 h. Subsequent ^{1}H NMR analysis of the reaction mixture showed growth of resonances from **4**, decrease of resonances from **2**, and no new ^{31}P NMR resonances (–800 to +3200 ppm, 1024 scans, spectral widths of 800 ppm).

2.6 Synthesis of [(pyrNdipp)$_3$V] (5)

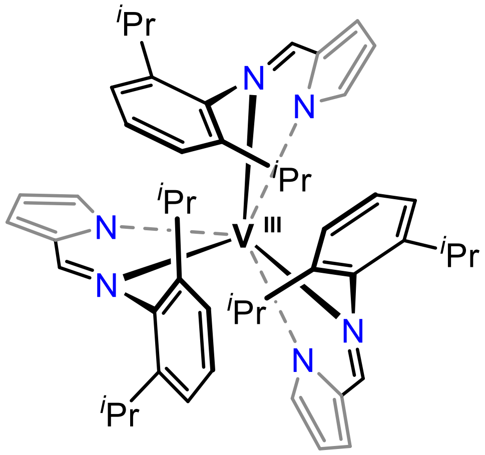

Method 1: Na(pyrNdipp) (8.4 mg, 0.031 mmol, 1.0 eq.) was added to [(pyrNdipp)$_2$VCl(THF)] (**1**) (20 mg, 0.030 mmol. 1.0 eq.) dissolved in 3 mL THF and stirred at room temperature over 20 h. A color change from red to dark red was observed. The solution was filtered through celite to remove the NaCl formed. The solvent was removed under vacuum. The product [(pyrNdipp)$_3$V] (**5**) was crystallized as dark red block crystals by concentrating a diethyl ether solution of **5**, using toluene as sorbent (–30 °C, 2 days). The crystalline product was washed with cold pentane and dried in vacuo (Yield: 12 mg, 0.015 mmol, 49.2%, based on **1**). Single crystals suitable for X-ray diffraction were grown by the same procedure.

Method 2: [(pyrNdipp)$_2$V(PCO)]·Et$_2$O (**2**) (100 mg, 0.145 mmol.) was dissolved in 8 mL toluene and stirred at 110 °C over 20 h. A color change from red to dark red was observed. The solution was filtered through celite to remove the solids. The solvent was removed under vacuum. The product [(pyrNdipp)$_3$V] (**5**) was crystallized as dark red block crystals by concentrating a diethyl ether solution of **5**, using toluene as sorbent (–30 °C, 2 days). The crystalline product was washed with cold pentane and dried in vacuo (Yield: 47 mg, 0.058 mmol, 40.0%, based on the vanadium content in **2**). Single crystals suitable for X-ray diffraction were grown by the same procedure.

Elemental Analysis – [(pyrNdipp)$_3$V] Calculated – $C_{51}H_{63}N_6V$ (811.05 g/mol). C, 75.53%; H, 7.83%; N, 10.36%. Found – C, 74.88%; H, 7.77%; N, 10.11%. **^{1}H NMR**: ^{1}H NMR (400 MHz, C_6D_6) δ 8.91 (FWHM = 32.8 Hz), 5.92 (FWHM = 37.8 Hz), 4.11 (FWHM = 21.8 Hz), 3.64 (FWHM = 115.8 Hz), 1.49 (FWHM = 82.5 Hz), 1.02 (FWHM = 28.6 Hz), 0.63 (FWHM = 34.6 Hz), -2.44 (FWHM = 76.5 Hz). **Magnetic moment**, μ_{eff} (Evans' method, THF-d_8, 298 K): 2.79 μ_B. **IR:** (KBr) ν (cm^{-1}) 1575 (ν_{imine}).

3. NMR Spectroscopy

3.1 ^{1}H NMR spectrum of [(pyrNdipp)$_2$VCl(THF)] (1)

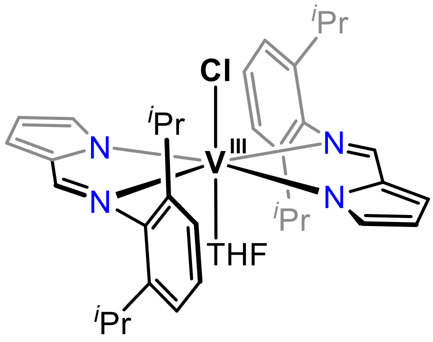

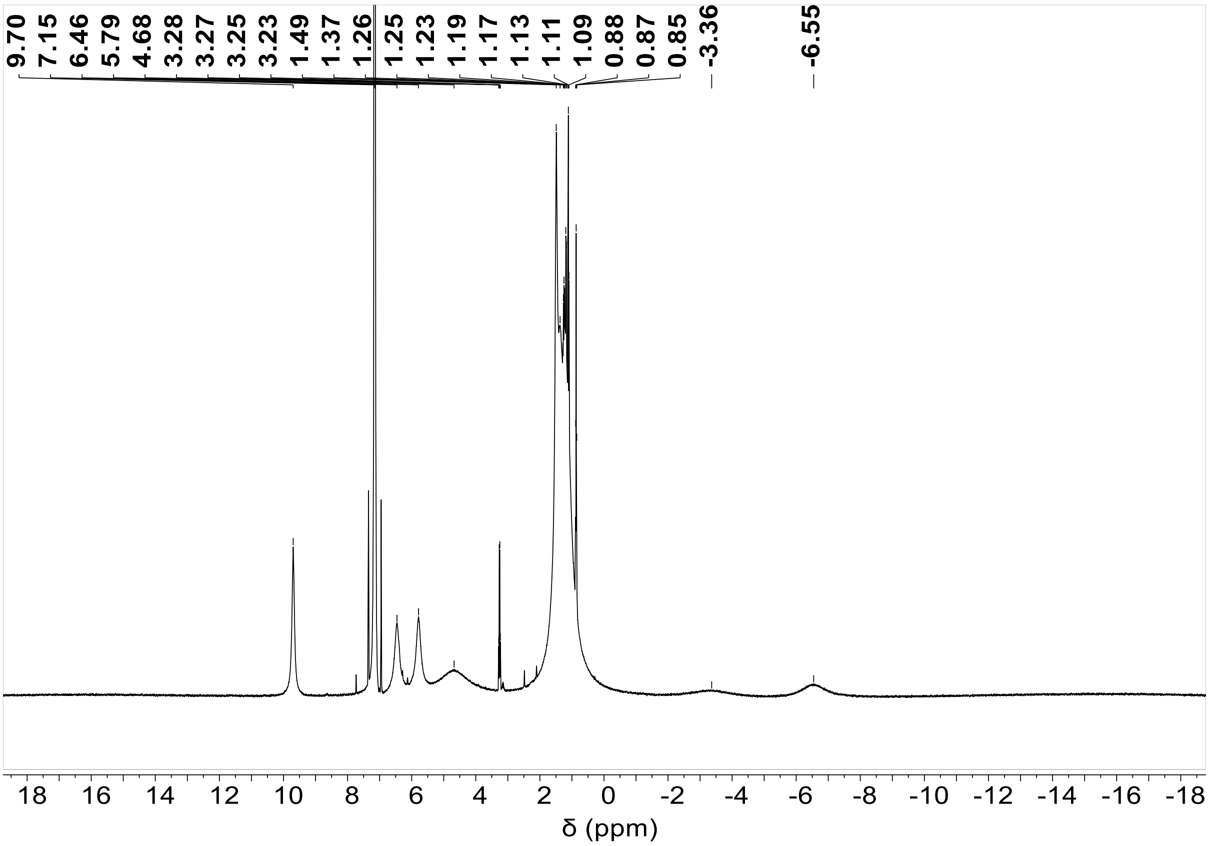

*Figure S1. ^{1}H NMR spectrum (400 MHz) of complex [(pyrNdipp)$_2$VCl(THF)] (**1**) in C_6D_6 (18.8 mM) at 298 K. Residual solvent signals at 0.87 (t), 1.23 (m) (n-Pentane); 1.11 (t), 3.26 (q) (Et_2O); 7.15 ppm (C_6D_6).*

3.2 ^{1}H NMR spectrum of [(pyrNdipp)$_2$V(PCO)] (2)

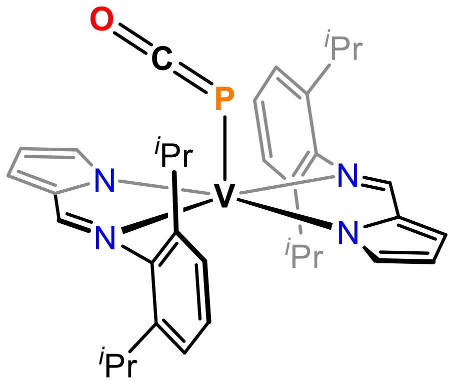

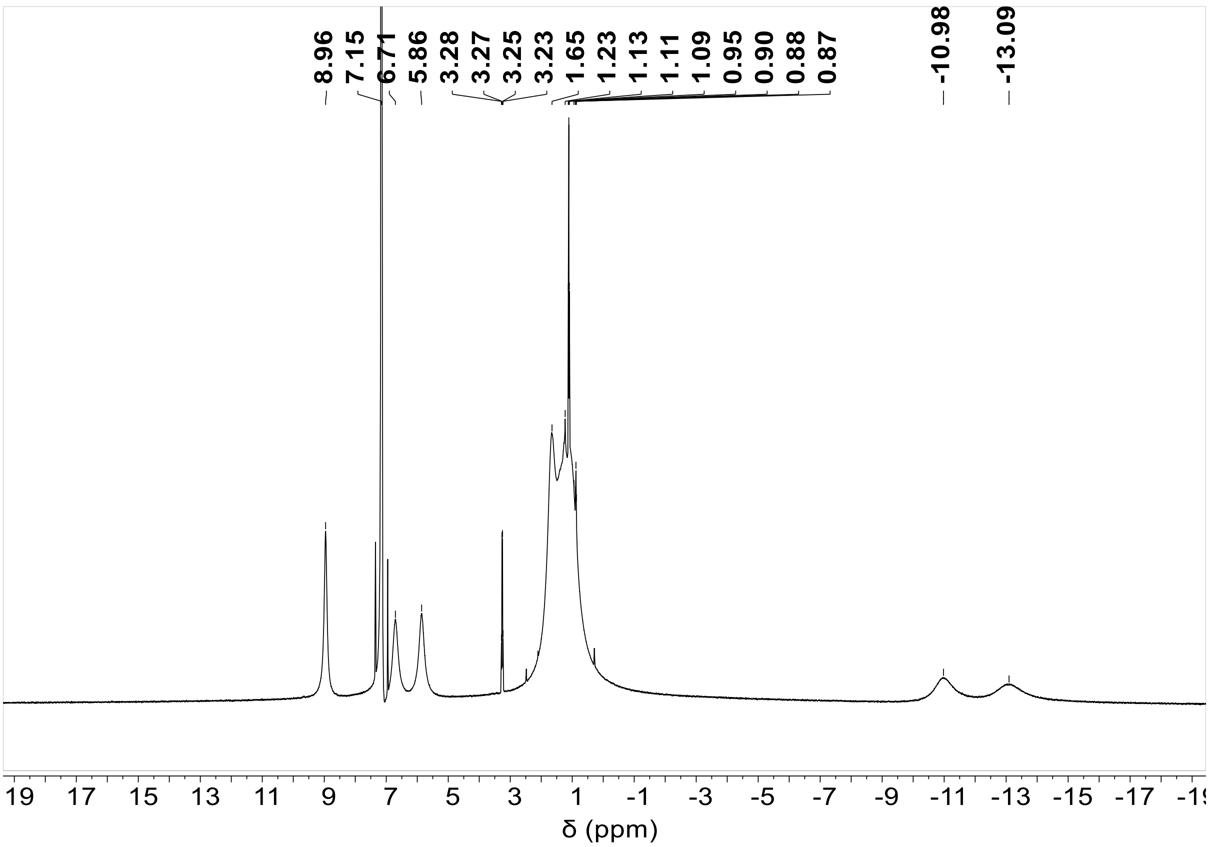

*Figure S2. ^{1}H NMR spectrum (400 MHz) of [(pyrNdipp)$_2$V(PCO)] (**2**) in C_6D_6 (18.1 mM) at 298 K. Residual solvent signals at 0.87 (t), 1.23 (m) (n-Pentane); 1.11 (t), 3.26 (q) (Et_2O); 7.15 ppm (C_6D_6).*

3.3 ^{1}H NMR spectrum of [(pyrNdipp)$_2$V(DMAP)$_2$] (3^{DMAP})

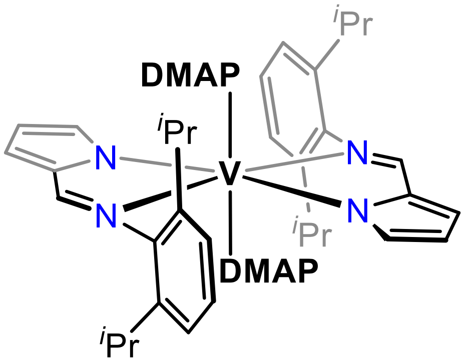

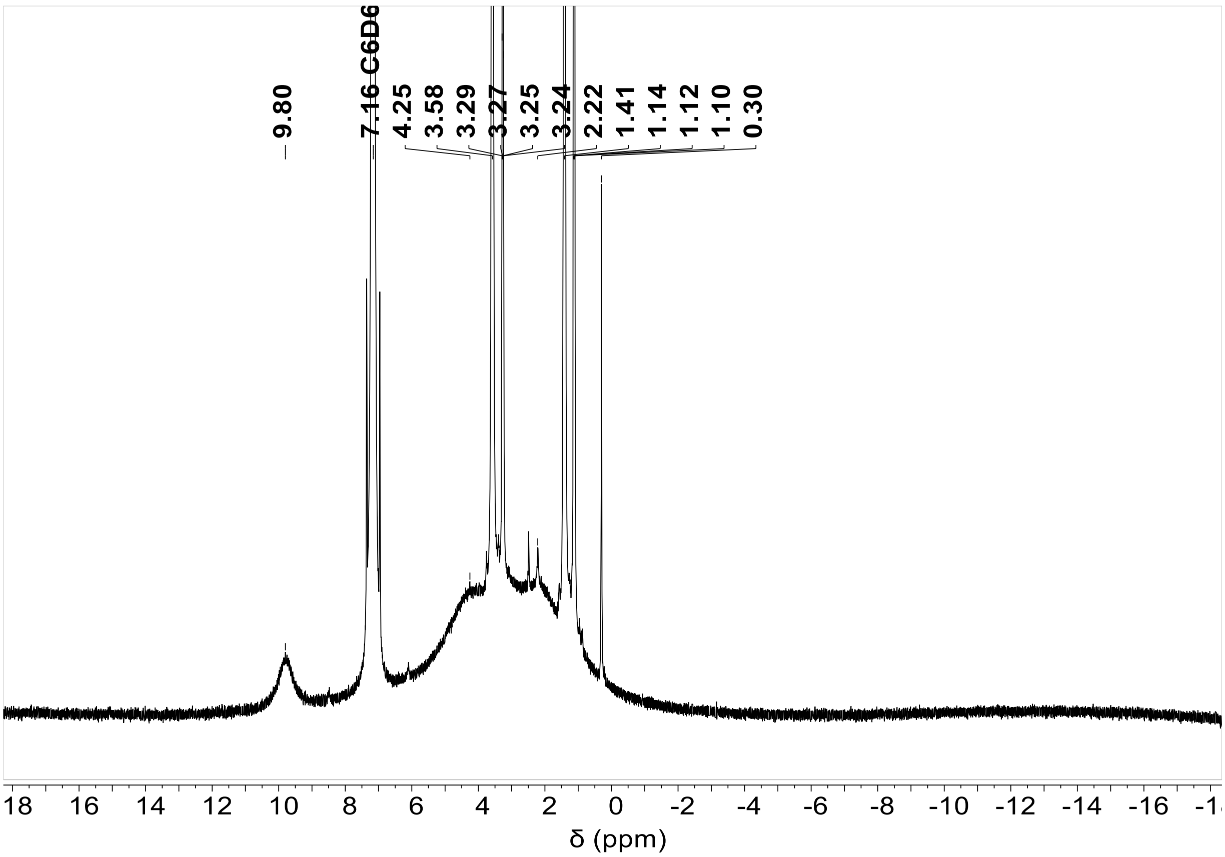

*Figure S3. ^{1}H NMR spectrum (400 MHz) of [(pyrNdipp)$_2$V(DMAP)$_2$] (**3^{DMAP}**) in C_6D_6 (12.5 mM) at 298 K. Residual solvent signals at 1.41 (m), 3.58 (m) (THF); 1.11 (t), 3.26 (q) (Et_2O); 7.16 ppm (C_6D_6); 0.30 ppm (s) (silicone grease).*

3.4 ^{1}H NMR spectrum of [(pyrNdipp)$_2$V=P=V(pyrNdipp)$_2$] (4)

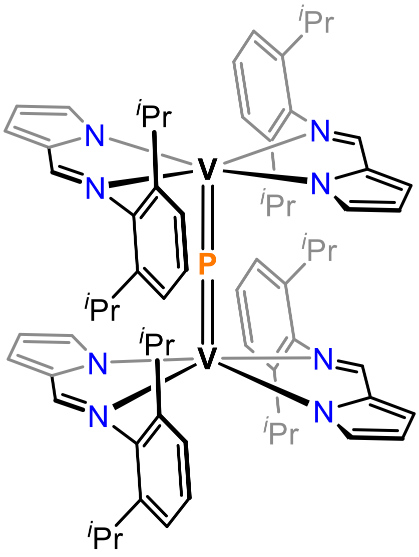

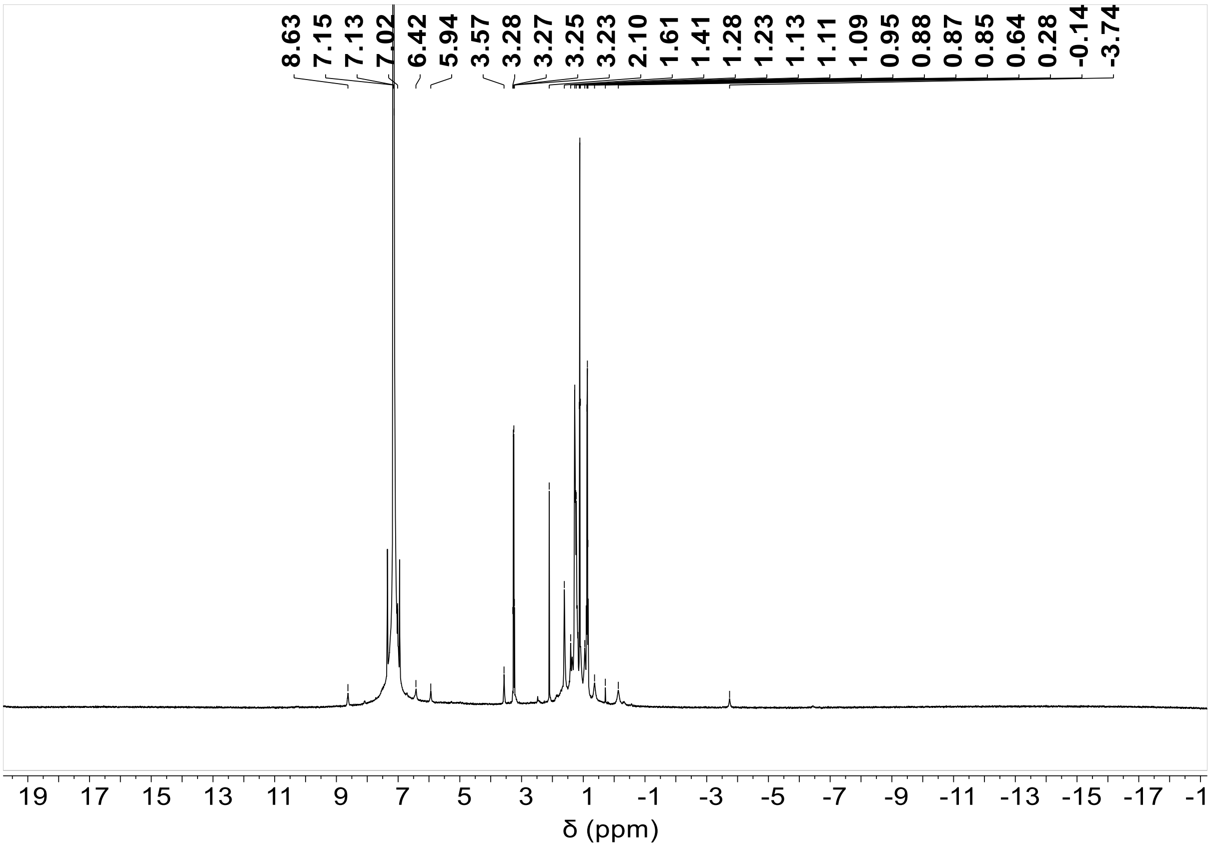

*Figure S4. ^{1}H NMR spectrum (400 MHz) of [(pyrNdipp)$_2$V=P=V(pyrNdipp)$_2$] (**4**) in C_6D_6 (4.7 mM) at 298 K. Residual solvent signals at 0.87 (t), 1.23 (m) (n-Pentane); 1.41 (m), 3.57 (m) (THF); 1.11 (t), 3.26 (q) (Et_2O); 7.13 (m), 7.02 (m), 2.10 (s) (toluene); 7.15 ppm (C_6D_6).*

3.5 ^{1}H NMR spectrum of [(pyrNdipp)$_3$V] (5)

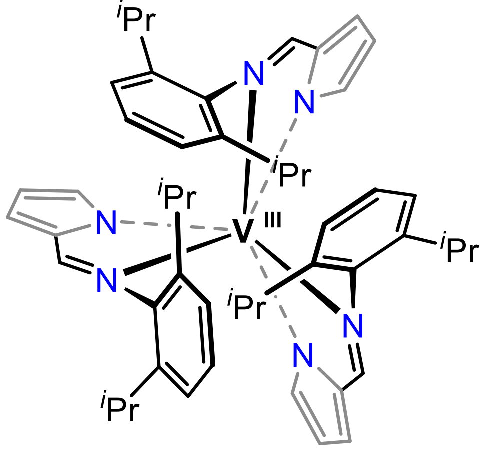

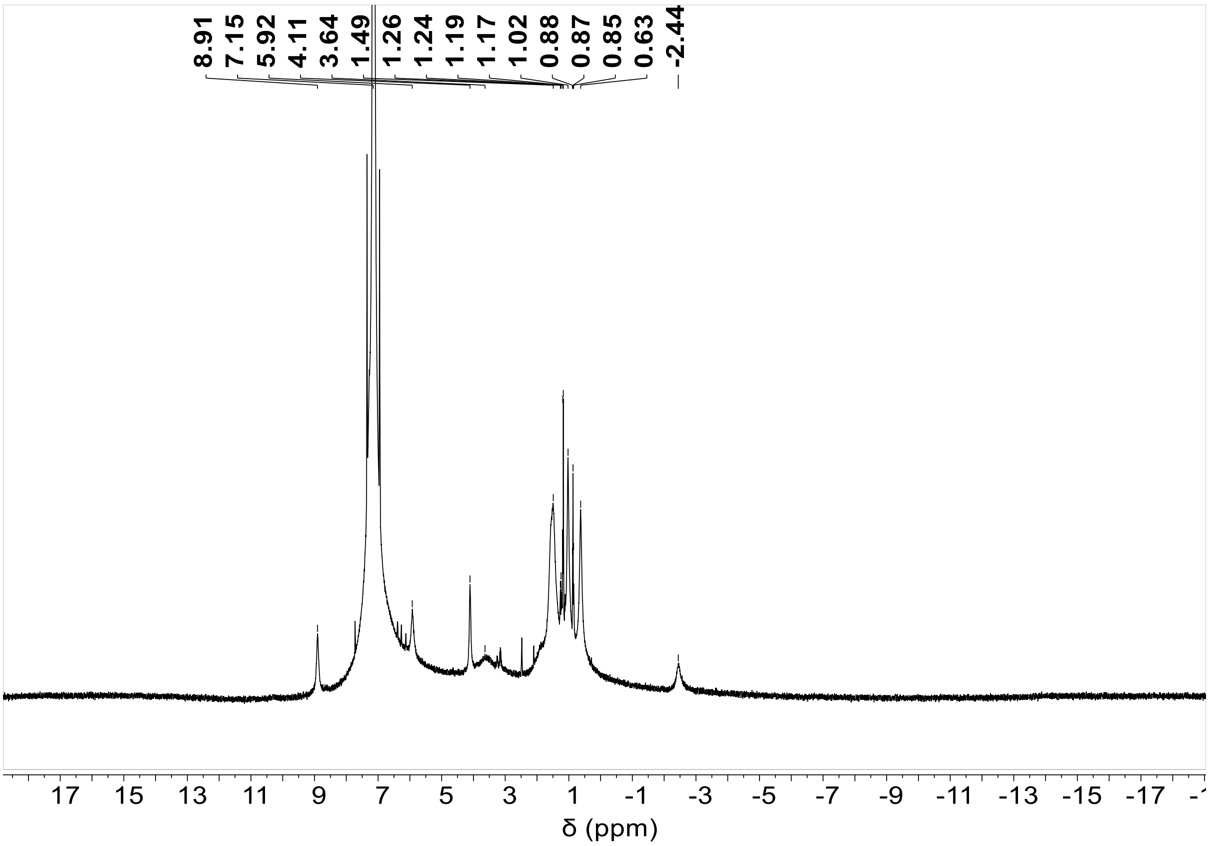

*Figure S5. ^{1}H NMR spectrum (400 MHz) of [(pyrNdipp)$_3$V] (**5**) in C_6D_6 (10.6 mM) at 298 K. Residual solvent signals at 0.87 (t), 1.23 (m) (n-Pentane); 7.15 ppm (C_6D_6).*

4. IR Spectroscopy

4.1 IR Spectrum of [(pyrNdipp)$_2$VCl(THF)] (1)

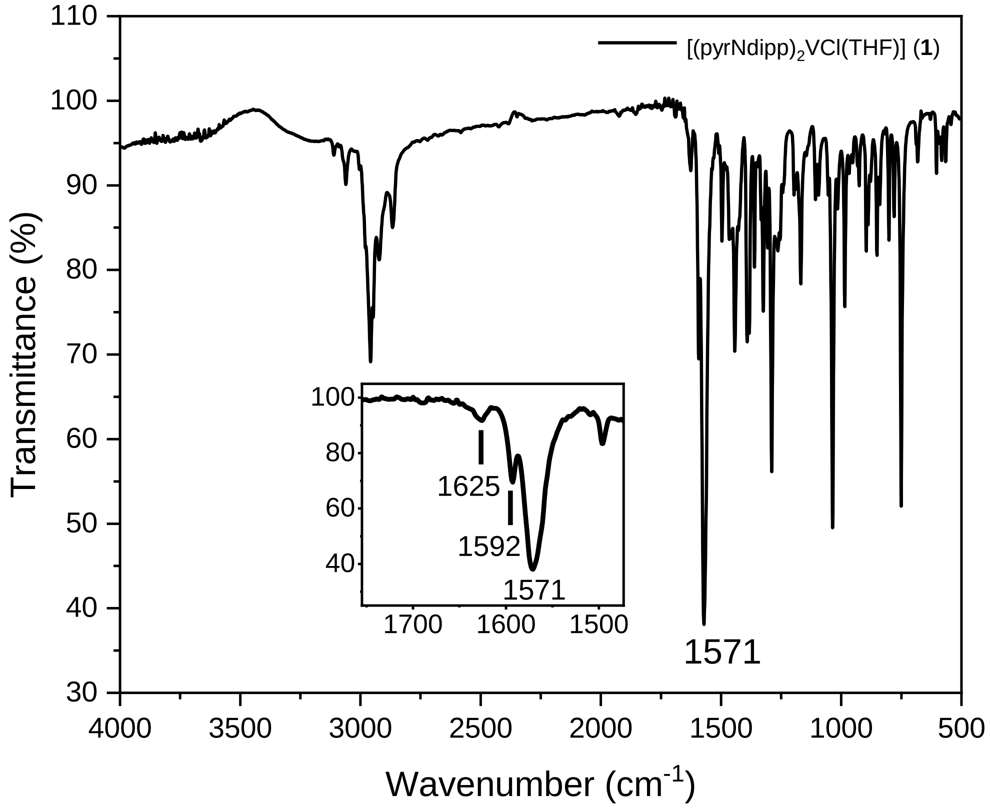

*Figure S6. IR spectrum of solid [(pyrNdipp)$_2$VCl(THF)] (**1**) in KBr pellet. The inset highlights minor peaks from the imine group, where the peak at 1625 cm^{-1} matches the literature value.*[4]

4.2 IR Spectrum of [(pyrNdipp)$_2$V(PCO)] (2)

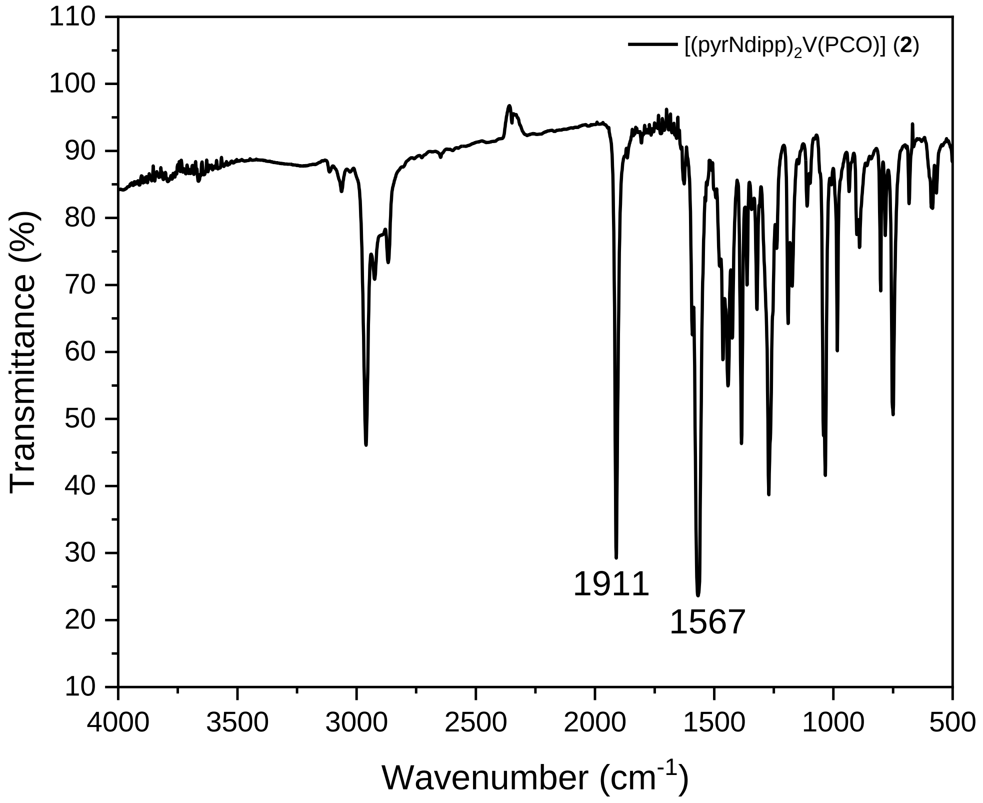

*Figure S7. IR spectrum of solid [(pyrNdipp)$_2$V(PCO)] (**2**) in KBr pellet.*

4.3 IR Spectrum of [(pyrNdipp)$_2$V(DMAP)$_2$] (3^{DMAP})

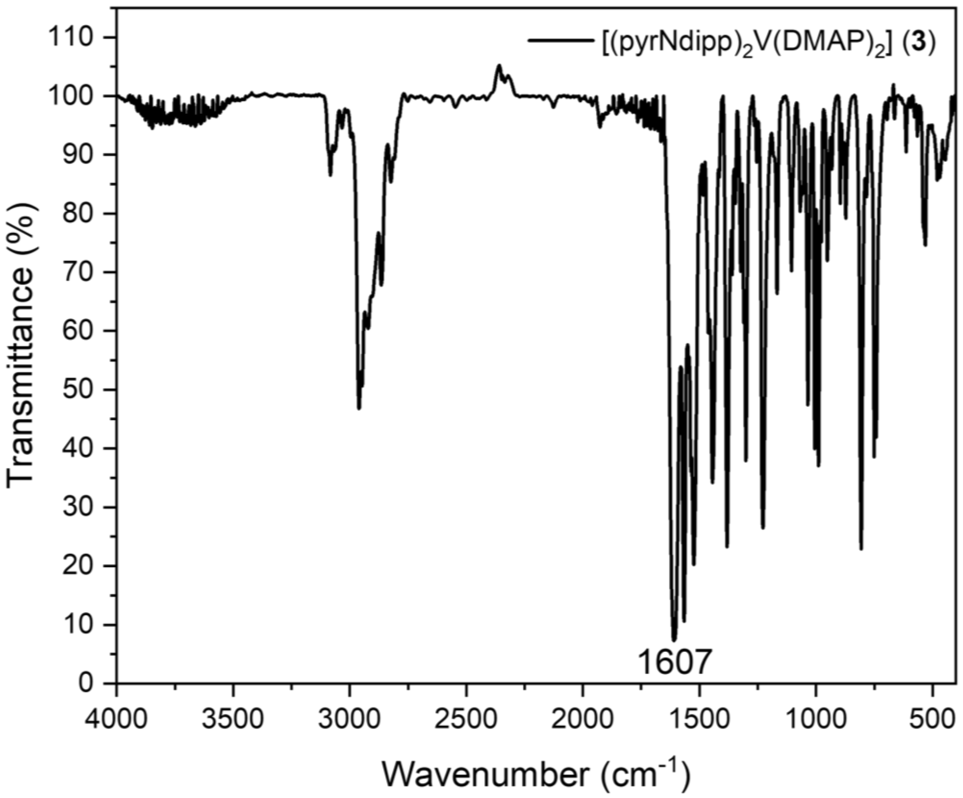

*Figure S8. IR spectrum of solid [(pyrNdipp)$_2$V(DMAP)$_2$] (**3**^{DMAP}) in KBr pellet.*

4.4 IR Spectrum of [(pyrNdipp)$_2$V=P=V(pyrNdipp)$_2$] (4)

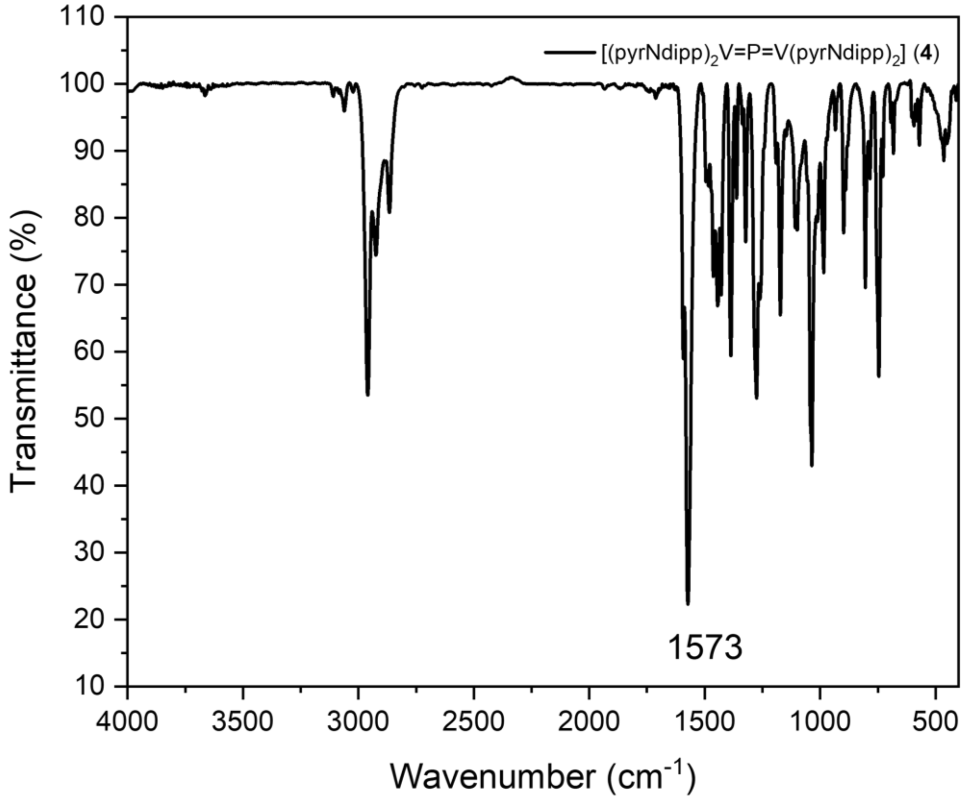

*Figure S9. IR spectrum of solid [(pyrNdipp)$_2$V=P=V(pyrNdipp)$_2$] (**4**) in KBr pellet.*

4.5 IR Spectrum of [(pyrNdipp)$_3$V] (5)

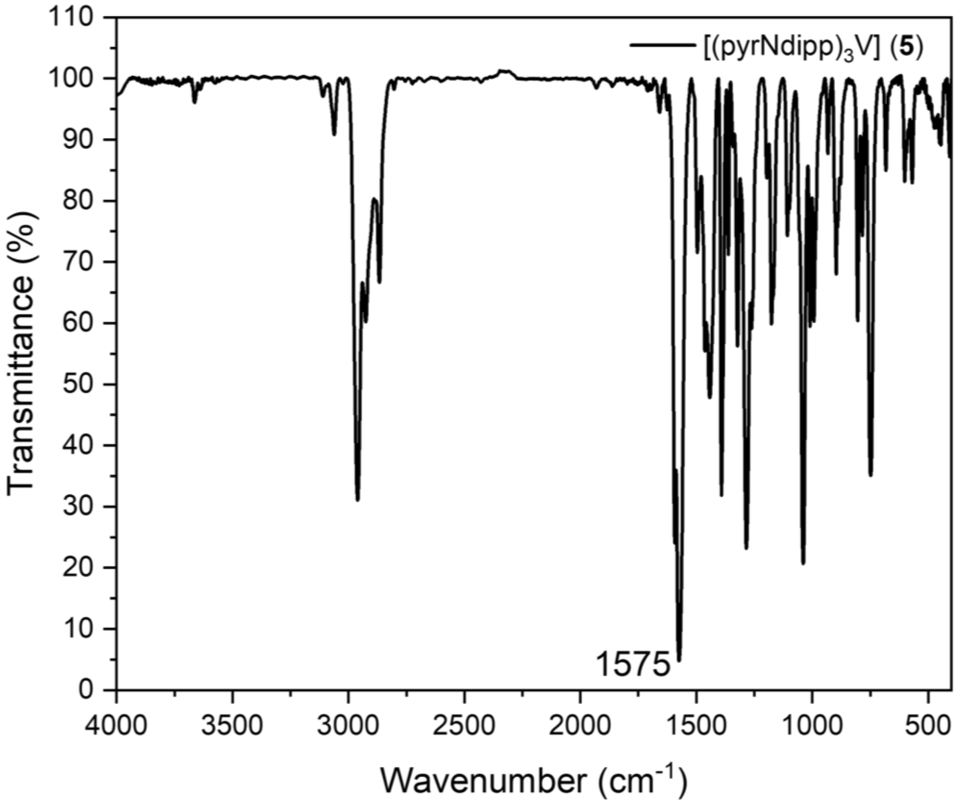

*Figure S10. IR spectrum of solid [(pyrNdipp)$_3$V] (**5**) in KBr pellet.*

5. UV-Vis Spectroscopy

5.1 UV-Vis spectral data for [(pyrNdipp)$_2$VCl(THF)] (1)

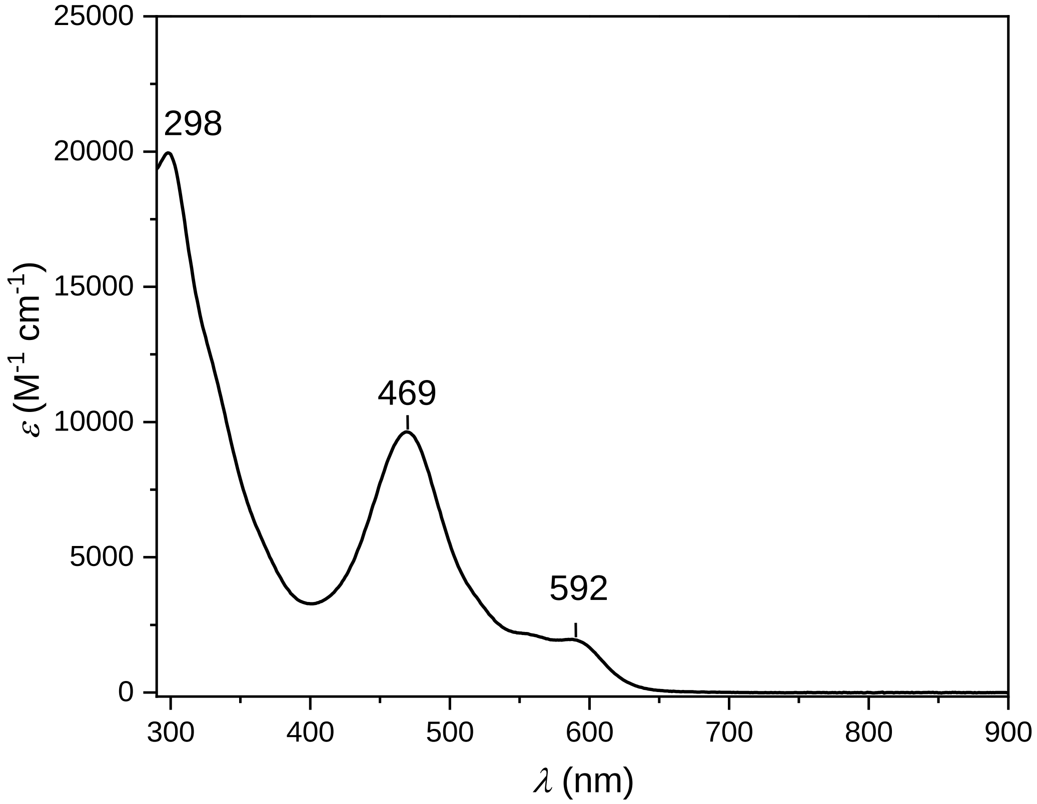

*Figure S11. UV-Vis spectrum of [(pyrNdipp)$_2$VCl(THF)] (**1**) dissolved in toluene recorded at 4.88 × 10^{-5} M.*

5.2 UV-Vis spectral data for [(pyrNdipp)$_2$V(PCO)] (2)

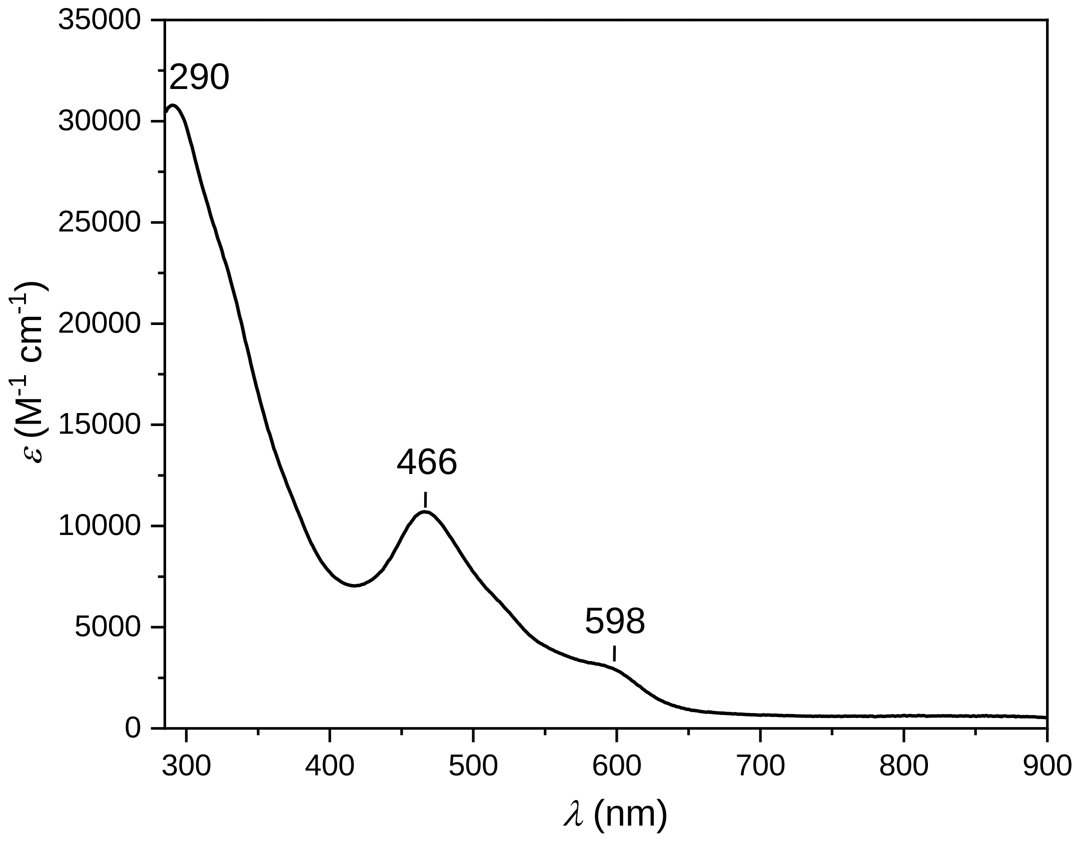

*Figure S12. UV-Vis spectrum of [(pyrNdipp)$_2$V(PCO)] (**2**) dissolved in toluene recorded at 2.85 × 10^{-5} M.*

5.3 UV-Vis spectral data for [(pyrNdipp)$_2$V=P=V(pyrNdipp)$_2$] (4)

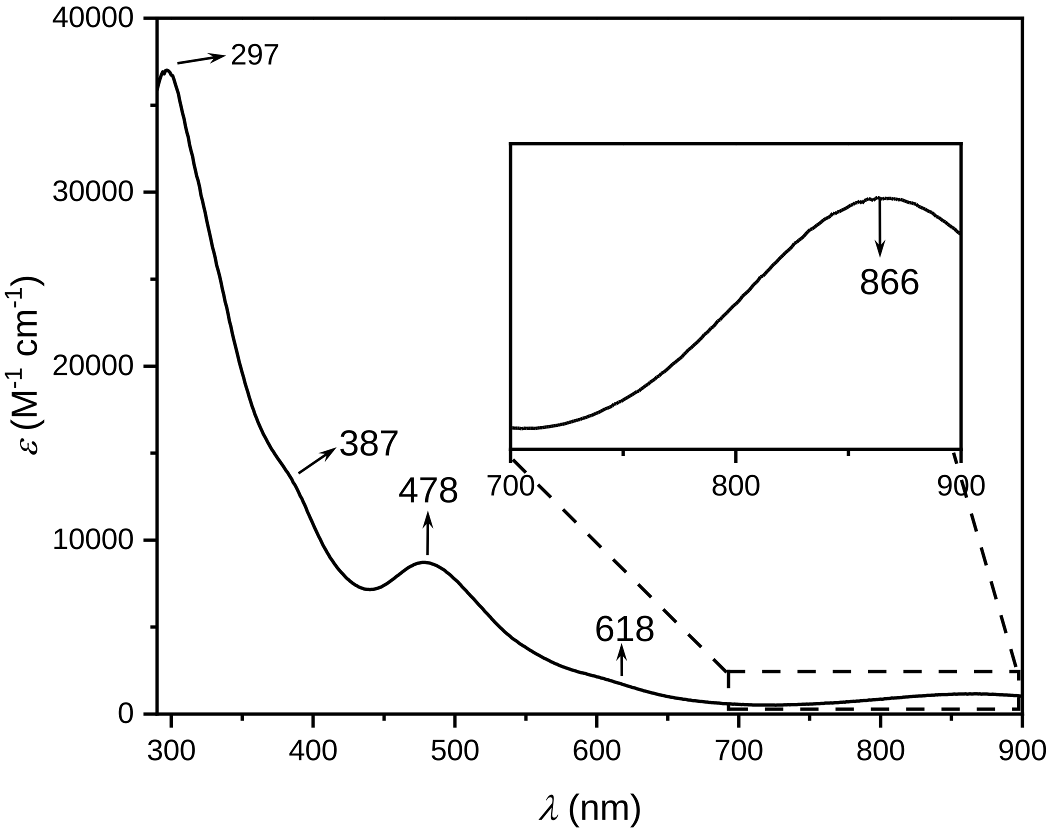

*Figure S13. UV-Vis spectrum of [(pyrNdipp)$_2$V=P=V(pyrNdipp)$_2$] (**4**) dissolved in toluene recorded at 6.14 × 10^{-5} M; inset shows data recorded at 1.94 × 10^{-4} M.*

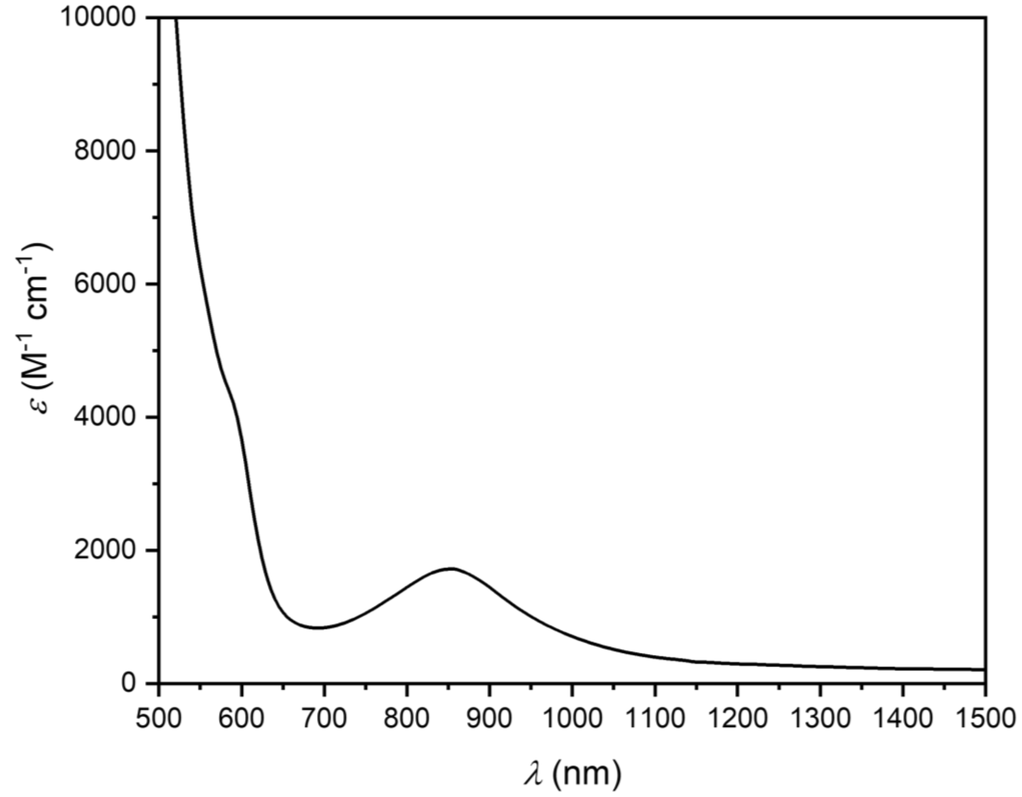

*Figure S14. NIR spectrum of [(pyrNdipp)$_2$V=P=V(pyrNdipp)$_2$] (**4**) dissolved in toluene recorded at 1.40 × 10^{-4} M.*

6. Raman Spectroscopy

6.1 Raman spectral data for [(pyrNdipp)$_2$V(PCO)] (2)

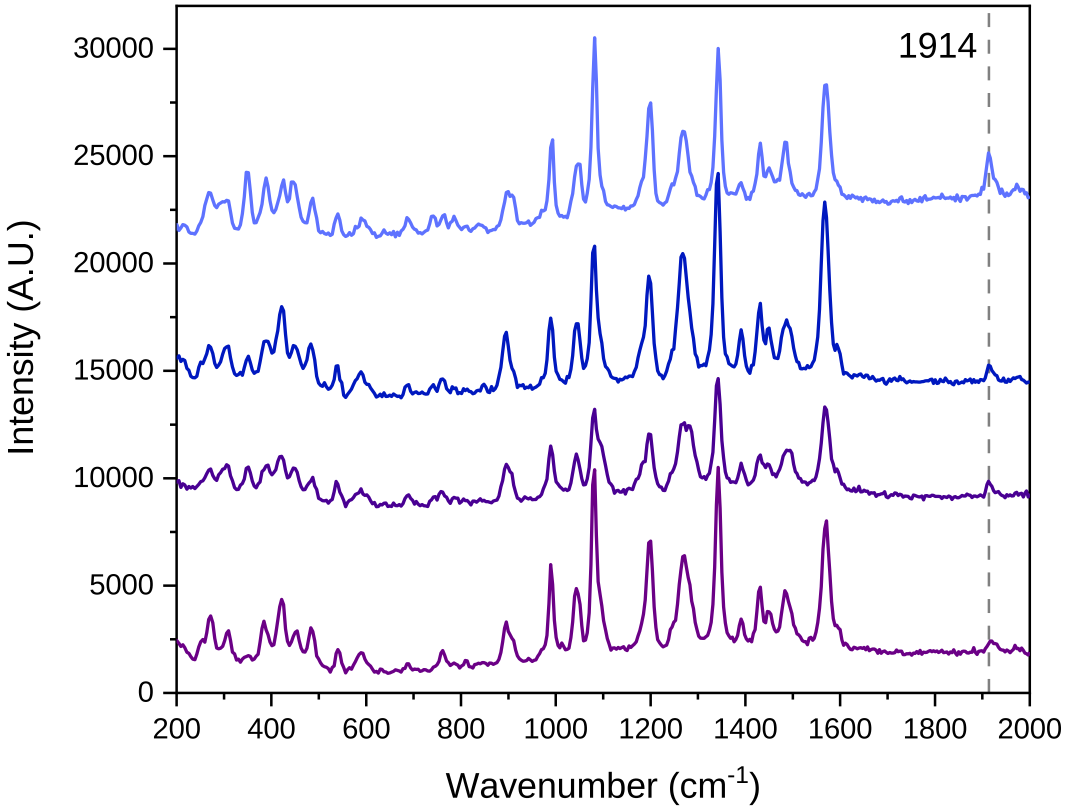

*Figure S15. Raman spectra of four different single crystals of [(pyrNdipp)$_2$V(PCO)] (**2**) recorded using a 488 nm Ar-ion laser.*

6.2 Raman spectral data for [(pyrNdipp)$_2$V=P=V(pyrNdipp)$_2$] (4)

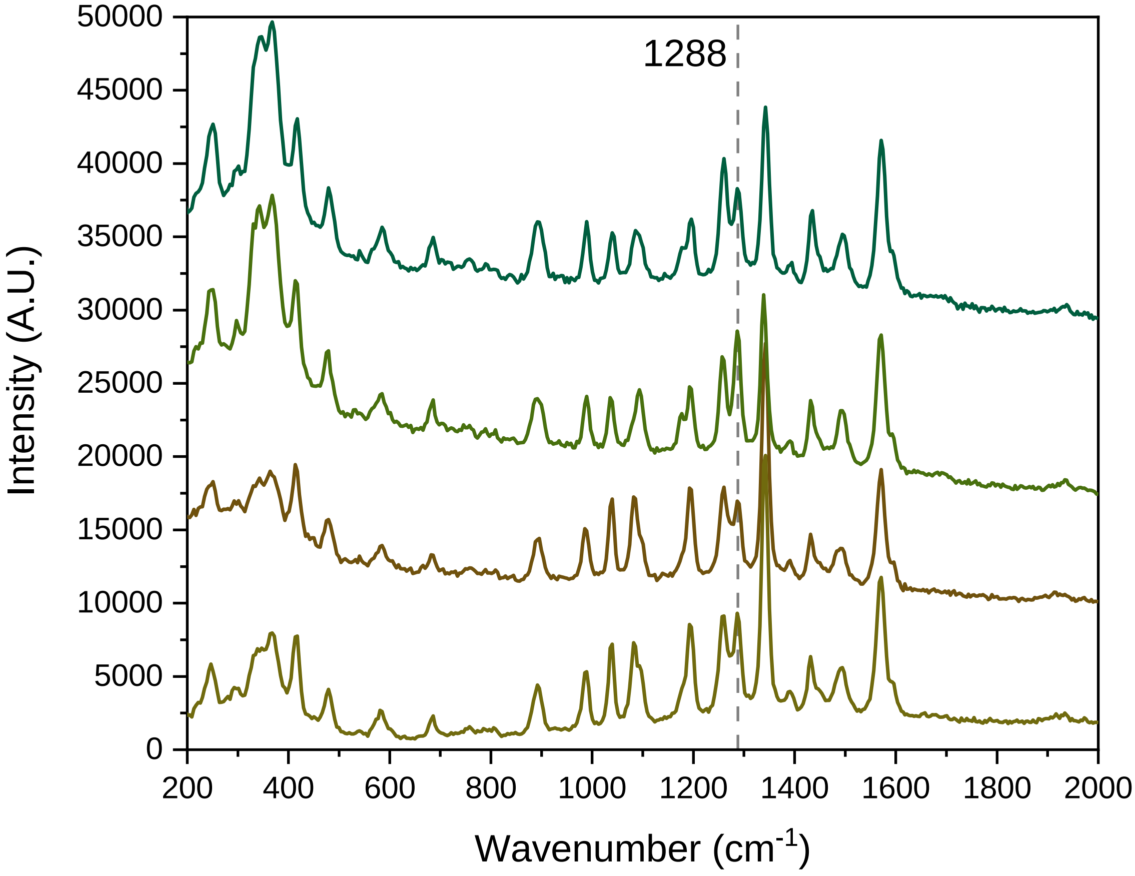

*Figure S16. Raman spectra of four different single crystals of [(pyrNdipp)$_2$V=P=V(pyrNdipp)$_2$] (**4**) recorded using a 488 nm Ar-ion laser.*

6.3 Raman spectra of [(pyrNdipp)$_2$V(PCO)] (2) compared with [(pyrNdipp)$_2$V=P=V(pyrNdipp)$_2$] (4)

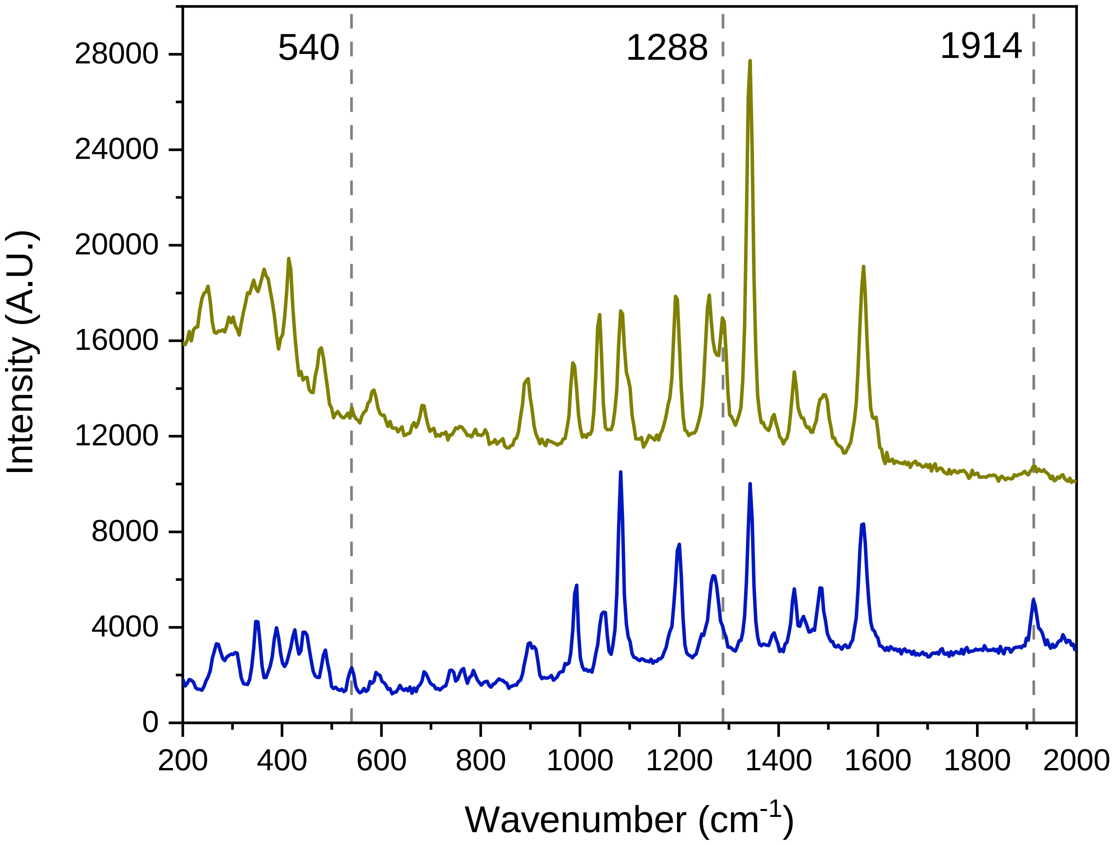

*Figure S17. Raman spectra of single crystals of **2** (bottom) and **4** (top) using a 488 nm Ar-ion laser.*

7. X-ray Absorption Spectroscopy

XANES Spectra of Vanadium Complexes

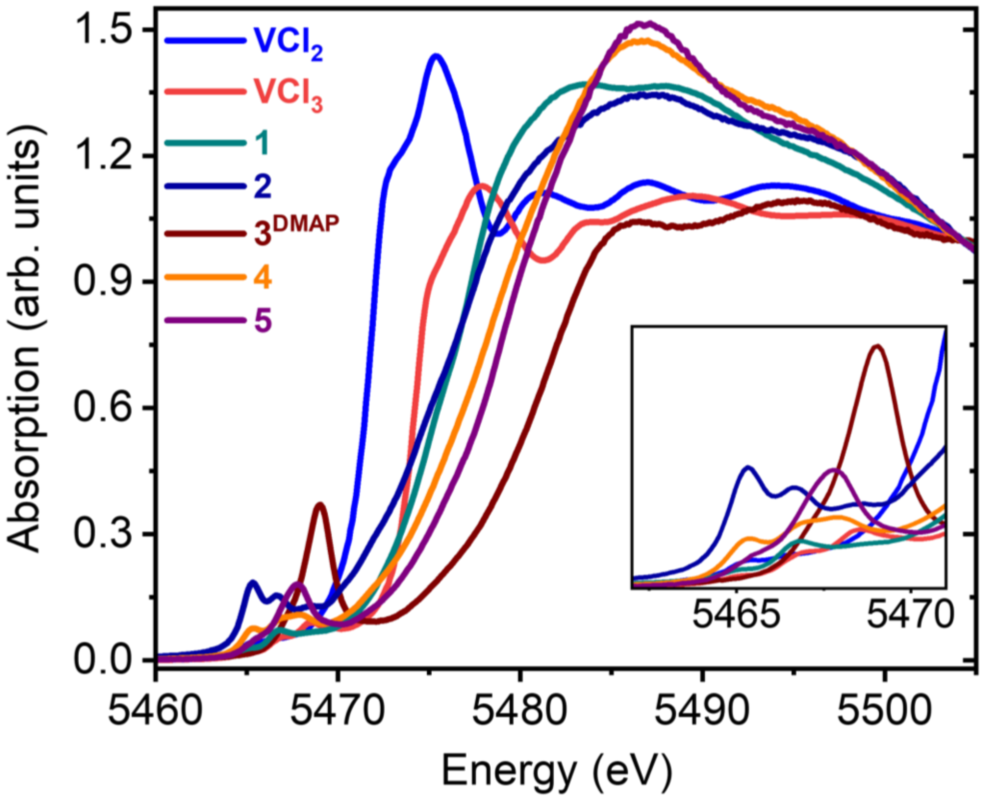

Figure S18. V K-Edge XANES Spectra of ***VCl_2****,* ***VCl_3****,* ***1****,* ***2****,* ***3^{DMAP}****,* ***4****, and* ***5****.*

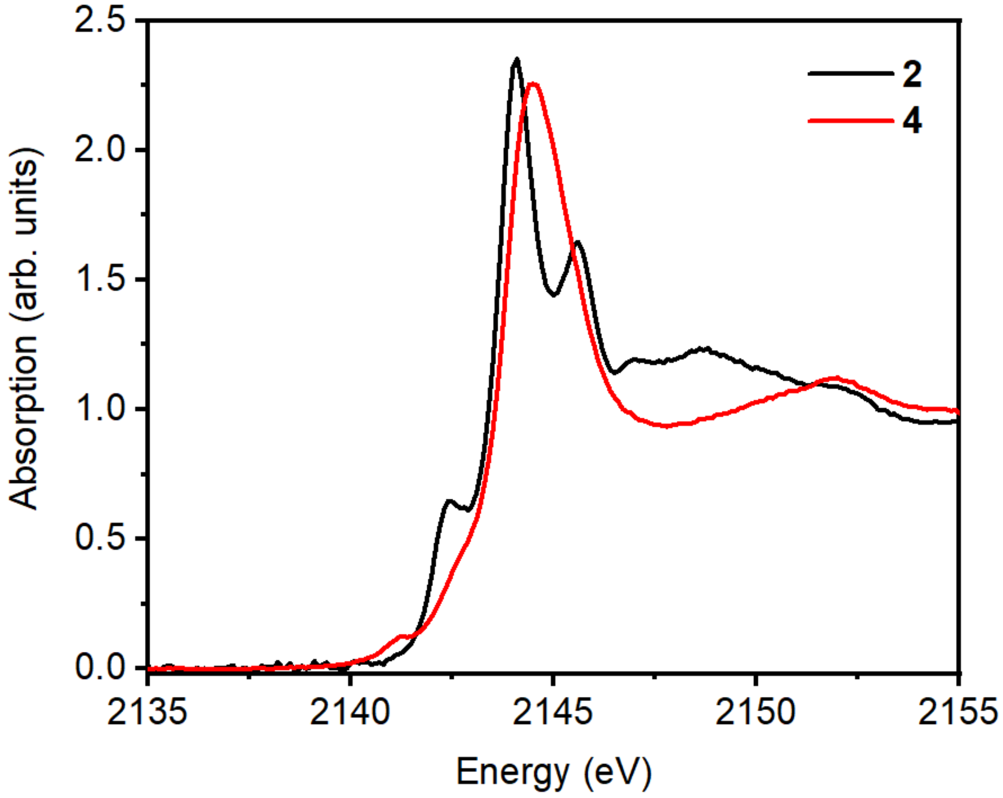

*Figure S19. P K-Edge XANES Spectra of **2**, and **4**.*

8. EPR Spectroscopy

X-band EPR spectra were recorded on a polycrystalline sample of **4** kept in a flame-sealed capillary. The sample showed a magnetically dilute $S = 1/2$ impurity of V^{IV}, with well-resolved hyperfine coupling to ^{51}V (I = 7/2), from the flame-sealing protocol. Complex **4** is practically EPR silent from 250 K to 20 K, in line with its orbital degeneracy. From 14 K to 5 K, a broad resonance began to appear, and transitions between the $m_S = \pm½$ states of an $S_T = 3/2$ species could be simulated using magnetic parameters obtained from independent bulk magnetometric measurements (S = 3/2, D = –2.7 cm^{-1}, g_{eff} = 1.76). Given the concentrated crystalline sample and the large zero-field splitting, observation of resolved ^{51}V or ^{31}P hyperfine coupling is not expected.

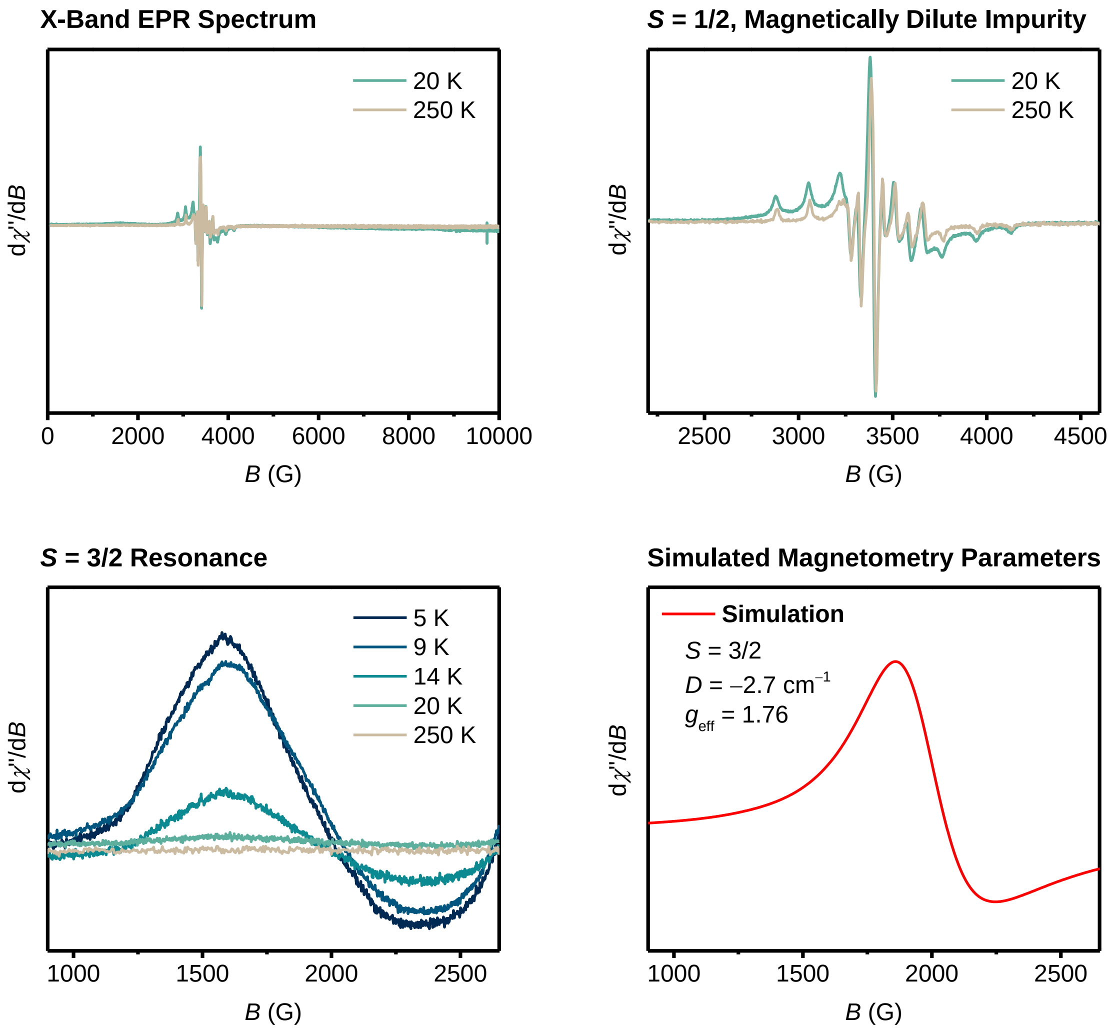

*Figure S20. X-band EPR spectra recorded on a polycrystalline sample of **4**.*

9. Crystallographic Data

9.1 Crystallographic Tables

Table S1. Crystallographic data for complexes **2**, **3**$^{\mathbf{DMAP}}$ and **3**$^{\mathbf{THF}}$.

**Complex**<br>**CCDC entry**	[(pyrNdipp)$_2$V(PCO)] (**2**)<br>**2501031**	[(pyrNdipp)$_2$V(DMAP)$_2$] (**3**$^{\mathbf{DMAP}}$)<br>**2501032**	[(pyrNdipp)$_2$V(THF)$_2$] (**3**$^{\mathbf{THF}}$)<br>**2501033**
Empirical formula	$C_{35}H_{42}N_4OPV$	$C_{48}H_{62}N_8V$	$C_{42}H_{58}N_4O_2V$
Formula weight	616.63	802.023	701.86
Temperature/K	100(2)	100(2)	100(2)
Crystal system	monoclinic	triclinic	triclinic
Space group	*C*2/*c*	*P*-1	*P*-1
a/Å	19.9514(4)	9.7748(5)	9.6063(6)
b/Å	10.1011(2)	11.8735(7)	10.5282(6)
c/Å	16.5568(4)	12.2776(7)	10.7553(5)
α/°	90	116.512(6)	65.475(5)
β/°	98.941(2)	101.748(5)	80.438(4)
γ/°	90	98.147(5)	73.504(5)
Volume/Å^3	3296.16(12)	1203.59(15)	947.44(10)
Z	4	1	1
ρ_{calc} g/cm^3	1.243	1.107	1.230
μ/mm^{-1}	0.382	0.245	0.302
F(000)	1304.0	429.5	377.0
Crystal size/mm^3	0.39 × 0.19 × 0.13	0.74 × 0.51 × 0.17	0.64 × 0.38 × 0.27
Radiation	Mo K_α (λ = 0.71073)	Mo K_α (λ = 0.71073)	Mo K_α (λ = 0.71073)
2Θ range for data collection/°	6.952 to 50.748	6.78 to 50.78	6.938 to 50.698
Index ranges	$-23 \le h \le 24$, $-12 \le k \le 12$, $-19 \le l \le 19$	$-11 \le h \le 11$, $-14 \le k \le 14$, $-14 \le l \le 14$	$-11 \le h \le 11$, $-12 \le k \le 12$, $-12 \le l \le 12$
Reflections collected	35206	26292	12820
Independent reflections	3027 [R_{int} = 0.0590, R_{sigma} = 0.0307]	4409 [R_{int} = 0.0633, R_{sigma} = 0.0511]	3468 [R_{int} = 0.0336, R_{sigma} = 0.0385]
Data/restraints/parameters	3027/0/208	4409/0/265	3468/0/227
Goodness-of-fit on F^2	1.042	0.930	0.990
Final R indexes [$I \ge 2\sigma(I)$]	R_1 = 0.0367, wR_2 = 0.0798	R_1 = 0.0456, wR_2 = 0.1251	R_1 = 0.0422, wR_2 = 0.1254
Final R indexes [all data]	R_1 = 0.0520, wR_2 = 0.0864	R_1 = 0.0644, wR_2 = 0.1398	R_1 = 0.0548, wR_2 = 0.1382
Largest diff. peak/hole / e Å^{-3}	0.30/-0.28	0.53/-0.31	0.51/-0.21

Table S2. Crystallographic data for complexes **4** as a toluene solvate and **5**.

**Complex**<br>**CCDC entry**	[(pyrNdipp)$_2$V=P=V(pyrNdipp)$_2$] (**4**)<br>**2501034**	[(pyrNdipp)$_3$V] (**5**)<br>**2501035**
Empirical formula	$C_{75}H_{92}N_8PV_2$	$C_{51}H_{63}N_6V$
Formula weight	1238.477	811.01
Temperature/K	150(2)	100(2)
Crystal system	monoclinic	monoclinic
Space group	P2$_1$/*c*	*P*2$_1$/*n*
a/Å	21.0107(7)	18.1499(6)
b/Å	20.8598(4)	13.1725(4)
c/Å	17.5835(5)	18.7148(6)
α/°	90	90
β/°	110.663(4)	92.558(3)
γ/°	90	90
Volume/Å^3	7210.7(4)	4469.9(2)
Z	4	4
ρ_{calc} g/cm^3	1.141	1.205
μ/mm^{-1}	0.326	0.263
F(000)	2640.3	1736.0
Crystal size/mm^3	0.37 × 0.234 × 0.082	0.99 × 0.71 × 0.39
Radiation	Mo K_α (λ = 0.71073)	Mo K_α (λ = 0.71073)
2Θ range for data collection/°	6.52 to 50.8	6.902 to 50.7
Index ranges	$-25 \le h \le 25, -25 \le k \le 25, -21 \le l \le 21$	$-21 \le h \le 21, -15 \le k \le 15, -22 \le l \le 22$
Reflections collected	148378	85899
Independent reflections	13213 [R_{int} = 0.1576, R_{sigma} = 0.0947]	8165 [R_{int} = 0.0862, R_{sigma} = 0.0434]
Data/restraints/parameters	13213/0/792	8165/0/535
Goodness-of-fit on F^2	1.002	1.041
Final *R* indexes [$I \ge 2\sigma(I)$]	R_1 = 0.0582, wR_2 = 0.1212	R_1 = 0.0448, wR_2 = 0.1067
Final *R* indexes [all data]	R_1 = 0.1186, wR_2 = 0.1456	R_1 = 0.0660, wR_2 = 0.1193
Largest diff. peak/hole / e Å^{-3}	0.88/-0.66	0.53/-0.41

9.2 X-ray Molecular Structures

9.2.1 [(pyrNdipp)$_2$V(PCO)] (2)

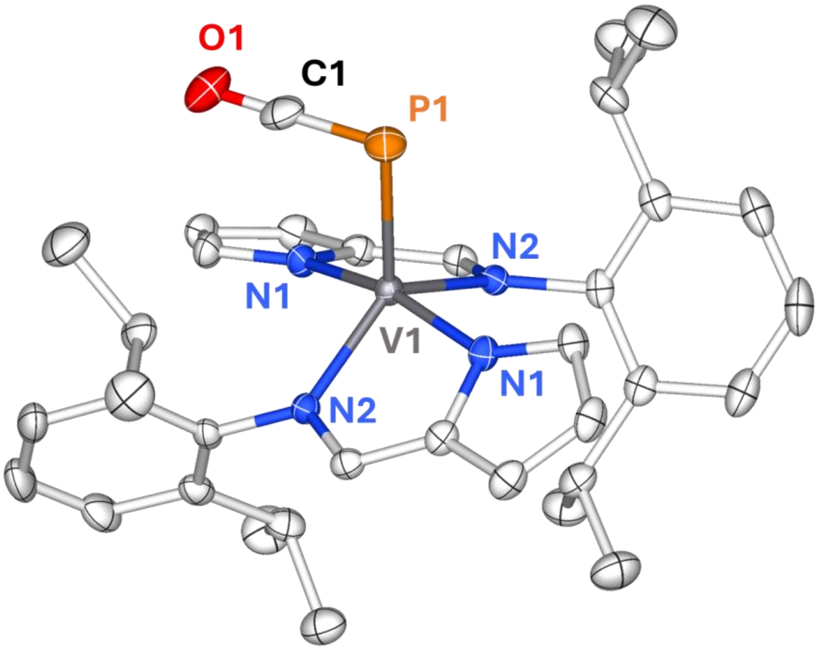

*Figure S21. Molecular structure of [(pyrNdipp)$_2$V(PCO)] (**2**) obtained through single crystal XRD, CCDC entry 2501031. Crystals grown by vapor diffusion from Et_2O and toluene. Hydrogen atoms are omitted for clarity, ellipsoids at 50% probability level. Orientational disorder of the PCO^{-} ligand is not shown. Color code: Light gray (C), blue (N), red (O), orange (P), dark gray (V).*

9.2.2 [(pyrNdipp)$_2$V(DMAP)$_2$] (3^{DMAP})

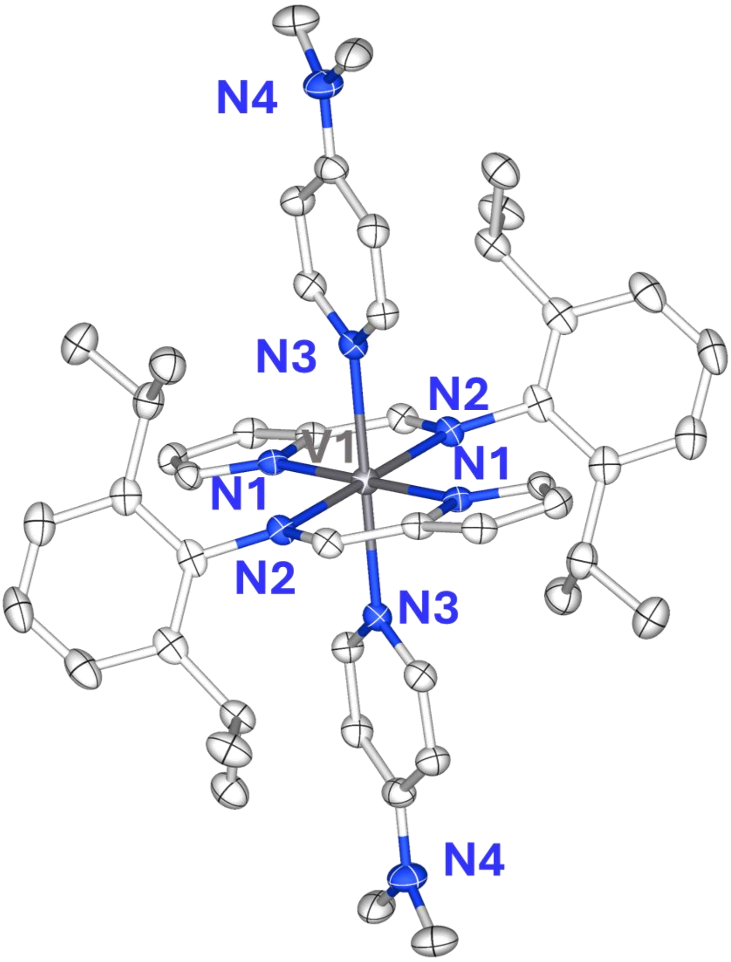

*Figure S22. Molecular structure of [(pyrNdipp)$_2$V(DMAP)$_2$] (**3**^{DMAP}) obtained through single crystal XRD, CCDC entry 2501032. Crystals grown by vapor diffusion from Et$_2$O and toluene, hydrogen atoms omitted for clarity, ellipsoids at 50% probability level. Color code: Light gray (C), blue (N), dark gray (V).*

9.2.3 [(pyrNdipp)$_2$V(THF)$_2$] (3^{THF})

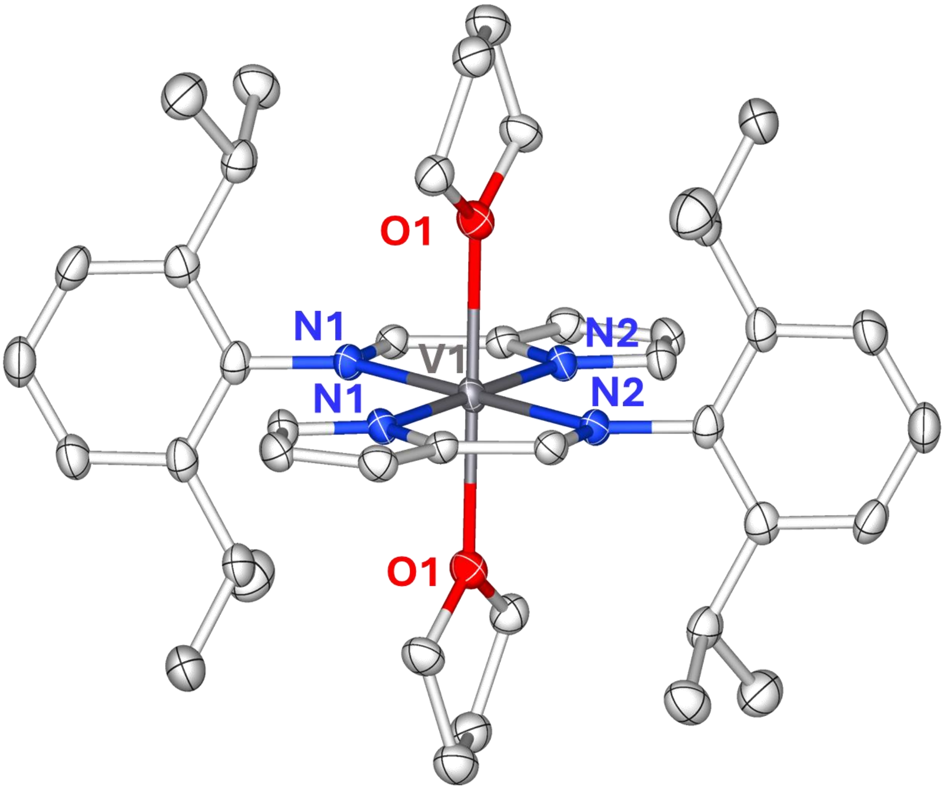

*Figure S23. Molecular structure of [(pyrNdipp)$_2$V(THF)$_2$] (**3^{THF}**) obtained through single crystal XRD, CCDC entry 2501033. Crystals grown by vapor diffusion from Et$_2$O and toluene, hydrogen atoms omitted for clarity, ellipsoids at 50% probability level. Light gray (C), blue (N), red (O), dark gray (V).*

9.2.4 [(pyrNdipp)$_2$V=P=V(pyrNdipp)$_2$] (4)

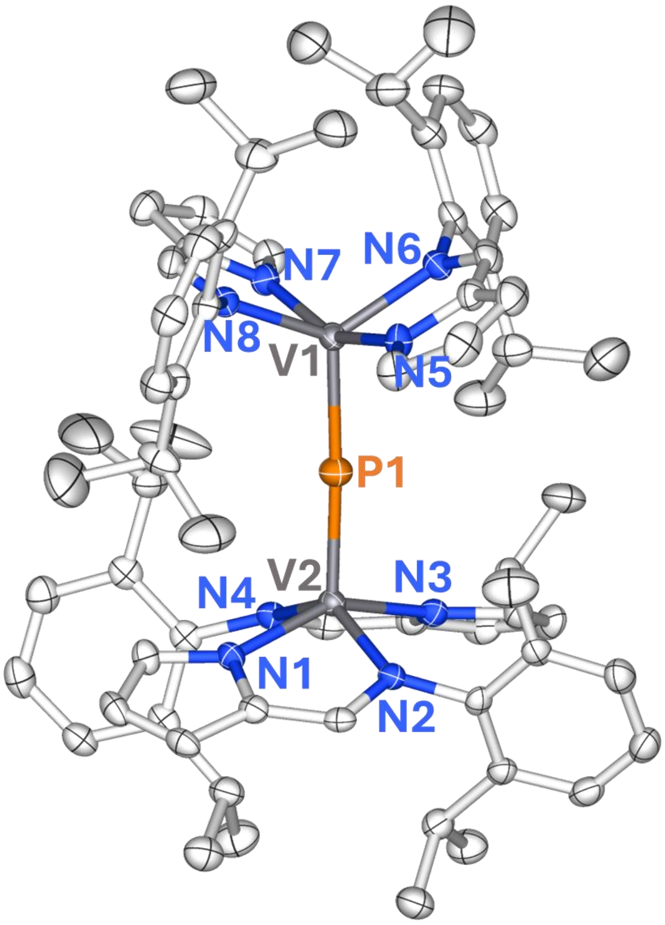

*Figure S24. Molecular structure of [(pyrNdipp)$_2$V=P=V(pyrNdipp)$_2$] (**4**) obtained through single crystal XRD, CCDC entry 2501034. Crystals grown by vapor diffusion from Et_2O and toluene. Hydrogen atoms and co-crystallized toluene are omitted for clarity, ellipsoids at 50% probability level. Light gray (C), blue (N), orange (P), dark gray (V).*

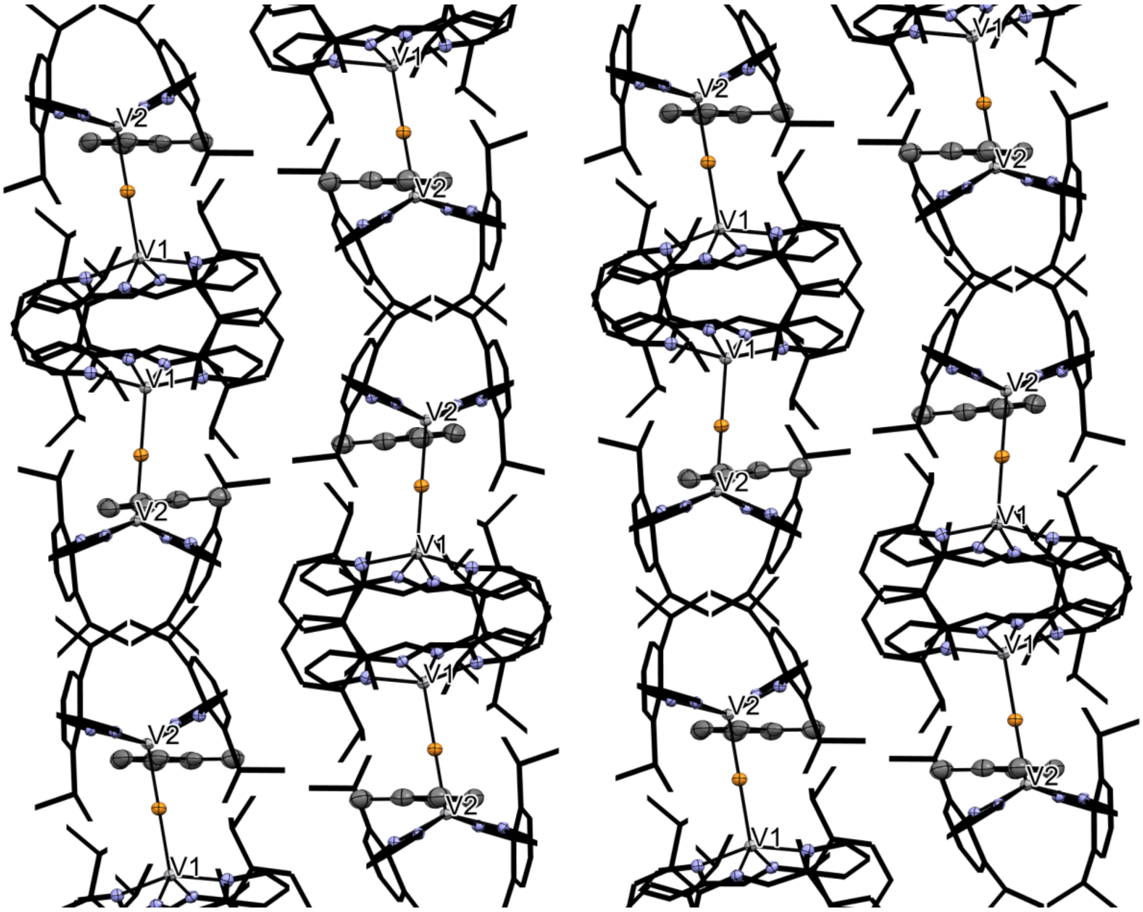

*Figure S25. Crystal packing of complex [(pyrNdipp)$_2$V=P=V(pyrNdipp)$_2$]·toluene (**4·toluene**) viewed along crystallographic c axis (toluene is observed closer to one vanadium atom, V2).*

9.2.5 [(pyrNdipp)$_3$V] (5)

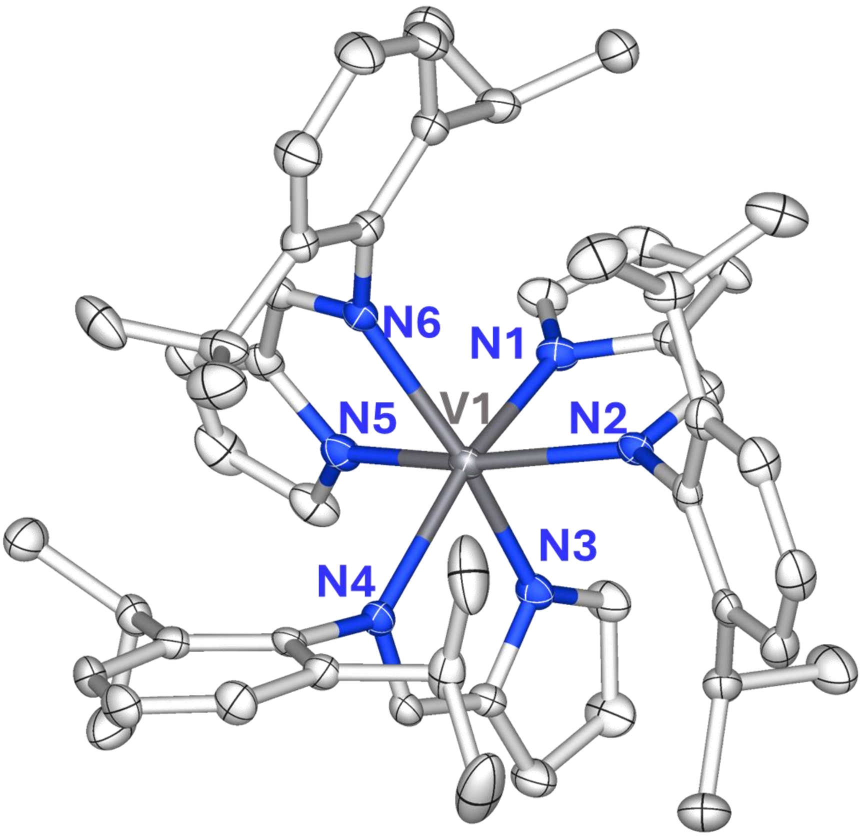

*Figure S26. Molecular structure of [(pyrNdipp)$_3$V] (**5**) obtained through single crystal XRD, CCDC entry 2501035. Crystals grown by vapor diffusion from Et_2O and toluene, hydrogen atoms omitted for clarity, ellipsoids at 50% probability level. Light gray (C), blue (N), dark gray (V).*

10. Computational Studies

10.1 Computational methodology

Density functional theory calculations were performed using the ORCA software version 6.0.0.[16–18] Gas-phase geometry optimizations were performed on non-truncated models using the hybrid Perdew–Burke–Ernzerhof generalized gradient functional, PBE0[19] combined with the def2-TZVP(-f)[20,21] basis set and the auxiliary basis set def2-J.[20] Dispersion effects were included in the calculations using Grimme's D3 method in combination with the Becke-Johnson (D3BJ) damping scheme.[22,23] We set up the convergence strategy to SlowConv with a TightSCF convergence threshold (energy change, TolE = 1×10^{-8}; RMS density change, TolRMSP = 5×10^{-9}; maximum density change, TolMaxP = 1×10^{-7}; DIIS error convergence, TolErr = 5×10^{-7}). The RI-J approximation for the Coulomb integrals and the COSX numerical chain-of-sphere integration for the HF exchange integrals (RIJCOSX) were employed to speed up the calculations.[24] The SHARK integral generation and digestion engine was employed to handle numerical integration more efficiently.[25] Analytical frequency calculations at the same level of theory (PBE0-D3/def2-TZVP(-f)/defgrid2) and with the same geometry optimization setup were employed to confirm that the resulting equilibrium structures were actual minima on the potential energy surface. The gas-phase energies and wavefunctions for all species were refined using the meta-GGA hybrid TPSSh functional, in combination with the def2-TZVP basis set.[26–28] The electronic structures of the optimized equilibrium species were scrutinized, including quasi-restricted orbitals,[29,30] Mayer and Wiberg bond orders at the TPSSh-D3/def2-TZVP level of theory. Orbital composition analysis and Wiberg bond orders were performed using Multiwfn v. 3.8.[31] For the calculation of solution state Gibbs free energies for the chemical reactions evaluated (see below), we employed the universal solvation model based on solute electron density (SMD)[32] to account for the solution state energetics of each species. The ORCA default setup was employed for both solvents considered, toluene and tetrahydrofuran. TDDFT calculations were performed for species **(4)** using the TPSSh and CAM-B3LYP functionals with the D3 dispersion correction and the def2-TZVP basis set, as implemented in ORCA.

10.2 Computational data

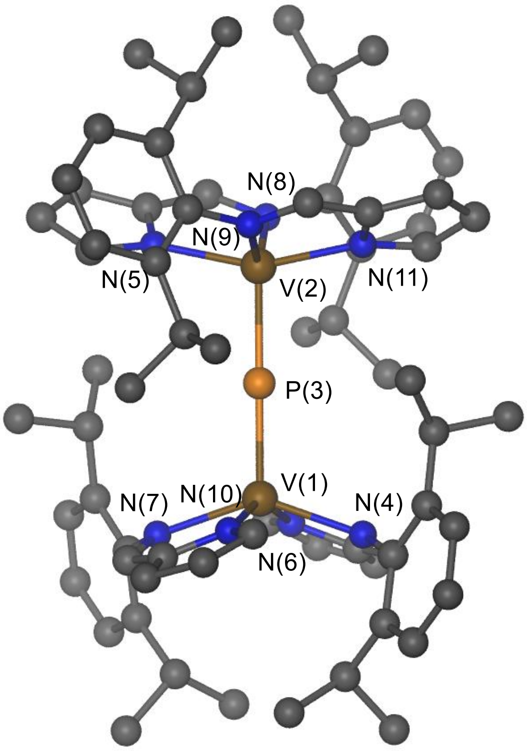

*Figure S27. DFT calculated molecular structure of **4***

Table S3. Calculated distances (Å) and angles (◦) of [(pyrNdipp)$_2$V=P=V(pyrNdipp)$_2$] (**4**). The schematic picture corresponds to the optimized quartet (*S* = 3/2) equilibrium structure. Hydrogen atoms are omitted for clarity. Geometries were optimized at the PBE0-D3/def2-TZVP(-f) level of theory

Metric	XRD	S = 1/2		S = 3/2	
		Calc.	Δ	Calc.	Δ
V(1)–P(3)	2.207	2.388	0.181	2.228	0.021
V(1)–N(4)	2.117	2.045	0.071	2.135	0.018
V(1)–N(6)	2.060	2.103	0.042	2.046	0.015
V(1)–N(7)	2.136	2.048	0.088	2.140	0.003
V(1)–N(10)	2.062	2.103	0.041	2.045	0.018
V(2)–P(3)	2.267	2.135	0.132	2.258	0.009
V(2)–N(5)	2.102	2.066	0.036	2.104	0.002
V(2)–N(8)	2.063	2.109	0.046	2.060	0.003
V(2)–N(9)	2.072	2.108	0.036	2.060	0.012
V(2)–N(11)	2.099	2.067	0.032	2.105	0.006
P(3)–V(1)–N(4)	107	115	8	109	2
P(3)–V(1)–N(6)	106	99	7	106	1
P(3)–V(1)–N(7)	113	115	2	109	3

P(3)–V(1)–N(10)	104	100	5	105	1
N(4)–V(1)–N(6)	78	79	1	78	0
N(4)–V(1)–N(7)	141	130	10	142	1
N(4)–V(1)–N(10)	92	93	0	92	1
N(6)–V(1)–N(7)	91	93	1	92	0
N(6)–V(1)–N(10)	150	161	11	149	1
N(7)–V(1)–N(10)	78	79	1	78	0
P(3)–V(2)–N(5)	101	105	4	101	1
P(3)–V(2)–N(8)	115	112	3	116	1
P(3)–V(2)–N(9)	115	112	4	115	0
P(3)–V(2)–N(11)	103	104	1	102	1
N(5)–V(2)–N(8)	79	78	1	79	0
N(5)–V(2)–N(9)	91	91	0	91	0
N(5)–V(2)–N(11)	156	151	5	157	1
N(8)–V(2)–N(9)	130	136	6	130	0
N(8)–V(2)–N(11)	91	91	0	91	0
N(9)–V(2)–N(11)	79	78	1	79	0

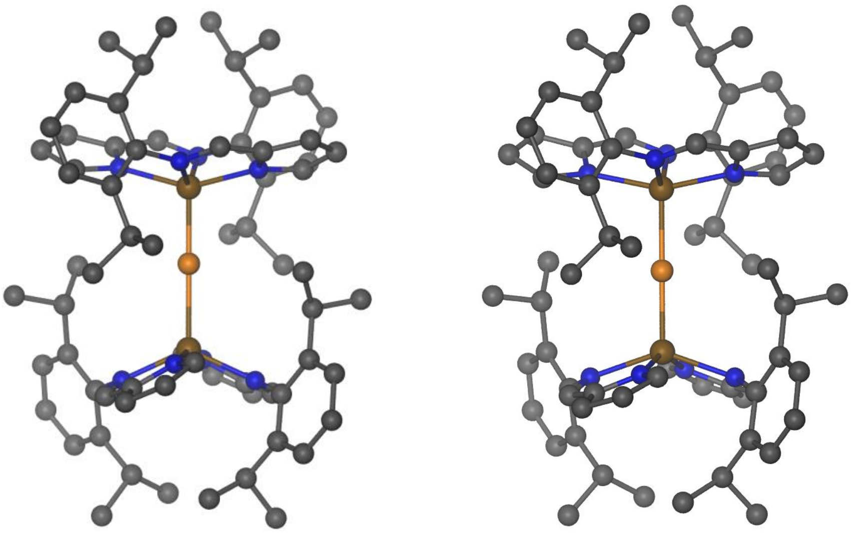

*Figure S28. Calculated equilibrium geometries for doublet (S = 1/2) and quartet (S = 3/2) states of [(pyrNdipp)$_2$V=P=V(pyrNdipp)$_2$] (**4**) together with their gas phase relative energies calculated at the TPSSh-D3/def2-TZVP level of theory.*

Table S4. Calculated Mayer and Wiberg bond orders of [(pyrNdipp)$_2$V=P=V(pyrNdipp)$_2$] (**4**, *S* = 3/2) at the TPSSh-D3/def2-TZVP level of theory

Bond	MBO	WBO
V(1) –V(2)	0.2627	0.3294
V(1)–P(3)	1.2336	1.8405
V(1)–N(4)	0.5468	0.5633
V(1)–N(6)	0.6254	0.7002
V(1)–N(7)	0.5516	0.5579
V(1)–N(10)	0.6308	0.7012
V(2)–P(3)	1.1157	1.6997
V(2)–N(5)	0.5583	0.6092
V(2)–N(8)	0.6147	0.6640
V(2)–N(9)	0.6094	0.6635
V(2)–N(11)	0.5622	0.6080

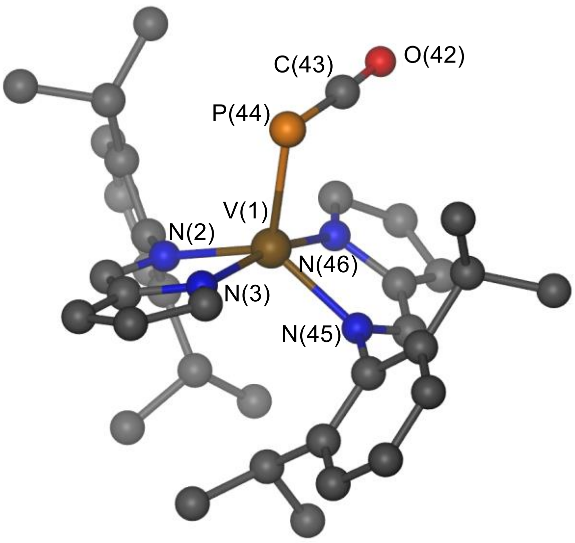

Table S5. Calculated distances (Å) and angles (◦) of [(pyrNdipp)$_2$V(PCO)] (**2**). The schematic picture corresponds to the optimized triplet (*S* = 1) equilibrium structure. Hydrogen atoms are omitted for clarity. Geometries were optimized at the PBE0-D3/def2-TZVP(-f) level of theory

Bond	XRD	Singlet		Triplet	
		Calculated	Δ	Calculated	Δ
V(1)–N(2)	2.038	2.065	0.027	2.029	0.010
V(1)–N(3)	2.089	2.024	0.065	2.098	0.008
V(1)–P(44)	2.391	2.236	0.156	2.390	0.001
V(1)–N(45)	2.038	2.066	0.027	2.036	0.002
V(1)–N(46)	2.089	2.065	0.024	2.088	0.001
P(44)–C(43)	1.660	1.724	0.064	1.664	0.004
C(43)–O(42)	1.161	1.181	0.020	1.157	0.004
N(2)–V(1)–N(3)	80	83	4	79	0
N(2)–V(1)–P(44)	115	104	11	113	2
N(2)–V(1)–N(45)	132	135	4	132	0
N(2)–V(1)–N(46)	94	87	7	96	1
P(44)–V(1)–N(45)	113	121	9	114	2
P(44)–V(1)–N(46)	108	112	4	107	2
N(45)–V(1)–N(46)	80	78	2	80	0
V(1)–P(44)–C(43)	87	62	25	91	5
P(44)–C(43)–O(42)	176	152	25	176	0

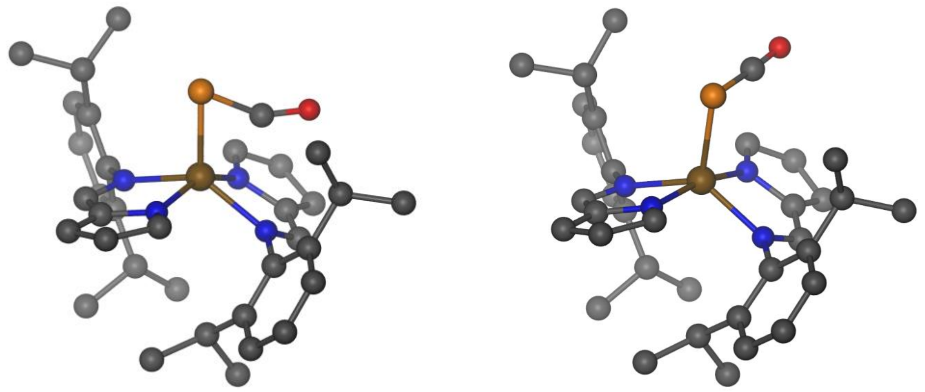

S = 0 ΔE^{gas} = 24.43 kcal/mol

S = 1 ΔE^{gas} = 0.00 kcal/mol

*Figure S29. Calculated equilibrium geometries for singlet (S = 0) and triplet (S = 1) states of [(pyrNdipp)$_2$V(PCO)] (**2**) together with their gas phase relative energies calculated at the TPSSh-D3/def2-TZVP level of theory.*

Table S6. Calculated Mayer and Wiberg bond orders of [(pyrNdipp)$_2$V(PCO)] (**2**, *S* = 1) at the TPSSh-D3/def2-TZVP level of theory

Bond	MBO	WBO
V(1)–N(2)	0.6651	0.7022
V(1)–N(3)	0.5763	0.6111
V(1)–P(44)	1.0309	1.2251
V(1)–N(45)	0.6449	0.6858
V(1)–N(46)	0.5893	0.6160
P(44)–C(43)	1.6698	2.1430
C(43)–O(42)	2.1074	2.8990

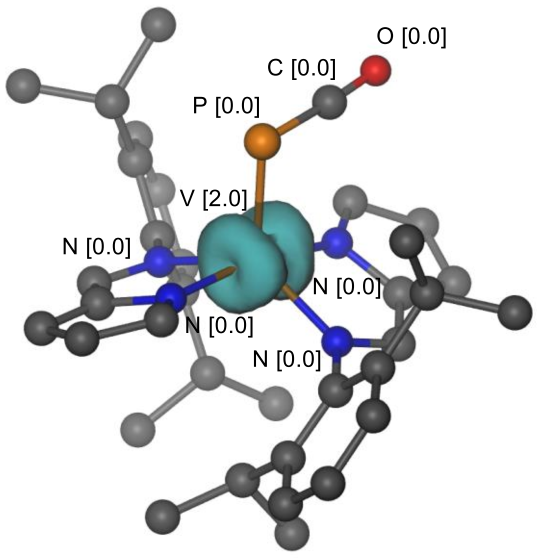

*Figure S30. Spin density plot and Löwdin atomic spin densities of triplet species [(pyrNdipp)$_2$V(PCO)] (**2**) (S = 1) at the TPSSh-D3/def2-TZVP level of theory. The isosurface is set to ± 0.03.*

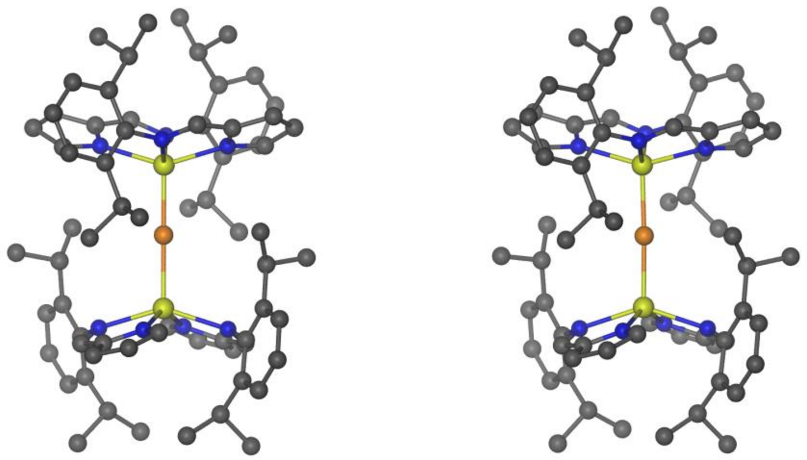

S = 1/2 | **S = 3/2**

ΔE^{gas} = 7.48 kcal/mol | ΔE^{gas} = 0.00 kcal/mol

Figure S31. Calculated equilibrium geometries for doublet (S = 1/2) and quartet (S = 3/2) states of [(pyrNdipp)$_2$Nb=P=Nb(pyrNdipp)$_2$] together with their gas phase relative energies calculated at the TPSSh-D3/def2-TZVP level of theory.

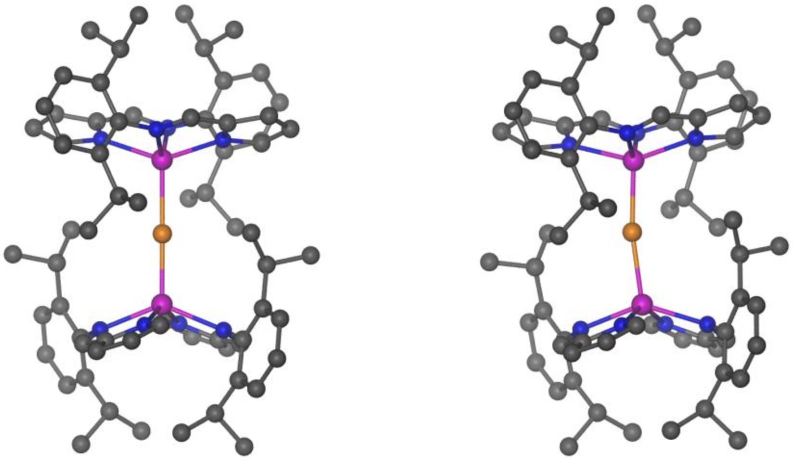

S = 1/2 — ΔE^{gas} = 0.00 kcal/mol

S = 3/2 — ΔE^{gas} = 1.36 kcal/mol

Figure S32. Calculated equilibrium geometries for doublet (S = 1/2) and quartet (S = 3/2) states of [(pyrNdipp)$_2$Ta=P=Ta(pyrNdipp)$_2$] together with their gas phase relative energies calculated at the TPSSh-D3/def2-TZVP level of theory.

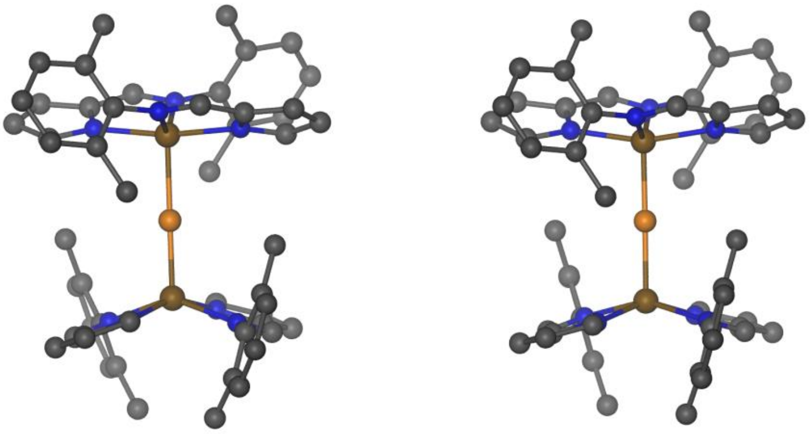

S = 1/2 — ΔE^{gas} = 11.24 kcal/mol

S = 3/2 — ΔE^{gas} = 0.00 kcal/mol

Figure S33. Calculated equilibrium geometries for doublet (S = 1/2) and quartet (S = 3/2) states of [(pyrNMe)$_2$V=P=V(pyrNMe)$_2$] together with their gas phase relative energies calculated at the TPSSh-D3/def2-TZVP level of theory.

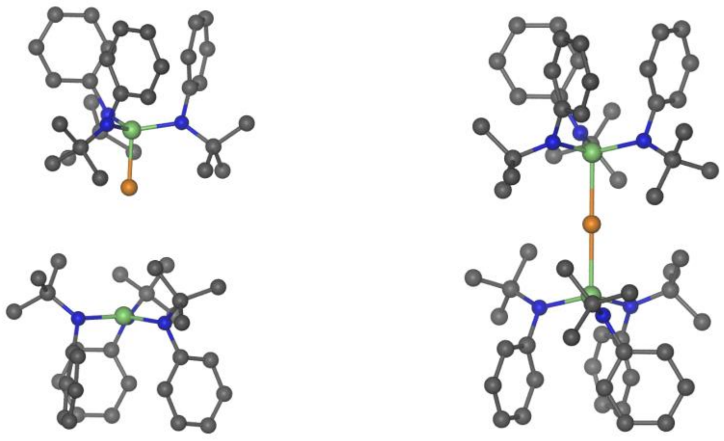

S = 1/2 ΔE^{gas} = 10.67 kcal/mol

S = 3/2 ΔE^{gas} = 0.00 kcal/mol

Figure S34. Calculated equilibrium geometries for doublet (S = 1/2) and quartet (S = 3/2) states of $[(^{t}BuPhN)_3Cr{=}P{=}Cr(N^{t}BuPh)_3]$ *together with their gas phase relative energies calculated at the TPSSh-D3/def2-TZVP level of theory.*

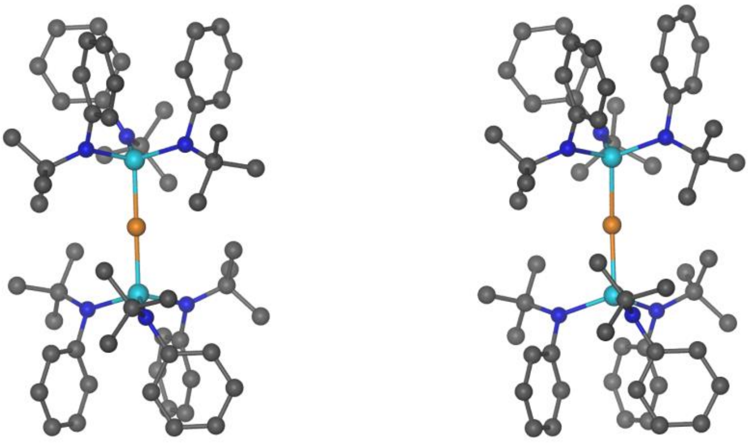

S = 1/2 ΔE^{gas} = 0.00 kcal/mol

S = 3/2 ΔE^{gas} = 12.96 kcal/mol

Figure S35. Calculated equilibrium geometries for doublet (S = 1/2) and quartet (S = 3/2) states of $[(^{t}BuPhN)_3Mo{=}P{=}Mo(N^{t}BuPh)_3]$ *together with their gas phase relative energies calculated at the TPSSh-D3/def2-TZVP level of theory.*

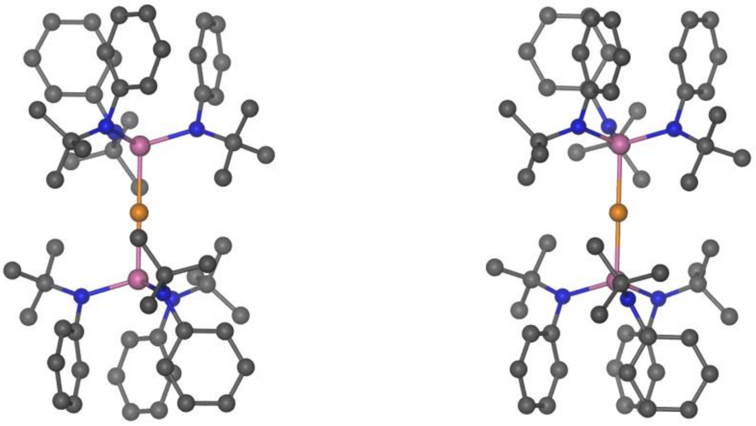

S = 1/2 — ΔE^{gas} = 0.00 kcal/mol

S = 3/2 — ΔE^{gas} = 11.79 kcal/mol

Figure S36. Calculated equilibrium geometries for doublet (S = 1/2) and quartet (S = 3/2) states of [(^{t}BuPhN)$_3$W=P=W(N^{t}BuPh)$_3$] together with their gas phase relative energies calculated at the TPSSh-D3/def2-TZVP level of theory.

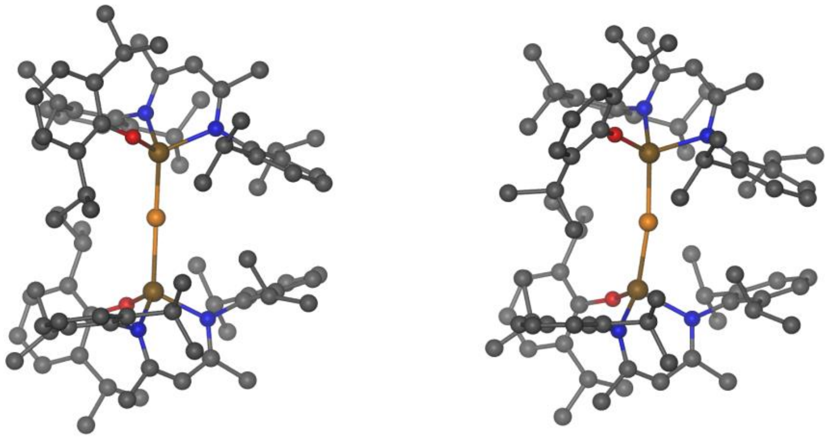

S = 1/2 — ΔE^{gas} = 4.29 kcal/mol

S = 3/2 — ΔE^{gas} = 0.00 kcal/mol

Figure S37. Calculated equilibrium geometries for doublet (S = 1/2) and quartet (S = 3/2) states of [(ArO)(nacnac)V=P=V(nacnac)(OAr)] together with their gas phase relative energies calculated at the TPSSh-D3/def2-TZVP level of theory.

Comments on calculated structures for 4 and other model systems.

To assess how the spin-state of an [M=P=M] complex is influenced by symmetry, coordination geometry, and sterics, as well as the nature of the *d*-orbitals of the metal centers, we calculated the relative energetics shown in **Figures S31–S37**. To select realistic model systems, we used Cummins' [(^{t}BuPhN)$_3$Mo=P=Mo(NPh^{t}Bu)$_3$] complex (*Angew.Chem., Int.Ed.* **1997**, *36*, 87) and analyzed the hypothetical series where Mo is substituted for Cr and W. Moreover, we constructed a hypothetical series from our own [(pyrNdipp)$_2$V=P=V(pyrNdipp)$_2$] complex with V substituted for Nb and Ta, and further used Mindiola's phosphaethynolate complex (*Chem. Commun.* **2019**, *55*, 5966), to model a hypothetical, trigonal, briding phosphide, [(nacnac)(Odipp)V=P=V(nacnac)(Odipp)].

Complex	Most Stable Spin State (Relative Energy, kcal mol^{-1})
[(pyrNdipp)$_2$V=P=V(pyrNdipp)$_2$]	Quartet (–10.79)
[(pyrNdipp)$_2$Nb=P=Nb(pyrNdipp)$_2$]	Quartet (–7.48)
[(pyrNdipp)$_2$Ta=P=Ta(pyrNdipp)$_2$]	Doublet (–1.36)
[(pyrNMe)$_2$V=P=V(pyrNMe)$_2$]	Quartet (–11.24)
[(nacnac)(Odipp)V=P=V(nacnac)(Odipp)]	Quartet (–4.29)
[{^{t}BuPhN}$_3$Cr=P=Cr(NPh^{t}Bu)$_3$]	Quartet (–10.67)
[{^{t}BuPhN}$_3$Mo=P=Mo(NPh^{t}Bu)$_3$]	Doublet (–12.96)
[{^{t}BuPhN}$_3$W=P=W(NPh^{t}Bu)$_3$]	Doublet (–11.79)

Among these model systems, all 3*d* complexes favor a high-spin, quartet state. The trigonal vanadium model, [(nacnac)(Odipp)V=P=V(nacnac)(Odipp)], possesses a relatively low doublet-quartet gap. To further address the structural influence of the ligand scaffold, we computed a methyl-substituted analog of our complex [(pyrNMe)$_2$V=P=V(pyrNMe)$_2$]. This model complex is also more stable in its quartet state relative to its doublet state, and the relative energies between doublet and quartet are essentially identical between the methyl and 2,6-diisopropylphenyl substituted systems.

The preferred spin-state varies between the tetragonal pyrNdipp$^-$ and the trigonal ^{t}BuPhN$^-$ ligated complexes. The niobium model system, [(pyrNdipp)$_2$Nb=P=Nb(pyrNdipp)$_2$], is a high-spin quartet, while the tantalum model system, [(pyrNdipp)$_2$Ta=P=Ta(pyrNdipp)$_2$], is likely a doublet (isoenergetic with quartet to within 1.4 kcal mol^{-1}). In contrast, the trigonal molybdenum and tungsten model systems, [{^{t}BuPhN}$_3$Mo=P=Mo(NPh^{t}Bu)$_3$] and [{^{t}BuPhN}$_3$W=P=W(NPh^{t}Bu)$_3$], are low-spin, doublet species.

In a three-fold symmetric complex, having pure σ-donors as supporting ligands, the δ combinations (from d_{xy} and $d_{x^2-y^2}$) should be non-bonding and low in energy. However, when inspecting the frontier orbitals (see Sections **10.5–10.14**, below) of model complexes [{^{t}BuPhN}$_3$M=P=M(NPh^{t}Bu)$_3$] (M = Cr, Mo, W), it is clear that the amido ligands π-donate into the d_{xy} and $d_{x^2-y^2}$ orbitals, such that these become high in energy. This leaves a σ-combination of two d_{z^2} orbitals among the lowest energetically accessible frontier orbitals. For the chromium complex, this orbital along with two π* orbitals are populated, to afford a relatively stable quartet state. In contrast, the spatial extent of the 4*d* and 5*d* orbitals of the molybdenum and tungsten congeners, leads to a greater overlap within the π* orbital sets, separating them energetically from the σ-set, and leading to a preference for low-spin in this case.

10.3 TD-DFT Calculations

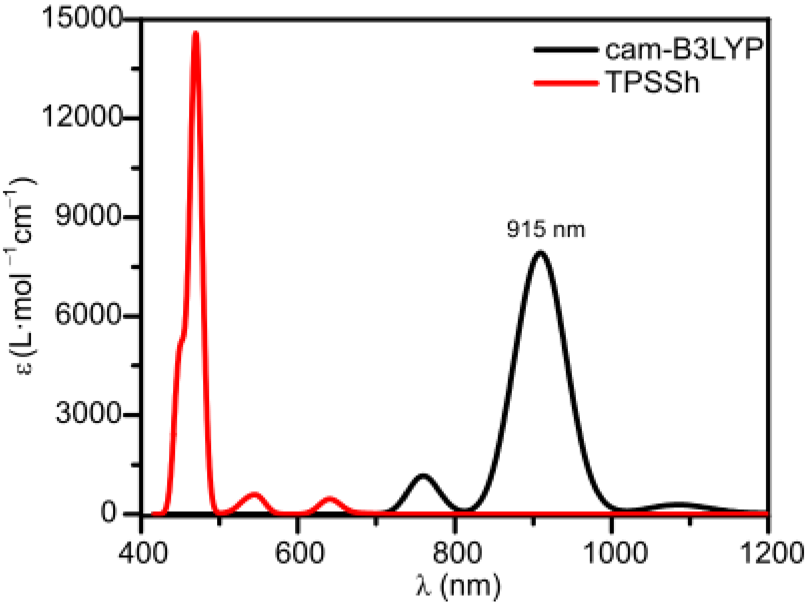

*Figure S38. TDDFT-Calculated UV–Vis Absorption Spectrum of [(pyrNdipp)$_2$V=P=V(pyrNdipp)$_2$] (**4**) (S = 3/2) at the TPSSh-D3/def2-TZVP and cam-B3LYP-D3/def2-TZVP levels of theory.*

Table S7. Major orbital contribution to the 915 nm electronic transition from TDDFT calculations calculated at the CAM-B3LYP-D3/def2-TZVP level of theory. Only transitions with weights higher than 10% are displayed.

**Transition**	**Transition Weight**
MO306 → MO307	0.60

10.4 Reaction Energies

Table S8. Calculated reaction Gibbs free energies (ΔG^0, 298.15 K) in the gas phase and solvent phase (THF and toluene). Energies are provided in kcal mol^{-1} and are calculated at the TPSSh-D3/def2-TZVP level of theory.

**Reaction**	**$\Delta G^{0,gas}$**	**$\Delta G^{0,THF}$**	**$\Delta G^{0,TOL}$**
OCPPCO → $^1/_2$ P_4 + 2 CO	-27.43	-21.83	-22.40
[(pyrNdipp)$_2$V(PCO)] → [(pyrNdipp)$_2$V≡P] + CO	34.83	37.51	37.26

10.5 Relevant Quasi-restricted Orbitals of [(pyrNdipp)$_2$V=P=V(pyrNdipp)$_2$] (4) (S=3/2) together with their Mulliken orbital composition analysis.

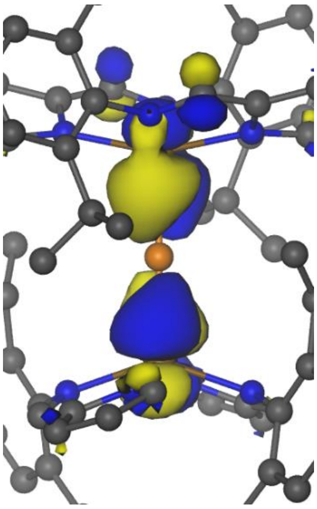

**LUMO**	V(1)	39.95
MO307	V(2)	33.62
-3.201 eV	P(3)	4.03

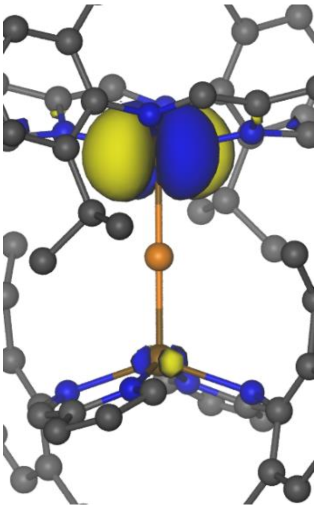

**SOMO 3**	V(1)	1.34
MO306	V(2)	94.70
-2.983 eV	P(3)	0.01

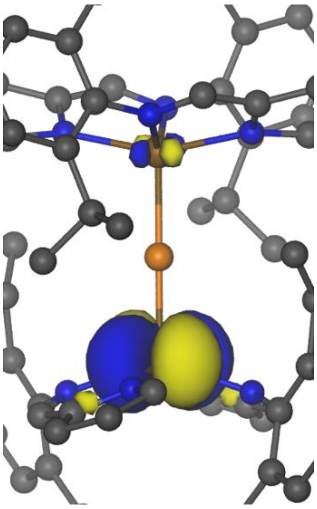

**SOMO 2**	V(1)	95.00
MO305	V(2)	1.37
-3.066 eV	P(3)	0.11

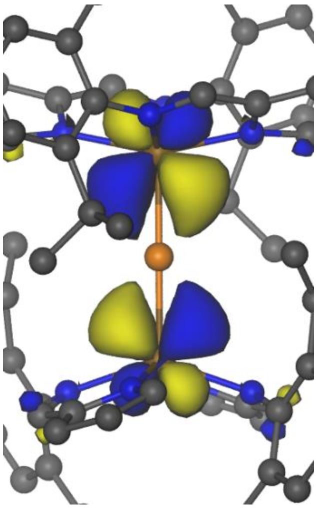

**SOMO 1**	V(1)	34.20
MO304	V(2)	53.18
-3.137 eV	P(3)	2.79

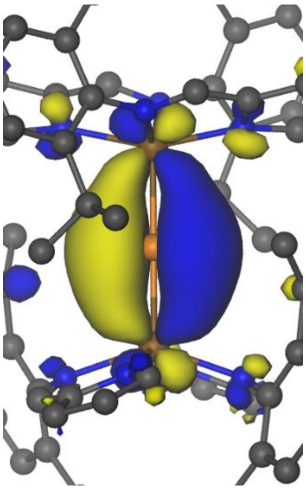

**HOMO**	V(1)	18.42
MO303	V(2)	12.04
-4.949 eV	P(3)	50.23

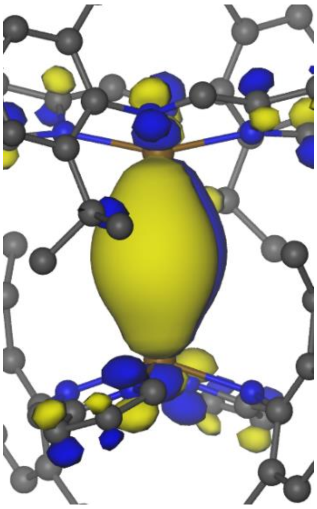

**HOMO – 1**	V(1)	10.14
MO302	V(2)	9.21
-5.125 eV	P(3)	54.08

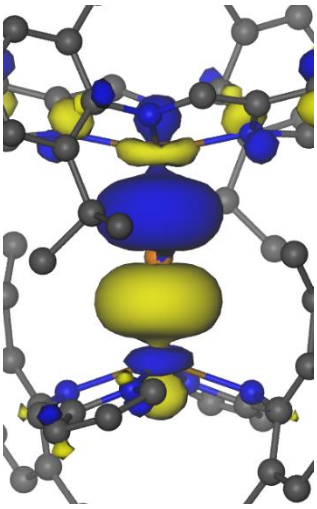

**HOMO – 18**	V(1)	18.42
MO285	V(2)	17.80
-6.682 eV	P(3)	34.94

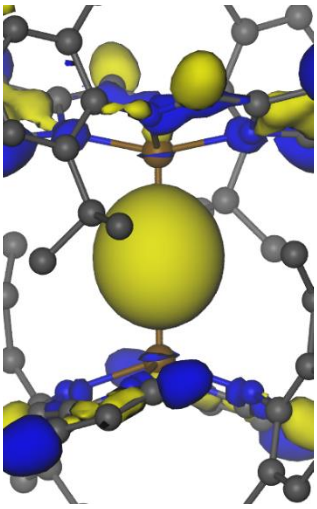

**HOMO – 129**	V(1)	1.95
MO174	V(2)	1.96
-13.682 eV	P(3)	36.03

10.6 Relevant Quasi-restricted Orbitals of [(pyrNdipp)$_2$Nb=P=Nb(pyrNdipp)$_2$] (S = 3/2)

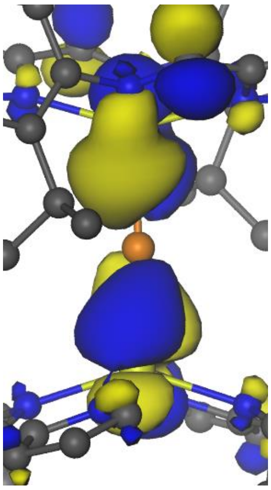

LUMO + 1

MO298

-2.613 eV

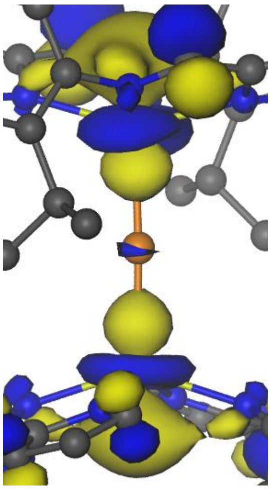

LUMO

MO297

-2.704 eV

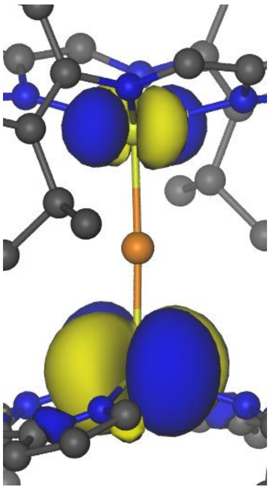

SOMO3

MO296

-2.524 eV

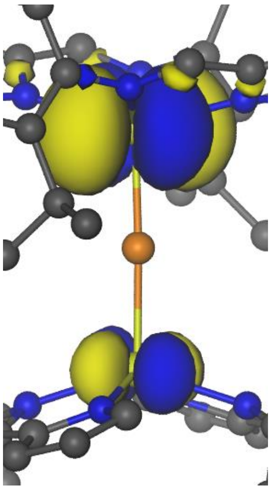

SOMO2

MO295

-2.596 eV

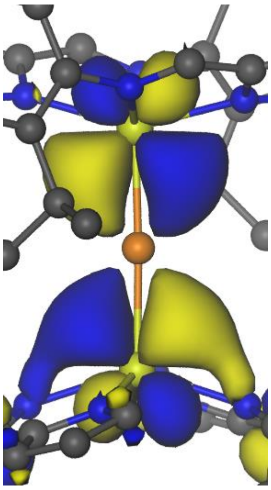

SOMO1

MO294

-2.778 eV

10.7 Relevant Quasi-restricted Orbitals of [(pyrNdipp)$_2$Ta=P=Ta(pyrNdipp)$_2$] (S = 3/2)

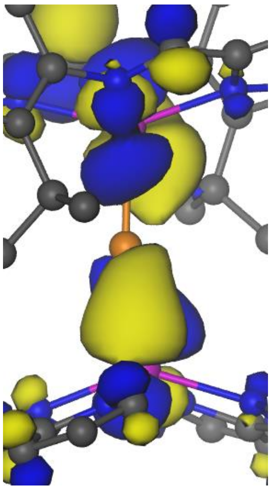

LUMO + 1

MO298

-2.524 eV

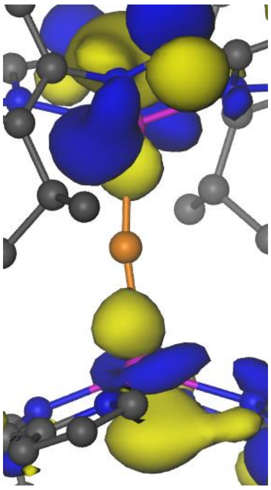

LUMO

MO297

-2.685 eV

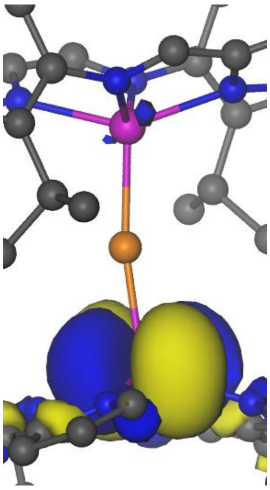

SOMO3

MO296

-2.326 eV

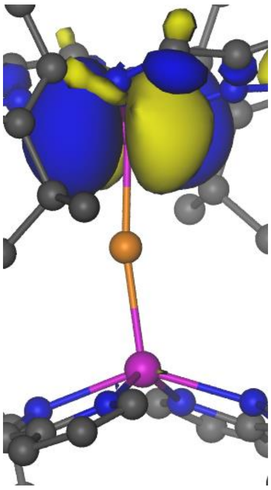

SOMO2

MO295

-2.452 eV

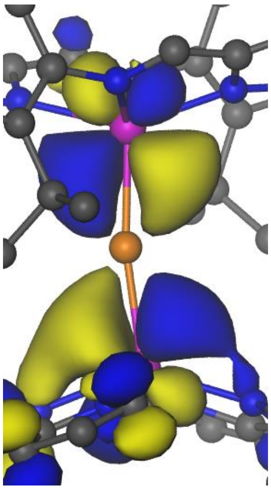

SOMO1

MO294

-2.638 eV

10.8 Relevant Quasi-restricted Orbitals of $[(^{t}BuPhN)_3Cr{=}P{=}Cr(N^{t}BuPh)_3]$ (S = 1/2)

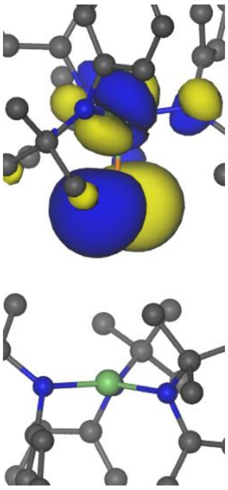

LUMO + 2

MO278

-3.063 eV

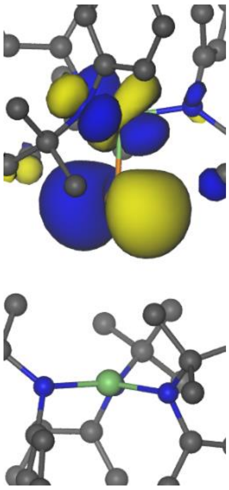

LUMO + 1

MO277

-3.073 eV

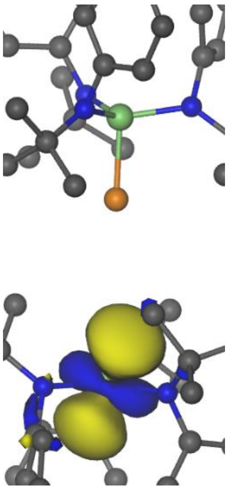

LUMO

MO276

-3.370 eV

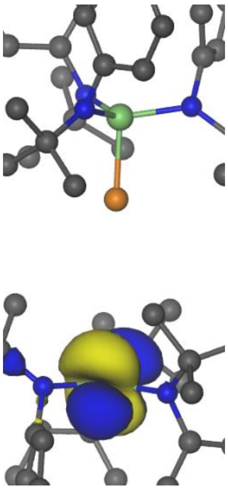

SOMO

MO275

-2.982 eV

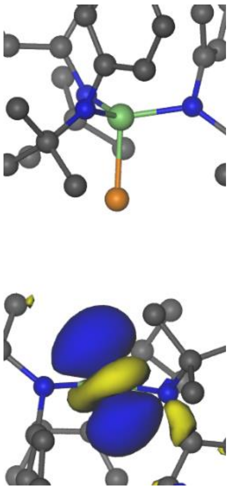

HOMO

MO274

-2.533 eV

10.9 Relevant Quasi-restricted Orbitals of $[(^t\text{BuPhN})_3\text{Cr}{=}\text{P}{=}\text{Cr}(\text{N}^t\text{BuPh})_3]$ (S = 3/2)

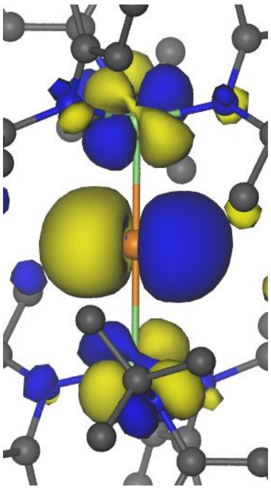

LUMO + 2

MO278

-3.357 eV

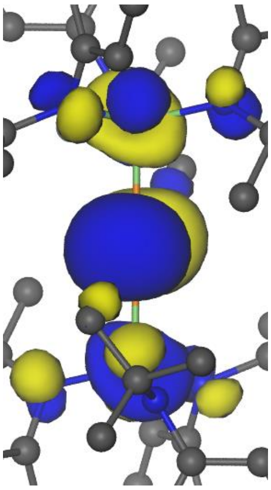

LUMO + 1

MO277

-3.370 eV

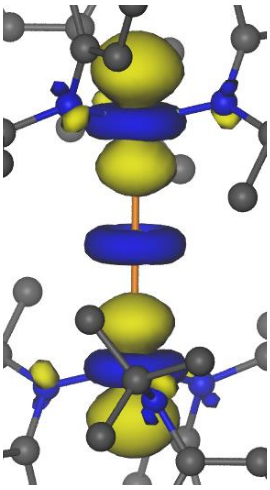

SOMO3

MO276

-2.873 eV

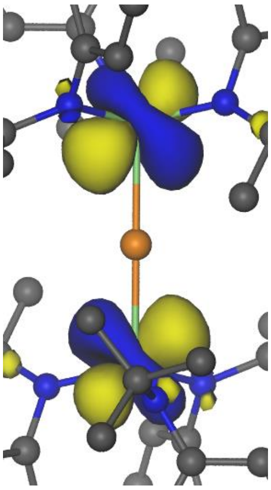

SOMO2

MO275

-3.526 eV

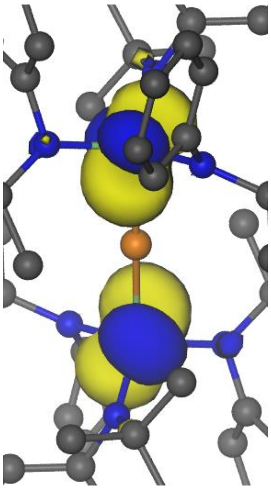

SOMO1

MO274

-3.536 eV

10.10 Relevant Quasi-restricted Orbitals of [(^{t}BuPhN)$_3$Mo=P=Mo(N^{t}BuPh)$_3$] (S = 1/2)

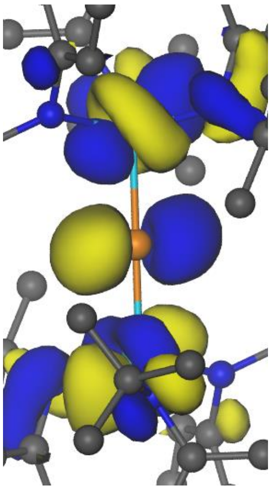

LUMO + 2

MO268

-1.456 eV

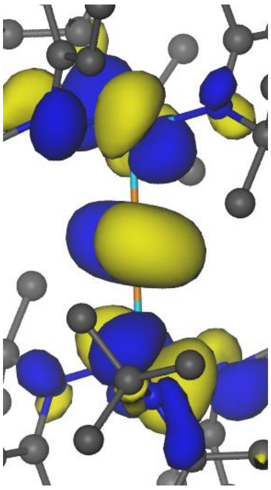

LUMO + 1

MO267

-1.580 eV

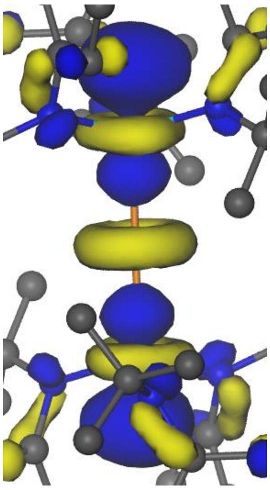

LUMO

MO266

-1.979 eV

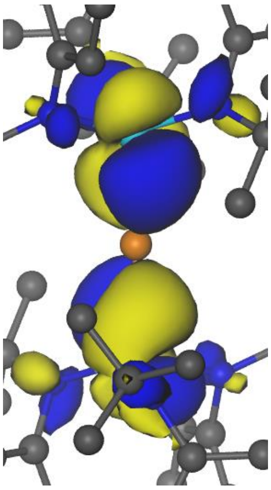

SOMO

MO265

-3.338 eV

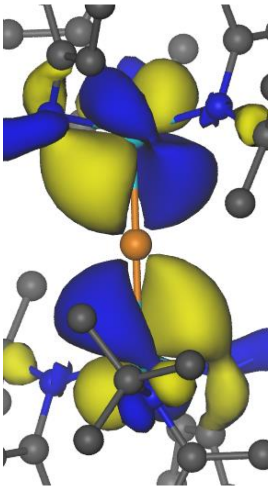

HOMO

MO264

-3.577 eV

10.11 Relevant Quasi-restricted Orbitals of [(^{t}BuPhN)$_3$Mo=P=Mo(N^{t}BuPh)$_3$] (S = 3/2)

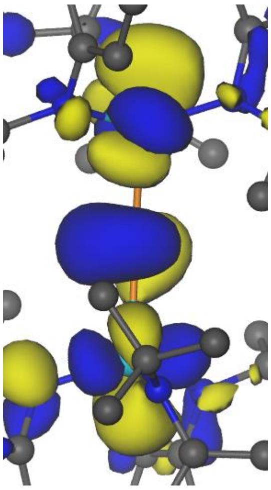

LUMO + 2

MO268

-1.859 eV

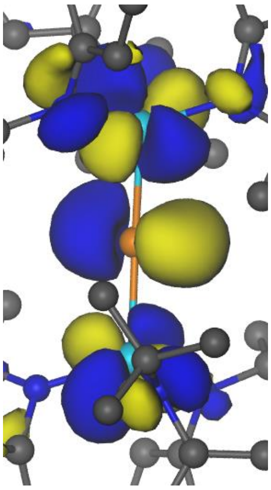

LUMO + 1

MO267

-1.951 eV

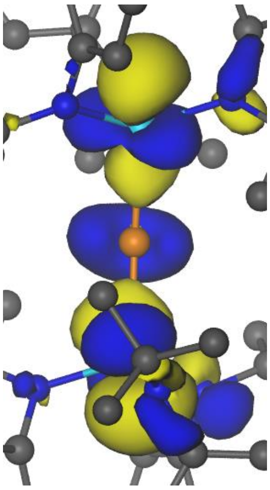

SOMO3

MO266

-2.457 eV

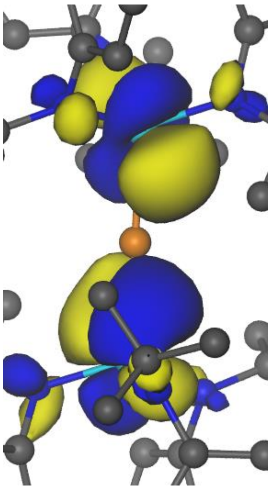

SOMO2

MO265

-3.305 eV

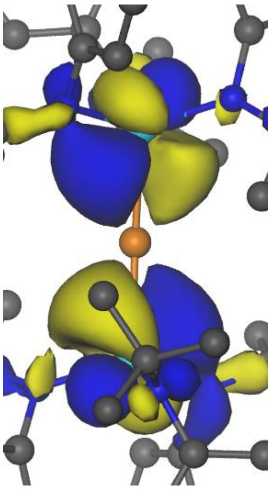

SOMO1

MO264

-3.325 eV

10.12 Relevant Quasi-restricted Orbitals of $[(^{t}BuPhN)_3W{=}P{=}W(N^{t}BuPh)_3]$ (S = 1/2)

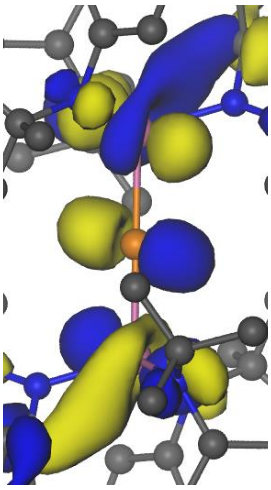

LUMO + 2

MO268

-1.184 eV

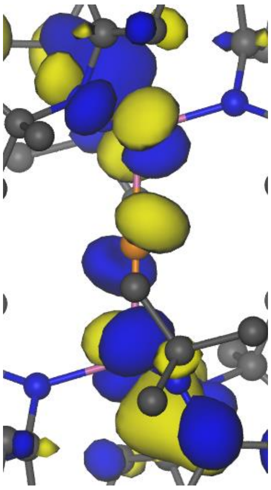

LUMO + 1

MO267

-1.235 eV

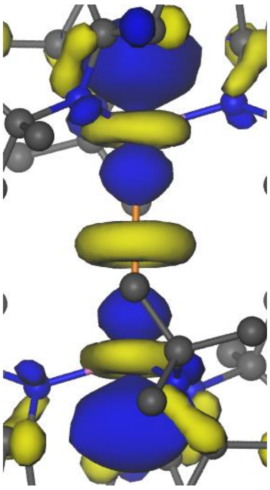

LUMO

MO266

-1.825 eV

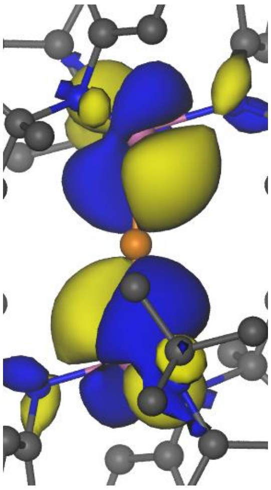

SOMO

MO265

-2.983 eV

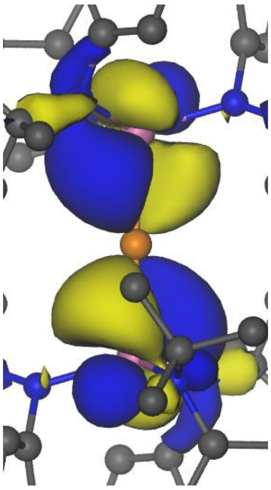

HOMO

MO264

-3.214 eV

10.13 Relevant Quasi-restricted Orbitals of $[(^{t}BuPhN)_3W{=}P{=}W(N^{t}BuPh)_3]$ (S = 3/2)

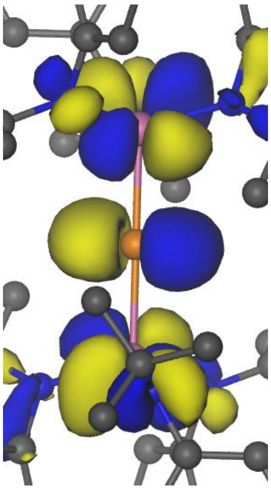

LUMO + 2

MO268

-1.549 eV

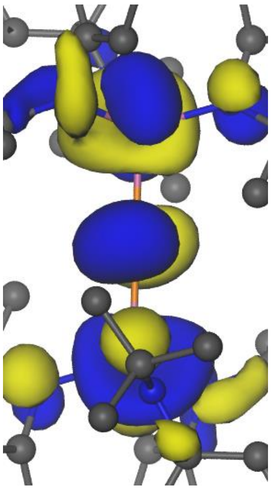

LUMO + 1

MO267

-1.570 eV

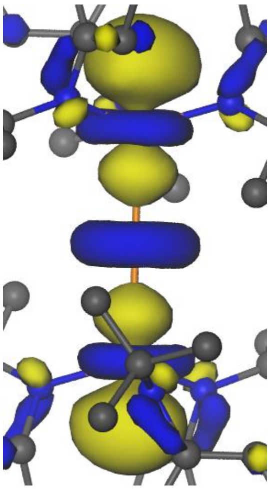

SOMO3

MO266

-2.109 eV

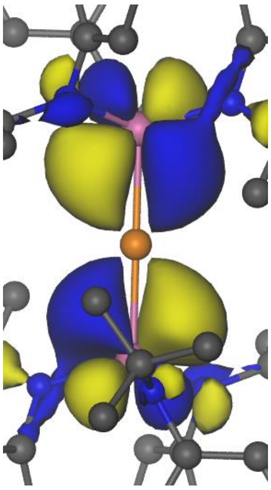

SOMO2

MO265

-3.155 eV

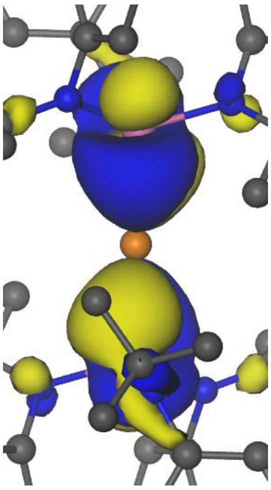

SOMO1

MO264

-3.167 eV

10.14 Relevant Quasi-restricted Orbitals of [(ArO)(nacnac)V=P=V(nacnac)(OAr)] (S = 3/2)

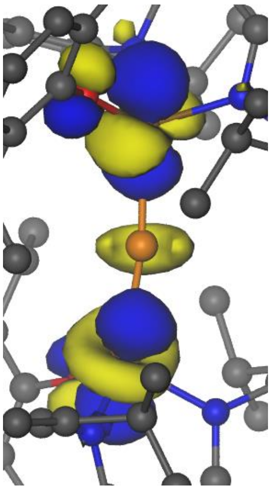

LUMO + 2

MO360

-2.258 eV

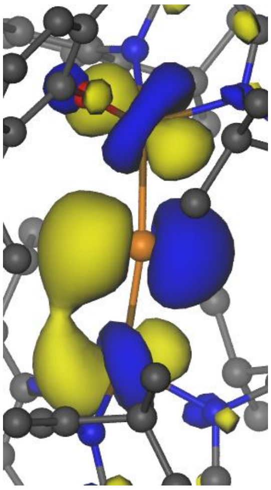

LUMO + 1

MO359

-2.987 eV

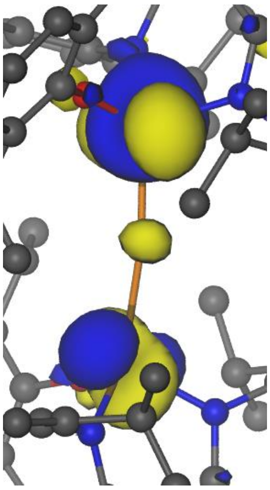

SOMO3

MO358

-2.789 eV

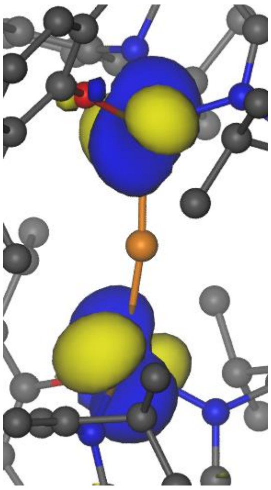

SOMO2

MO357

-3.118 eV

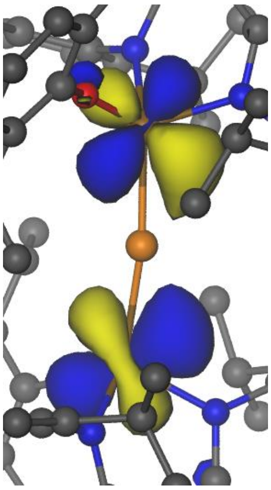

SOMO1

MO356

-3.215 eV

10.15 Cartesian coordinates

Cartesian coordinates of optimized structures (in Å) at PBE0-D3/def2-TZVP(-f)/defgrid2 level of theory. *S* is the spin multiplicity (*M* = 2*S* + 1).

[(pyrNdipp)$_2$V=P=V(pyrNdipp)$_2$] (**4**, *S* = 1/2)

```
Eh = -5306.513101652404
V    13.39342789803485    8.95298801718488    5.97972200965544
V    12.92614544114521   13.45037524078699    5.85600705928717
P    13.15065248707803   11.32810989589338    5.91266570276922
N    12.06472106937779    7.91405556114095    4.82254031560354
N    14.12874526528180   14.14006066777746    7.38797513920720
N    11.90680554132743    8.51020445744491    7.39920090330330
N    14.89353812998401    8.27895812610000    7.20056481653895
N    11.49579277511848   14.13577805264113    7.24568079668973
N    14.19423550225089   14.33034948568173    4.42028701491880
N    14.94986389181681    8.71813436322268    4.58489074879536
N    11.61615301978935   13.79051264426548    4.29351939595358
C    11.04489674691262    7.42893215575045    5.49068420992904
H    10.31313509004127    6.80224962808563    4.98339460491716
C    10.92707550496790    7.71281318148898    6.84791636971593
C    16.00064635248575    7.98140672195424    6.56233443965492
H    16.84353642562755    7.56023622711026    7.10802118750733
C    12.02509988891129   14.65016264018174    8.31805138715956
H    11.39163753380970   15.10176795736117    9.08042176205866
C    13.41940290304654   14.64593137155886    8.45129646767230
C    16.07128638757352    8.19213865721084    5.18853722651501
C    12.01510506290151    8.11366873250172    1.13670763008495
H    11.73085939307935    8.81121793932401    0.35732499457712
C    12.21405310152643    7.55902157670789    3.45371994443645
C    13.57965747903198   14.65506914231869    3.31973747927577
H    14.11520515376263   15.17124457759295    2.52419834660971
C    11.61759024778413    8.60726905014597    8.69705692768326
H    12.24550359043567    9.17141317620545    9.36997150543847
C    14.63924396534941    7.39174043085833   11.28480237878220
H    14.57795115408828    7.16217262356204   12.34228475437672
C    12.21610780228626   14.36012962824534    3.19572944563836
C    11.85276630189073    8.48255375351801    2.46741212492137
C    14.47547689279268    6.38946488927613   10.34685594688516
H    14.27597909564642    5.37603187471316   10.67603122399595
C    10.10459945362095   14.33008212712727    7.03675832763938
C    12.53459180114879    6.87989060438704    0.79203250888429
H    12.65441992036197    6.61231271413016   -0.25140184112884
C    15.42562110969393   14.25505659543754    7.71169187182872
H    16.20321718175261   13.93513109666459    7.03542806809415
C    15.51732238407412   14.81308326289046    4.60406491405546
C     9.21976455228256   13.27058482167612    7.25435261888800
C    16.52548055368158    8.31542233579401    3.01476988130644
H    16.99259622419827    8.26323731709667    2.04361722681245
C    11.30033590928704    9.84573005482012    2.80602048033675
H    11.37382308578503    9.98410588454600    3.88731028007417
C    10.45458287159426    7.88417410009039    9.01457292745562
H    10.00742719797747    7.80126546872998    9.99294948869777
C    12.90784462043085    5.99114104758205    1.78349035751111
H    13.32995906066406    5.03078065233663    1.51034454980358
C    17.88109093881364   14.45479213624906    4.62478954073100
H    18.73500326815086   13.80277981830181    4.48061099364693
C    15.57364793253968   14.83437940982786    8.97536315071302
H    16.50917084536953   15.04100349058165    9.47165275555310
C    15.69786881848566   16.14264618615800    5.01209759838494
C    14.55285822268086    6.65984967663964    8.98666413319176
C    12.76086387041568    6.31138052971140    3.12664694214536
C    14.28925383445277   15.09046244607415    9.44974332917702
```

H	14.00915126480404	15.53987323313382	10.39061558530783
C	7.86553609257633	13.49033212467073	7.02953039182293
H	7.16243108809633	12.68437571669844	7.20370006279287
C	14.81217906071130	7.97724532042715	8.58794488637692
C	8.29698079280951	15.74388044813997	6.35373666107395
H	7.93232464133697	16.69717623863081	5.98898597949882
C	15.22869337188725	8.78665811565938	3.28253307483420
H	14.50138280308915	9.15042479829990	2.57254937378927
C	13.20435005068144	5.32684908870520	4.18300231093112
H	13.12597851901105	5.82115472401744	5.15289063578861
C	12.98181057158448	4.87165342706515	8.19418208117767
H	12.93553890064901	4.35273471193595	9.15475498983905
H	12.80484214700675	4.13091919220932	7.41019643676765
H	12.17132360247423	5.60145818459936	8.16788962241778
C	18.08251032341788	15.76931278690915	5.00179675722358
H	19.08827413145323	16.14495333798274	5.15067124525697
C	16.99509776491841	16.60155818090688	5.19710449837528
H	17.15631401197924	17.62647927405202	5.51052570241042
C	10.61325028154820	16.70420632541563	6.25815219453853
H	11.62773825438276	16.30456849036051	6.30678580363339
C	14.33473224819003	5.54488959732484	7.99077353211736
H	14.33272392368446	5.98453396389908	6.99202512449929
C	7.40213232874994	14.71617995905513	6.58847699460577
H	6.34235927340646	14.86884655496789	6.41992427771191
C	16.60076029061469	13.94965549541253	4.42549261690959
C	16.41165425106195	12.50757260002611	4.02599512216795
H	15.34184545477097	12.28899015422413	4.05461457756318
C	10.01130265627137	7.31160350893610	7.83345925229728
H	9.14591786311451	6.68303102543701	7.68610136742015
C	11.28337720760274	14.54830425065833	2.17324025007248
H	11.47390916580598	14.98300056066732	1.20349737291023
C	14.87367318936209	8.68949500394818	10.86909945682362
H	14.99442798008183	9.47339178322900	11.60734068485695
C	12.29159638559884	4.10438912384588	4.21510471608082
H	11.25421599956488	4.38395964657327	4.41217528472551
H	12.60948012375193	3.40566335794023	4.99305683899422
H	12.31632833096623	3.57640779688177	3.25846983581536
C	10.07495179342050	14.06416290659224	2.66881759889222
H	9.12185318519812	14.04056901901049	2.16355245329570
C	10.32677561454448	13.61254380289967	3.96760234006912
H	9.62536847927293	13.18364091707765	4.66648239290110
C	15.47108677359003	4.52782311739469	8.03875702034211
H	16.43541144411066	4.99500282346852	7.82625608404007
H	15.30905950483581	3.73369292555604	7.30544625996406
H	15.53864701044031	4.06367071494223	9.02594336458320
C	9.65914335042120	15.57202979501774	6.56315117698897
C	17.06611209236301	7.93459407974567	4.23244899255359
H	18.04482655113535	7.52019268878081	4.42230383891899
C	14.95603149889481	9.01106459134780	9.51935072110494
C	10.41441722709088	17.25603414153783	4.85096791610870
H	10.47839388855128	16.46444249807234	4.10307380688241
H	11.18156361252177	18.00205184535043	4.62940417095777
H	9.44295541383909	17.74515945469645	4.74532477025773
C	12.11607534503128	10.95706913290956	2.15673469421011
H	12.13638127554259	10.86029231448635	1.06820159272736
H	13.14528126452931	10.95491570583634	2.52131525648942
H	11.68462487772145	11.92762195390955	2.39519715337336
C	14.52956505183621	17.06799239964380	5.26505236029963
H	13.62257971596893	16.46031547448465	5.27897200600419
C	15.21059925221890	10.43718030183864	9.09573043964281
H	15.02923037090592	10.50840584880446	8.02028472298953
C	9.70484976709693	11.92569643204685	7.73384452115533
H	10.79126727530911	11.90494194011296	7.62005775738909
C	10.50401005244562	17.81212202567722	7.30188358880582
H	9.49426712391043	18.23071338125994	7.31776843219417
H	11.20124830307928	18.62343174179814	7.07790466031278
H	10.72518162782045	17.44275229339810	8.30568063392080
C	14.66224799949967	4.92039934076258	3.99806599453639

```
H    14.80330111179320    4.34958734857140    3.07700944505398
H    14.98615880657775    4.28810626762605    4.82870408245440
H    15.31406408450337    5.79425867818458    3.95563527132700
C    16.90493190632471   12.26771895459779    2.60232692402204
H    16.38395671849719   12.91199012773832    1.88975436155485
H    16.73702714260160   11.22755953133855    2.31427659436388
H    17.97567680278032   12.47268937624211    2.51449122012423
C     9.82579115873850    9.95000005895095    2.42787751032952
H     9.43561219368713   10.93166869922172    2.70560820766382
H     9.23061445580317    9.18502233251639    2.93260098047748
H     9.68631275663902    9.82431036994505    1.35062795314558
C    14.62503309968560   17.76026916765188    6.61997438402473
H    15.46835420323062   18.45410899181627    6.65854825400262
H    14.74298459045010   17.03608859922604    7.42694011482158
H    13.71759402022456   18.33981109953785    6.80718157412357
C    17.08760712366158   11.56697722429681    5.01557222679928
H    18.15944325577077   11.76569089482346    5.09604394443895
H    16.96543040343254   10.53335526776460    4.69489637709013
H    16.64566732823880   11.66520142280934    6.00982423918376
C     9.14634283943145   10.78788869004806    6.89018857730341
H     8.05623433654531   10.73194778890986    6.94793509077817
H     9.54239172483514    9.83513067642116    7.23840412656995
H     9.42676440160868   10.90578156658477    5.84061671126919
C     9.38111395915614   11.73442551584033    9.21265404937686
H     9.83670144736528   12.51918593186863    9.82173579278160
H     9.75248886998438   10.76820143478970    9.56114142505908
H     8.30126713601159   11.76430740928738    9.38395325463229
C    14.38534845776963   18.08886142106481    4.13969293992331
H    13.53362742149991   18.74803894374963    4.32473341447709
H    14.23536399339939   17.60423578758813    3.17263490393095
H    15.28240631871556   18.70919096512880    4.06467651484310
C    14.25827577579508   11.41573305225098    9.77019727314640
H    13.22008908766164   11.20284712781903    9.50666999687095
H    14.47849932934222   12.43164014005118    9.44679254977477
H    14.34847821717092   11.38965124187063   10.85891801309252
C    16.66651064366210   10.82483854305409    9.33873990503818
H    16.91709991093250   10.76537339450378   10.40164699117982
H    16.84291655486484   11.84987808818267    9.00518483112639
H    17.34880656882155   10.16443893215278    8.79771428812245
```

[(pyrNdipp)$_2$V=P=V(pyrNdipp)$_2$] (**4**, *S* = 3/2)

```
Eh = -5306.520687280069
V    13.39640129778940    8.92301752700944    5.99636085475671
V    12.93670253737168   13.38338934284803    5.85297977280040
P    13.17824907565919   11.13875370861262    5.90867135858964
N    12.04614957896013    8.04078295192935    4.59727767393829
N    14.30866742198819   13.98334711111423    7.33084300273496
N    11.90258680009187    8.25456183708216    7.22379887272203
N    14.89044435892010    8.42167193671886    7.44341611830183
N    11.66905769498968   14.17722755560706    7.26961612110924
N    14.03089906138527   14.33329372589433    4.38828976318015
N    15.00266651176680    8.49740069610627    4.80521226320187
N    11.48038872575568   13.61653014898623    4.35089477766109
C    11.01357626528273    7.50046812854065    5.17132318583656
H    10.29468036011600    6.92776515745715    4.58680567336901
C    10.87256848644351    7.62526805206590    6.56133742966688
C    16.01302453372859    8.06552642498306    6.89472577533493
H    16.82852573874382    7.68072788043840    7.50620027409422
C    12.28693226541804   14.70370057956955    8.29851054829782
H    11.71341043839650   15.20657123933026    9.07548624408992
C    13.67538846154403   14.62379512929953    8.37150057984105
C    16.13507446794653    8.13415539895722    5.49866784576771
C    12.38373546645026    8.22437931814110    0.92624360681895
H    12.20936643396642    8.92540733287392    0.11837483261263
C    12.30988875587180    7.67676174973517    3.25188944260083
```

C	13.32960779599531	14.65992624648623	3.33106781991742
H	13.79808137618370	15.21521999258492	2.52007062628787
C	11.56918125454993	8.24823915500224	8.52850708654622
H	12.21341086627264	8.68502087453943	9.27513532268951
C	14.20871894996064	7.43343193566300	11.44937567125782
H	14.02280784808772	7.17798710833769	12.48618098674029
C	11.98482320773499	14.30262951052815	3.26981745358332
C	12.08922799727309	8.60382113832509	2.23066179475036
C	14.20970429456911	6.44624338695401	10.48204828535632
H	14.01146578431721	5.41902685365578	10.76609025673070
C	10.26143614740849	14.35737005485149	7.17131859716876
C	12.89520629254192	6.97065857888777	0.64423413241183
H	13.11914843386476	6.69243398555976	-0.37909608745158
C	15.61416668545201	14.04589968074615	7.59566291338971
H	16.34341284067343	13.62784568206197	6.91808686105069
C	15.37097232385251	14.80378521815217	4.47076935363476
C	9.41236436158552	13.29035825197049	7.48084643213539
C	16.66796699218540	8.09693843671515	3.33829795082574
H	17.18736010797393	8.00031800596527	2.39759212928400
C	11.53383692061719	9.97439642268037	2.52357755007132
H	11.56452717431363	10.11941553352481	3.60622357799992
C	10.34285565088502	7.61555372989011	8.72240224181283
H	9.84737461326557	7.47373948159778	9.67025399468504
C	13.12884581320495	6.07562863380038	1.67264616580619
H	13.54763394159015	5.10142365628225	1.44784558933602
C	17.72276348221604	14.41694789151291	4.28134306903781
H	18.55295351571056	13.75451877080955	4.06523055664480
C	15.85455425218801	14.72064439513091	8.80498088722927
H	16.82185418605783	14.90715824128981	9.24511354379002
C	15.60341257160279	16.13088563871466	4.85615143194239
C	14.44098135416404	6.74883602909859	9.14545749531887
C	12.85031971488798	6.40827899025421	2.99200010296839
C	14.61525301140537	15.09187732832662	9.30268675339602
H	14.40115605584293	15.63052323029678	10.21358171063509
C	8.04072461821957	13.49396102591931	7.37792472779705
H	7.36674691364416	12.68106364593391	7.62238913211542
C	14.69151085960838	8.08619660686001	8.80683951055066
C	8.38105040951435	15.74472837501968	6.64677470597528
H	7.97319707060607	16.69117297406515	6.31067053032696
C	15.33726389081512	8.47757287451065	3.50111275239531
H	14.62143722074659	8.72620474271479	2.73354075970121
C	13.15415450441633	5.41955256175402	4.09653341151993
H	13.04277105990763	5.93772026379311	5.05115222027074
C	13.06363391386829	4.88288943452374	8.15840050620798
H	12.94515061238551	4.34416087068834	9.10166117714139
H	13.03287788051277	4.14466971574083	7.35279054551181
H	12.21086658553869	5.55341481021122	8.04369756051577
C	17.97251661338862	15.72994824883354	4.63355500268237
H	18.99196843583312	16.09377448860126	4.68981591652053
C	16.91715285958813	16.57496926107641	4.92429114192970
H	17.11652736308181	17.59814033668293	5.22108141240271
C	10.66410679437015	16.73142955696900	6.33467191547335
H	11.68731435272316	16.35281920915362	6.32486394426037
C	14.38313619438692	5.64667145209341	8.11050873881731
H	14.45020658992354	6.10597640285070	7.12284031797379
C	7.52374888517432	14.70913030988349	6.97003295823373
H	6.45125347444578	14.84809119736063	6.89763121258234
C	16.42440230816719	13.92506823705538	4.20133145217263
C	16.18887864776655	12.48021717580896	3.83524139441011
H	15.13083198061670	12.25853582279074	3.99230821715129
C	9.89664507359816	7.20909516852007	7.46382210435908
H	8.98407797189250	6.68442660191526	7.22393033203625
C	10.98299499009501	14.50851092085782	2.30925012878567
H	11.09661485741130	15.01950336437457	1.36496981397856
C	14.43428264523023	8.75096881553273	11.08960389676228
H	14.41961872948283	9.52204697761228	11.85004504064910
C	12.16174867637518	4.25964433252850	4.08419323863233
H	11.13273494305588	4.60662098301888	4.20003816693018

H	12.37735065002859	3.56086510522432	4.89634336667306
H	12.22179539070211	3.71087472274489	3.14074750261014
C	9.83747905585414	13.92692821328008	2.83032802228374
H	8.85766606712176	13.88753145597084	2.38022950231333
C	10.19254890673284	13.39541052245840	4.08170713392472
H	9.55217107730024	12.88434320984474	4.78494000708249
C	15.56874121957986	4.69563902980241	8.25035424400898
H	16.52054505784725	5.22291114488384	8.15537613361524
H	15.53103024779500	3.92100443885076	7.48037950929835
H	15.55980986739718	4.20232387368538	9.22571582211788
C	9.75829740599680	15.58997246579991	6.73273294265677
C	17.18031710826036	7.86934312099571	4.61668328981974
H	18.17915063730385	7.55727281397995	4.88231753024873
C	14.67312059537700	9.10262927799432	9.76725564894225
C	10.35405005301365	17.23583703900820	4.92953924695944
H	10.37628458663466	16.42213673717813	4.20316184694207
H	11.08965643412935	17.98595739513566	4.62875770741608
H	9.36835126210988	17.70516939840383	4.88222260829980
C	12.37985448043171	11.07458115862237	1.89764461095003
H	12.41169312610943	10.99288477299008	0.80805456138422
H	13.40546730652883	11.04095901325481	2.27235390699904
H	11.96292082776908	12.05060962086627	2.14300065225686
C	14.47453238867205	17.06918006772451	5.21246243605707
H	13.56476671902695	16.47167509887136	5.29410925487170
C	14.92548765452100	10.53426981300244	9.37093490117539
H	14.67659861915276	10.62540770990189	8.30944906353447
C	9.93820308298362	11.94495078430283	7.91801584326856
H	11.02524430108896	11.95349116674915	7.81354826995688
C	10.60378730342587	17.86873706139266	7.35037872748726
H	9.59088841234220	18.27367504631185	7.42030775026401
H	11.27127072584098	18.68277702125924	7.05655675047462
H	10.89556312302393	17.53372024517117	8.34826618110155
C	14.58852310491250	4.90587011077823	4.03341339024602
H	14.76311919188919	4.31091795187090	3.13365386033486
H	14.79142267372543	4.26285433480635	4.89391649253132
H	15.30603686942541	5.72746234509222	4.04053973693324
C	16.50087590030662	12.22546576029642	2.36390042784202
H	15.88624206190408	12.85214418698196	1.71302165864434
H	16.30956084084865	11.17909604415063	2.11378129177712
H	17.54983288894829	12.43900819192060	2.14010558479935
C	10.07653892791736	10.07261811662668	2.08166335017172
H	9.67445966204773	11.05746823821270	2.32811367776220
H	9.46202974586081	9.31422459512302	2.57314182978629
H	9.98395023852627	9.92754551500928	1.00152575810623
C	14.69630277248752	17.74136062361795	6.56257457680816
H	15.53253497377831	18.44414712856457	6.52759457960870
H	14.90339517195882	17.00571529616792	7.34078304537519
H	13.80658235763778	18.30658102455110	6.85113466491265
C	16.97827545275821	11.54020540062446	4.73696945659455
H	18.05603296637236	11.69466941621879	4.64044718727831
H	16.76908056199420	10.50496524634744	4.47418172171147
H	16.70609835274046	11.67886651197850	5.78540577649440
C	9.41565146803541	10.82664776403343	7.02526576492889
H	8.32391020760291	10.77397466011688	7.03806566073675
H	9.79776415055661	9.86610564379139	7.36534150098276
H	9.74070210114910	10.96493461299890	5.99169745336516
C	9.61673331335712	11.67855545381609	9.38557693776542
H	10.03928105409069	12.45257573281020	10.03095880702322
H	10.02724549830137	10.71316353908938	9.69034286709996
H	8.53703293372936	11.65383880069246	9.55697113483816
C	14.25128160965284	18.10806753309947	4.11692756226330
H	13.43420967555668	18.78197988259597	4.38640510597273
H	14.00418461574087	17.64127530772002	3.16100959455741
H	15.15093284457007	18.71119490656738	3.96931690058662
C	14.04532035295983	11.51778188228666	10.12656188430240
H	12.98880242619899	11.25516910926102	10.03061904352201
H	14.18264802611097	12.52119177397950	9.72565997752113
H	14.29179945302004	11.55601009120780	11.19101614756274

```
C    16.40435982824388   10.87850616952594    9.52829727099536
H    16.71852065953472   10.76685023926572   10.56999009157667
H    16.58573189180253   11.91134489625282    9.22400636657841
H    17.03050899622784   10.22392064753114    8.91733472402316
```

[(pyrNdipp)$_2$V(PCO)] (**2**, *S* = 0)

```
Eh = -2937.074193370713
V     8.04162447084715    7.82281061920701   12.24363986378179
N     8.96393935253245    6.98770514839692   10.59502998942853
N     6.34859525836814    7.36944071146497   11.23079703612314
C     6.69735442529782    6.89943360367219    9.98077467257171
C     8.05561752872545    6.69034857402286    9.70495731838672
H     8.35570963164745    6.27434289391921    8.74380580348428
C    10.32442475599535    6.69439120240299   10.30675932983532
C     9.98307121921209    4.46706105502358   11.46637191132847
H     9.12326803486936    5.01204580406170   11.86127242283401
C     4.48401763288018    7.16256859590950    9.98267377378208
H     3.44076867619567    7.18787674884325    9.70961186954785
C     5.55549793807810    6.75965703826664    9.18919870593147
H     5.52616432949215    6.40886744584469    8.16841152586279
C    10.85132020079569    5.47513933155720   10.75203883296354
C     5.01647751538644    7.52980049372688   11.21917676005094
H     4.48976254436398    7.88249354539174   12.09337450919233
C    11.11165306248810    7.64260527416717    9.64852272448145
C    10.55512449987263    8.96776649282016    9.18555515296614
H     9.51864514229031    9.03124982448984    9.52071071444512
C    12.19103212231760    5.21313191796896   10.50376429879254
H    12.62131454302026    4.27801302238912   10.84082676282205
C     9.45923915047663    3.41940217462372   10.48782732776888
H     8.87892027097868    3.87526713646139    9.68227995973681
H     8.81781978792204    2.69901495436267   11.00176565705602
H    10.28714717959143    2.87062631693686   10.03123307642981
C    12.98858288750943    6.13481361184220    9.84748860884232
H    14.03470786440662    5.91617105064985    9.66754863210721
C    12.45057390561975    7.33631197263648    9.42713762225021
H    13.08099550000338    8.05640027889722    8.91790625289482
C    10.67943508860556    3.81478750876617   12.65256719848247
H    11.50334176663165    3.16978353975173   12.33705057746433
H     9.97008336800434    3.18959319769263   13.19973447836102
H    11.07071410544744    4.56711698833784   13.33877683497733
C    11.30138008392444   10.13788917867668    9.81735798069067
H    11.26094610338613   10.08561334368303   10.90714328116562
H    10.84967682221244   11.08443655075365    9.51143247494228
H    12.35134649483439   10.15470034615466    9.51468204463795
C    10.55846773114772    9.06434894563153    7.66276304915400
H    11.57554098798687    9.02087696665765    7.26456783128630
H    10.11149124763371   10.00753305181805    7.33948647591914
H     9.99157767224025    8.24663834841767    7.21142306216651
O     8.73250641822260    9.79114650711417   14.47826125142034
C     8.42787917479683    9.53436856123307   13.36615613343130
P     7.99150344067042   10.00620345420658   11.76608335664954
N     7.21692430232593    7.09386185209332   13.99181793930463
N     9.72126601231129    7.30984347408377   13.33001146803172
C     9.43901159574705    6.85567218184060   14.60055251719985
C     8.08806565417616    6.71920431000816   14.89677993716795
H     7.74633507186740    6.30193596355809   15.84119765154210
C     5.84152237719947    6.79492885683519   14.20791867705111
C     6.25094328111861    4.55388879685216   13.08675017571687
H     7.13244146888058    5.09411710568421   12.73373534856310
C    11.64798570256283    7.07773566833964   14.47557644545038
H    12.70664370482486    7.08903915821630   14.68350631993420
C    10.62666259286191    6.68643182144371   15.32599391602025
H    10.71114743597985    6.31805196974446   16.33694016638001
C     5.34587142623601    5.56507924542858   13.75010946032207
C    11.04783893754631    7.45627010609203   13.26389540563152
```

```
H    11.53363575194049    7.81549551537070   12.36981279504347
C     5.01046452712536    7.73709343898477   14.82075807778855
C     5.53057678062199    9.06419158344632   15.31532319491059
H     6.61172394912655    9.06855585711547   15.19232281246777
C     3.99695534193852    5.29414360374447   13.92647475086001
H     3.59308613152235    4.35041369464407   13.58055599471119
C     6.72703356963276    3.50789440130031   14.09094072350142
H     7.26805261058103    3.96470534135684   14.92207800189037
H     7.39332802823206    2.78805636042248   13.60865105362602
H     5.87795915048036    2.95882600304590   14.50638170292153
C     3.15514563860050    6.21759319077204   14.52280110532370
H     2.10173193988815    5.99297021716526   14.64348389093600
C     3.66155648631259    7.42477372988414   14.96340130226832
H     2.99996812481944    8.14464046658221   15.43160416243059
C     5.61263656525018    3.89569047106024   11.87080248113216
H     4.77714728650451    3.24952043295316   12.15092791043203
H     6.34677743493423    3.26991334150833   11.35820492798133
H     5.24715943241148    4.64285297489868   11.16504828893092
C     4.96624780915212   10.21724249109617   14.49010321153065
H     5.20138318982716   10.09873985855677   13.43030660052895
H     5.39489899409502   11.16567099794965   14.82182353254882
H     3.87927302750684   10.28215739954904   14.59274776354677
C     5.24409742434450    9.26198306601671   16.80060333490137
H     4.17114326050919    9.31556015735012   17.00242434890508
H     5.69509466272299   10.19428623658894   17.14784811211366
H     5.65576638135026    8.44381232956331   17.39573334400278
```

[(pyrNdipp)$_2$V(PCO)] (**2**, *S* = 1)

```
Eh = -2937.128853093571
V     8.05816552559917    7.71570762008415   12.23199113249844
N     8.88523933331563    6.86679566993599   10.58529030916631
N     6.35847394361123    7.44984616873694   11.03149474152545
C     6.69145752203975    6.91192264931845    9.80683200862931
C     8.03733089173192    6.62122375152099    9.61111076262898
H     8.39526654199377    6.20199451891546    8.67233132853555
C    10.27334623344587    6.63683806065841   10.39602204932580
C     9.98570974784513    4.48232017405853   11.68498060589545
H     9.12267787804698    5.03177895893247   12.07098590668551
C     4.49156405338281    7.25058731923610    9.77723084041364
H     3.45010923958338    7.30639542821617    9.50088461410383
C     5.54184071099383    6.77840633462475    9.00889257325110
H     5.50083922386660    6.38354652856464    8.00483313404016
C    10.84282353921969    5.48064580448873   10.94578569700748
C     5.04312436113976    7.65185298258038   11.00905416061577
H     4.52540493075263    8.06083680878807   11.86552277866235
C    11.04661251465492    7.59728931682993    9.73576181662433
C    10.44543843468298    8.87255533460902    9.19295066271728
H     9.42583571720566    8.95277341114503    9.57428995868977
C    12.20459958401471    5.27882402274767   10.78038173501263
H    12.66761402228031    4.39228189779876   11.19586145958307
C     9.45815709909717    3.41405455161267   10.73114816180164
H     8.88214758219363    3.85457456004870    9.91435081605466
H     8.81144273207388    2.70916609696462   11.25966672911112
H    10.28548002745712    2.85153190421039   10.29067518017465
C    12.98452140599796    6.20260381746012   10.10421577015434
H    14.04796341731971    6.03011599439936    9.98578320974354
C    12.40902866544165    7.35047704926906    9.59431818602513
H    13.02860008850890    8.07750519811788    9.08204207300179
C    10.68661278489393    3.86019559212604   12.88386066630254
H    11.50757430898249    3.20494585029546   12.58195799385222
H     9.97908824171500    3.25126230510281   13.45118363375910
H    11.08343833068922    4.63016676300693   13.54775659904680
C    11.19558700340742   10.10760000787381    9.67991896158827
H    11.24640870521294   10.13561789771661   10.77006391089874
H    10.68308115168647   11.01246355985273    9.34601353572511
```

```
H    12.21659316889561   10.14157139577998    9.29167141456334
C    10.37433545102069    8.83910847033703    7.66875780423324
H    11.37341612161657    8.76434055725227    7.23077053466290
H     9.90514348349674    9.74937073435814    7.28775056571896
H     9.79389372091498    7.98431490065422    7.31363823363794
O     9.95071349701165   10.73107744128776   13.51921522893622
C     9.02674732219131   10.41051185900024   12.90095208380798
P     7.68375902092376   10.06332130412883   11.98103672360161
N     7.20849890128332    6.90984493345710   13.89788476906003
N     9.74900543646048    7.50665609252786   13.43976611781162
C     9.40689029021386    6.99608538517625   14.67453238603128
C     8.06048721237327    6.70402003094588   14.87636394645208
H     7.70894317557022    6.30868963452800   15.82801758547498
C     5.82352498655391    6.67916222922189   14.10210560274895
C     6.10616828056224    4.51973401203584   12.82187007610007
H     6.95597372442860    5.07243526039598   12.41182104793609
C    11.61535158754532    7.27881953567665   14.69050602646548
H    12.65829033055174    7.31457658820681   14.96388677009973
C    10.55370327290264    6.84813696883368   15.47046356031363
H    10.58729392268812    6.46836980464488   16.48066664734440
C     5.25077200198231    5.52150528320158   13.55841334819136
C    11.07169730343085    7.67082846605024   13.45601511582683
H    11.59840597374917    8.05539881953818   12.59398129227497
C     5.05568511354558    7.63529122795194   14.77523156218748
C     5.65869689850045    8.90757585785203   15.32453400111486
H     6.67333053416680    8.99577628650491   14.93171515554095
C     3.89089459975805    5.31751167982784   13.73526912075097
H     3.42595327887315    4.43012204584037   13.32395845881279
C     6.65979533829346    3.47443866588160   13.78665301407093
H     7.24331243890600    3.93525164249189   14.58670204120639
H     7.30600694831790    2.76716645874365   13.26070591157430
H     5.84535245545544    2.91114204185264   14.24956656000451
C     3.11586565600764    6.23888441617938   14.42036018711906
H     2.05383873157724    6.06472214051205   14.54863255304073
C     3.69509958467964    7.38523693862107   14.92916311849002
H     3.07972882564476    8.10839468786542   15.45183538735976
C     5.39438580878614    3.86683475251258   11.64632908728388
H     4.58713949675220    3.20661507161849   11.97362542195576
H     6.10068895055726    3.25626268219415   11.07926968271334
H     4.97701408017181    4.61855518199466   10.97420254298291
C     4.89808483237724   10.14503461569898   14.86141319898698
H     4.85744827723537   10.19447563086587   13.77180374105351
H     5.40031151250179   11.04771452723740   15.21649472770201
H     3.87535733512371   10.16075255805738   15.24595089331328
C     5.74666319244025    8.85787074234014   16.84768729148568
H     4.75215490401476    8.77910316139292   17.29513096787131
H     6.21998981582702    9.76402432823542   17.23334003199063
H     6.32975071003414    7.99929604063937   17.18879075724133
```

[(pyrNdipp)$_2$Nb=P=Nb(pyrNdipp)$_2$] (S = 1/2)

```
Eh = -3532.892688763162
Nb    13.40452036852146    8.80761950682220    5.98522175975793
Nb    12.93645972592929   13.40228367823812    5.86533284923008
P    13.17337709386998   11.15349804342595    5.90541979050050
N    11.93901633827319    7.97512190394009    4.56655916037009
N    14.28941510361017   14.20795703539489    7.39111388846873
N    11.81441368392179    8.03917388716791    7.20598332607262
N    15.00105028589177    8.35090848777218    7.44588908132320
N    11.62945919620941   14.19171089493137    7.41999702260136
N    14.05723327136612   14.35728417921445    4.25898464984405
N    15.12519113667304    8.30538690121489    4.80255736509910
N    11.45669750095091   13.80628253422290    4.30226717976876
C    10.89751926365715    7.40938030761136    5.11573720670117
H    10.18140004936511    6.86253593798999    4.50559247321159
```

C	10.76102807369772	7.47623697239140	6.51254570896355
C	16.13259233903672	7.96813611075282	6.91918471760900
H	16.93967013637883	7.60223934144953	7.55126246582625
C	12.24017094940515	14.77784094261074	8.42035799660428
H	11.65714204042144	15.26525765048343	9.19997675002273
C	13.63372106744699	14.79105931691404	8.45679632037221
C	16.26211360555359	7.99147706612082	5.52019532891867
C	12.43604556703510	8.28941409697586	0.92389946805358
H	12.31846795215584	9.02563590111113	0.13758303776640
C	12.24443353442650	7.65251565886402	3.21892762010207
C	13.34263597743076	14.74130491367495	3.22981337072625
H	13.81369469501928	15.29377891368302	2.41845142402034
C	11.48286127630849	7.98680562626333	8.51440490748523
H	12.15188589083693	8.36101343822263	9.27445587743243
C	14.18280248035819	7.50716817524153	11.45705147834534
H	13.96018252848000	7.29005800280416	12.49537849215595
C	11.97858698430741	14.45407602260955	3.20061280423706
C	12.09469537360870	8.62776345768373	2.22801118385648
C	14.23513942605244	6.48175748926861	10.53168028015513
H	14.03874335920571	5.46423968950737	10.84972208530980
C	10.22338202385435	14.35029861655623	7.29114683913513
C	12.92519993467843	7.03227796481483	0.61468779094596
H	13.18551907226106	6.78771228470774	-0.40866002475967
C	15.59792457232588	14.35021925020023	7.63425389297484
H	16.33699590169176	13.99118012165722	6.93339315737012
C	15.39805713120071	14.81359130442043	4.37117055160117
C	9.38545754781748	13.25508261883651	7.52606377063694
C	16.81544478321879	7.92944568254924	3.36082180975865
H	17.35071898337661	7.82965470912216	2.42935061415830
C	11.55890270247964	9.99846025634010	2.55818250188769
H	11.62160344736086	10.12470368979873	3.64203787626757
C	10.23328697040211	7.39855942144411	8.67793705148876
H	9.72882632329698	7.23508609476720	9.61760310038084
C	13.09667948709703	6.09327780610845	1.61573992633852
H	13.50653211668665	5.12010586774913	1.37104495778857
C	17.74752716034744	14.37232155021036	4.35457509993288
H	18.57518739627882	13.68462721682961	4.22520815786461
C	15.81355103724989	15.01233972614919	8.85074236638893
H	16.77429871994542	15.25213544856456	9.27905238262590
C	15.63225305128595	16.15492768804905	4.70827516290191
C	14.51304698173268	6.73480659266261	9.19341770179871
C	12.77224979054564	6.38312523937769	2.93504298378689
C	14.56100287989000	15.29899617815578	9.37631378155782
H	14.33256440155312	15.81041327472050	10.29926113011860
C	8.01572554475162	13.43863491117767	7.37578804655741
H	7.34783165001219	12.60619461174842	7.56440895745520
C	14.76128261397858	8.06219195883578	8.81363180295418
C	8.34207427933567	15.71995425448969	6.73041840512978
H	7.92905228209087	16.66773385331868	6.40448141638228
C	15.47244806825654	8.26469302174498	3.49826487561660
H	14.74910108204096	8.46556721996719	2.72252267129096
C	13.02605601417914	5.35402557081775	4.01659788227919
H	12.90320907664429	5.84483594551754	4.98524389908284
C	13.20577225381803	4.79048363890687	8.25135953498601
H	13.09923174729252	4.26315854434778	9.20238812104438
H	13.20350500278227	4.03852787930241	7.45782600162282
H	12.33217950481225	5.43064788707642	8.12165135923467
C	18.00213243918229	15.69714649441544	4.65931706304079
H	19.02348006100747	16.04403850783605	4.76544779238161
C	16.94947869018673	16.57552086255555	4.83982527197115
H	17.15308009023428	17.60756020470161	5.10168875393438
C	10.61924725677867	16.75308630518935	6.52203776142254
H	11.65159016523197	16.39752565244781	6.53879716798829
C	14.50106834276526	5.59490728823405	8.19697123531123
H	14.56901209251604	6.02184605664129	7.19408832676819
C	7.49309998891621	14.65932989038146	6.98940523463671
H	6.42179924453804	14.78195694990346	6.87966927931593
C	16.44731351553145	13.90126007296051	4.21299622084668

C	16.19910478065720	12.44749409952458	3.89191167373875
H	15.13035571010114	12.25385180370222	4.00865452151969
C	9.77274304364525	7.06238768164347	7.40132354628275
H	8.83928199309981	6.58508269944242	7.14343786862052
C	10.97117074710392	14.68769675967711	2.25501357083419
H	11.09049455143952	15.17995209697853	1.30161538074510
C	14.39876022783099	8.81433357956310	11.05340033203538
H	14.33893591530751	9.61435338464473	11.78091414791529
C	12.00791261742396	4.21866712892218	3.94937827690851
H	10.98480978391682	4.58728146166578	4.04654318002892
H	12.18531373275377	3.49476151042104	4.74874641346687
H	12.08137050742156	3.69282418074713	2.99381181016915
C	9.80761929175711	14.16361896502165	2.80191839989852
H	8.82166409750899	14.16012096618156	2.36378112033994
C	10.15211765379105	13.63974604976032	4.05581426022956
H	9.50431895445975	13.16803064390679	4.77990554994928
C	15.71170293088505	4.68397085628775	8.38283518207079
H	16.64921591360784	5.23513108419833	8.28413866204416
H	15.70744127370205	3.88412993910899	7.63797305429992
H	15.70189838117875	4.22310107525901	9.37402335603034
C	9.71795246761856	15.58767582459621	6.86573602093420
C	17.32107761301664	7.74413987654730	4.65092323522223
H	18.32789328517310	7.47200024104934	4.93013270203316
C	14.68573606114770	9.11886307295446	9.72895231765339
C	10.35359630134950	17.28247771801244	5.11639406041784
H	10.41983872425503	16.48674380440331	4.37296454410296
H	11.08627433952858	18.05330896326261	4.86291479589538
H	9.36183417854588	17.73452759050139	5.03954599240671
C	12.39134006273800	11.10889131504348	1.93300265722632
H	12.34757026628627	11.08945716701735	0.84072916723838
H	13.43786518010862	11.03114561714223	2.23640875919581
H	12.01866222584893	12.07922891909513	2.26054340793834
C	14.50782412298339	17.13475672476197	4.96252461885333
H	13.56706464516836	16.58115521346984	4.95098886710617
C	14.93522928610759	10.53661248283120	9.28167798568572
H	14.68304480997357	10.59205880502439	8.21843494662645
C	9.92915643630208	11.90753381009191	7.93398983857131
H	11.01622383781170	11.93684493591515	7.83003512809668
C	10.50304990170699	17.86904785554690	7.55629695033121
H	9.48393093632810	18.26296188229511	7.58884195184562
H	11.17539196685527	18.69461932020632	7.30950592541076
H	10.75267353640011	17.51692514361794	8.55956103608738
C	14.44883944169221	4.80520729357639	3.97383738966741
H	14.62418210226510	4.21358596331610	3.07209147374116
H	14.61985743633771	4.15003912513532	4.83220248407510
H	15.18742930488493	5.60747714028339	4.00004917124093
C	16.57015397687875	12.13393482399198	2.44573522725843
H	16.00110951772300	12.75154993374268	1.74647553503359
H	16.36430415765764	11.08384719451402	2.22410516221661
H	17.63333651021290	12.31491516693809	2.26413126488152
C	10.09023092026050	10.11500032957968	2.15960129065589
H	9.70375804555107	11.10131842738413	2.42677487415646
H	9.48198468865250	9.35854217381991	2.66157346289467
H	9.96643674493827	9.98157605615319	1.08093926101626
C	14.62191412806999	17.78776540332732	6.33617714066259
H	15.51380233079075	18.41442272540278	6.41080261463228
H	14.66963065821104	17.03894314356102	7.12798183558576
H	13.75415986786803	18.42738966920102	6.51871012804276
C	16.92171963589391	11.52211589190441	4.86252047048206
H	18.00728955174347	11.63599772732568	4.80180908929405
H	16.68579980946507	10.48312484169324	4.63415234890755
H	16.61022108899185	11.71432585408282	5.89144919540641
C	9.42984479598101	10.79847673284567	7.01714932066908
H	8.34069938679120	10.70881312831963	7.04175074600106
H	9.84687728540379	9.84124566027350	7.32780303914485
H	9.74073835798689	10.97697505163169	5.98546038994050
C	9.61448985372329	11.60436099840608	9.39579448250082
H	10.02559364212726	12.37050760870578	10.05778203400116

H	10.04235469452192	10.63926240790252	9.67764635846717
H	8.53536720164685	11.55795303356445	9.56716120888765
C	14.42868849904479	18.18668887214709	3.85992407666171
H	13.59377215448100	18.86890664697540	4.03902168021388
H	14.28939049694837	17.72939399928796	2.87788898150360
H	15.34571346696251	18.78023270128194	3.82033550181555
C	14.05701186758873	11.55071064422431	9.99696091510987
H	13.00015116111179	11.28692288888362	9.90981298991683
H	14.19687877204392	12.53503107198764	9.55103115097929
H	14.30362255958966	11.63639166554583	11.05884956593418
C	16.41328901418397	10.89350846820962	9.41820332740167
H	16.73173923806580	10.83955574513333	10.46329168937002
H	16.58911266986117	11.91030843483298	9.05913502286255
H	17.04177097513228	10.21278984397379	8.83938615364313

[(pyrNdipp)$_2$Nb=P=Nb(pyrNdipp)$_2$] (S = 3/2)

Eh = -3532.898349494054

Nb	13.39565424474980	8.82813077621624	5.98196505004611
Nb	12.93760214986890	13.41488167887577	5.86450425110662
P	13.14001721956892	11.12743416632714	5.88993125952964
N	11.93757954836769	7.95745563713509	4.57235978651501
N	14.30102949195459	14.17964926634561	7.41691117845152
N	11.81541571184769	8.04618552452829	7.21377519015216
N	14.99797655587961	8.33789511723838	7.43517580002674
N	11.63322520502447	14.21001755778118	7.40894899432014
N	14.04951194192213	14.37948339796035	4.26893787973798
N	15.11210605078892	8.32303880997818	4.78709848044858
N	11.45705190545078	13.77373077501564	4.27899091480207
C	10.90133276256752	7.39374677910883	5.12949821955757
H	10.18359910983298	6.84178666929767	4.52540866684866
C	10.76577464410473	7.47157122940007	6.52565877219337
C	16.12314237913050	7.95732766088993	6.89718768197520
H	16.93421509791725	7.58513493388857	7.52103022623184
C	12.24438740712503	14.78072618212327	8.41921642787106
H	11.66079990582740	15.27151832914738	9.19645779977256
C	13.63698459692395	14.77391060158715	8.47173648777880
C	16.24797753607109	7.99435164509906	5.49837457270295
C	12.40173627103326	8.25874945888211	0.92325324080977
H	12.26668804632048	8.98838926633925	0.13352211494950
C	12.23742650571464	7.63390388258981	3.22356150786219
C	13.33718898164228	14.74870171435711	3.23042212041404
H	13.80786383476901	15.30592795271856	2.42198120152300
C	11.48471653597410	8.00326003018631	8.52124688029293
H	12.15230498042662	8.38676255233224	9.27806463579103
C	14.20444602398250	7.48583427325443	11.44950677729144
H	13.98863480671257	7.26648876531148	12.48879354451826
C	11.97997106405218	14.43861315398686	3.18727322263385
C	12.06600474807005	8.60073659567041	2.22804089971794
C	14.24620772987175	6.46316963299911	10.52068864637660
H	14.04854224034928	5.44534277507459	10.83692908547898
C	10.22786807192142	14.37149664219726	7.27482624688058
C	12.90599216843943	7.00705915889060	0.61735227776529
H	13.16096755764893	6.75972570271549	-0.40667459753182
C	15.60614909076999	14.32191848071370	7.67106318576612
H	16.35112750661058	13.95723010888113	6.97912356906388
C	15.38942895508350	14.83663785810417	4.38643606175906
C	9.38734952252081	13.27888030818486	7.51388832313373
C	16.79919795380574	7.95934528399990	3.33845576340623
H	17.33213228690301	7.86915042909751	2.40469978221361
C	11.51638235597072	9.96795171773982	2.55030115388449
H	11.55715063411999	10.09233643116152	3.63510526416163
C	10.23781351488308	7.40858138479451	8.69091762997069
H	9.73525702385522	7.24934565014410	9.63231272126257
C	13.09844944835008	6.07672702886623	1.62275022144222
H	13.51860345710658	5.10736393646563	1.38047657484324
C	17.73884770482267	14.39281296640235	4.37851401221384

H	18.56631181309942	13.70430202479107	4.25167796947963
C	15.81349578605244	14.99399865359098	8.88510818296473
H	16.77102872917556	15.23503071234394	9.31993249004967
C	15.62417075394710	16.17692827050531	4.72590387322007
C	14.51573943678139	6.71931752569852	9.18129981176437
C	12.78004775101904	6.37012728526364	2.94259194933713
C	14.55784018838868	15.28648200718916	9.39705608941347
H	14.32198725139334	15.80601343909691	10.31361799626229
C	8.01832910243938	13.46462709315761	7.36071955093616
H	7.34816324105617	12.63500232178319	7.55332616678507
C	14.76651139788559	8.04668259650999	8.80359639627625
C	8.35050188890107	15.74132988978302	6.70203808486048
H	7.94021335497514	16.68793245643091	6.36926593327399
C	15.45685061208238	8.29873289659490	3.48344078606688
H	14.73293568246750	8.51303855204106	2.71164280626978
C	13.05162434437371	5.34953740095244	4.02770825311429
H	12.93282994085970	5.84481398814433	4.99453808979795
C	13.19352803956400	4.78492011072837	8.24138485170217
H	13.08869330647886	4.25701649409841	9.19226828295233
H	13.18341610000699	4.03405623469726	7.44691825452812
H	12.32284224736907	5.43006927051067	8.11667566493693
C	17.99372176820005	15.71698181408090	4.68576400836088
H	19.01501287850427	16.06271652636597	4.79615923114943
C	16.94126726553108	16.59617794637008	4.86317744128908
H	17.14486698509406	17.62772312774865	5.12715603483017
C	10.63132666398167	16.76668341198162	6.49135151245650
H	11.66240134872264	16.40769622392159	6.51514900841479
C	14.49281117140586	5.58242805931595	8.18186139498185
H	14.55714875086573	6.01182453301177	7.17988653379294
C	7.49892536231721	14.68426868422459	6.96644279920155
H	6.42804366817263	14.80845268203519	6.85432187943230
C	16.43871791767486	13.92331085875754	4.23165012697542
C	16.19175195891396	12.46893881255286	3.91108808927421
H	15.12036456340264	12.27809931802977	4.00761264632420
C	9.77923038374403	7.05929286597828	7.41803154839791
H	8.84893254688280	6.57366272811695	7.16422948735850
C	10.97542492864756	14.67123876838343	2.23556358468338
H	11.09634849333014	15.17372713269988	1.28771906152501
C	14.42234935970297	8.79334415377822	11.04805668071251
H	14.37046715358790	9.59128917591031	11.77837759809789
C	12.04214817030723	4.20567061313230	3.97487948159266
H	11.01703106735211	4.56631959520634	4.08080613756876
H	12.23333082030289	3.48632822597171	4.77514502733112
H	12.11038729674087	3.67694760021840	3.02054756520671
C	9.81475743959005	14.13198774117477	2.76965119342758
H	8.83162553728238	14.12286127222538	2.32521730522631
C	10.15768329227625	13.59788787645181	4.02195661924978
H	9.50978738968160	13.11184388569826	4.73663536881907
C	15.69982951322135	4.66491652953408	8.35885832182141
H	16.63968836931277	5.21144658460533	8.25612203221119
H	15.68732658514911	3.86667792976999	7.61239621490814
H	15.69349631501155	4.20187773539457	9.34904885375686
C	9.72587225104064	15.60660860618491	6.84053320676195
C	17.30558259007468	7.75441764370129	4.62430730621821
H	18.31133315347918	7.47333240083329	4.89849284835600
C	14.70129541146679	9.10105054609178	9.72265522262494
C	10.37177858878166	17.28461654149280	5.08027961979100
H	10.43646512374852	16.48173214661569	4.34431738605339
H	11.10846487267757	18.04983998905138	4.82140832999264
H	9.38208473715325	17.74000996445441	4.99691560033782
C	12.35920423410129	11.08153618479280	1.94379354534100
H	12.35468417213811	11.04984473193552	0.85101286033959
H	13.39479121058006	11.01732194914982	2.28551421366517
H	11.96559595300137	12.05155847576105	2.24729763944988
C	14.49974911075414	17.15681694661894	4.97790907084141
H	13.55743178058976	16.60664496513741	4.94602340129946
C	14.95173238536955	10.51966067580344	9.27827879826682
H	14.68475258678247	10.58034060792127	8.21884298133996

C	9.92852233855491	11.93325768197661	7.93234493749162
H	11.01373767794558	11.95279165512047	7.80825514159179
C	10.51494447792635	17.89070288482493	7.51669498198878
H	9.49635459946207	18.28632479736762	7.54465687236514
H	11.18875733626007	18.71351616440415	7.26480672504098
H	10.76260380677437	17.54549525906676	8.52287654448051
C	14.47843044121413	4.81249824533333	3.97585380854275
H	14.65211114238124	4.21960589171111	3.07464314035517
H	14.66137569949717	4.16121812306982	4.83471608807706
H	15.21033694023306	5.62109053005857	3.99437001172610
C	16.58941686189515	12.14801894330315	2.47365317117035
H	16.03719190130111	12.76600058327382	1.76140505690245
H	16.38299349271854	11.09815525890563	2.25188937125041
H	17.65678727182928	12.32332657657899	2.31229091030135
C	10.05615860435056	10.08085506137998	2.12137756409285
H	9.66103649614305	11.06420994441475	2.38677819206020
H	9.44046830830014	9.31916954765203	2.60614932746254
H	9.95388761043073	9.95418194095303	1.03966983224714
C	14.60030331270871	17.78911462543669	6.36234423139052
H	15.49499658747295	18.40914035214757	6.45650744668972
H	14.63560871204451	17.02839472729188	7.14365262834752
H	13.73407511466156	18.43078351738522	6.54474914247534
C	16.89278156476431	11.54572155263499	4.89974149547558
H	17.97915480263198	11.66276557451218	4.86512627781376
H	16.66433147652109	10.50594950159124	4.66729658670211
H	16.55569234742079	11.73816743165807	5.92059779208205
C	9.40663310138507	10.81104157371763	7.04464834597145
H	8.31834248707714	10.72242028872700	7.09559566158920
H	9.83017910288250	9.85818453132607	7.36045980940749
H	9.69336591147569	10.97207033803465	6.00314903428942
C	9.63968251984147	11.65692201368732	9.40486391235912
H	10.07044303879421	12.42997924957880	10.04603190492221
H	10.06422297218870	10.69279659435608	9.69515300060831
H	8.56358604544593	11.62418060655980	9.59739966512915
C	14.43768870864915	18.22346213727665	3.88861734441950
H	13.60347538073412	18.90678319205821	4.06687972817627
H	14.30752370235925	17.77856428519747	2.89958777779733
H	15.35716101056071	18.81406487303971	3.86661367128474
C	14.08823107532549	11.53456606489955	10.01037751605844
H	13.02905061753056	11.27603960437198	9.93643335197870
H	14.22640713820057	12.52065435007870	9.56779736036042
H	14.34965078788254	11.61409138324817	11.06916359196823
C	16.43319406249757	10.86902349214143	9.39639250226012
H	16.76560551517208	10.80818008733604	10.43671884956331
H	16.60879887943991	11.88695288638054	9.04043827835927
H	17.05086043848926	10.18895719381922	8.80528541035902

[(pyrNdipp)$_2$Ta=P=Ta(pyrNdipp)$_2$] (S = 1/2)

Eh = -3532.967803697311

Ta	13.41207666744359	8.82194185161469	5.98731034265345
Ta	12.93003297810245	13.46997022856143	5.85207310502084
P	13.20509883107464	11.09174748947745	5.90051312429857
N	11.97614472713486	7.81479484375952	4.69979000663856
N	14.18170955889756	14.29762458930793	7.38809872456275
N	11.88256299598717	8.06325721630719	7.31211263030946
N	15.01276703728375	8.20055493105521	7.32593957243228
N	11.60748386420140	14.38845311555816	7.25391637555348
N	14.05012897395357	14.54282008840987	4.38941193019010
N	15.06814226519041	8.30033148394388	4.70413267017253
N	11.54729546075622	13.92755939080797	4.27459316096451
C	10.93684613439825	7.28748700665791	5.29914156454531
H	10.19445366390782	6.73969364179001	4.72279168725610
C	10.82978855108288	7.42502540662887	6.68420304444610
C	16.13920647092191	7.85424963462815	6.75259910201162
H	16.97231306145500	7.49938024179261	7.35570856265722
C	12.13192621658431	14.86320148886906	8.37457866734438

H	11.49047245148654	15.25867819241632	9.15599813985335
C	13.52052133567548	14.82733183037110	8.48406101732529
C	16.22478321650848	7.92718520096565	5.36095528946018
C	12.21778182449294	8.15000312986911	1.02871404596822
H	12.01552401763553	8.87999139235797	0.25367310151172
C	12.20774417211419	7.50973689681348	3.32888780379962
C	13.44902229612310	14.83050855827149	3.24341796262048
H	14.00477803854027	15.29595527292371	2.43557740672359
C	11.57863115049258	8.09458371076742	8.62177471394356
H	12.25165012774215	8.53826008768969	9.34007292257112
C	14.39007556977973	7.39938053979474	11.38554355486816
H	14.22012682851947	7.19042205608701	12.43535342985501
C	12.09665832562946	14.50917237058826	3.14377305637492
C	11.94733060409901	8.47717371488813	2.35320836397510
C	14.34460590250785	6.37340695564895	10.45891945527819
H	14.12772304894756	5.36390913761469	10.78885109887590
C	10.19970734225470	14.49473765811550	7.08409338195571
C	12.74185970255564	6.91686731112721	0.68610516493030
H	12.94491489245167	6.68288523593963	-0.35250188383328
C	15.50060257060375	14.32724289411228	7.68865910692831
H	16.24136836465503	13.97874956773607	6.98576569995330
C	15.40433996947884	14.94991513151709	4.54465434982182
C	9.38090374429433	13.39749473584713	7.37373342455268
C	16.70388356529503	7.97929417873006	3.18586677390634
H	17.20061560477391	7.92293405530160	2.22997373346740
C	11.38985296774843	9.83545659068553	2.70243581497113
H	11.41854252210506	9.94142049095156	3.78927028561703
C	10.34381028469336	7.48697095001110	8.85708548957430
H	9.86683232892208	7.37775634320250	9.81845046741249
C	13.01866649229215	5.98663626216223	1.67225718517072
H	13.45024120280775	5.03052278363127	1.39978199499104
C	17.74680295306563	14.47571975051789	4.47921984837149
H	18.56390780607180	13.78843622628709	4.29166929931262
C	15.70013772365571	14.86393718524344	8.95747638336211
H	16.65589107216295	15.00528273909877	9.43844970203335
C	15.66109830541237	16.25574051306993	4.98279853864739
C	14.55839420703630	6.61707150286042	9.10861761694100
C	12.76698802175717	6.26406083754571	3.00924632606070
C	14.44290337075632	15.18843598912103	9.46973717830302
H	14.21533589481487	15.62739142314558	10.42916946825158
C	8.01061041836407	13.53891446817028	7.18276261272838
H	7.35579194850007	12.70479978607030	7.40679968073322
C	14.83639612776301	7.93373337136070	8.71283451772389
C	8.30167729561041	15.78510589455167	6.40716577279437
H	7.87619075981814	16.70332418446014	6.01888881395884
C	15.36797011570286	8.32894316090491	3.39397590865583
H	14.62220218879938	8.58748001619966	2.65707567006272
C	13.11561455616617	5.24550215584882	4.07210490790689
H	13.03855726367789	5.73642730712504	5.04520770521150
C	13.13699197385720	4.73278276298642	8.22056105389242
H	13.03354088370539	4.23049840710858	9.18520708523168
H	13.07906654189447	3.96632727388733	7.44322128640954
H	12.28904602820843	5.40866166062104	8.10058221945262
C	18.02230585130507	15.76617056888237	4.89160172084880
H	19.04934801559552	16.08645253586750	5.02412064661638
C	16.98373399574267	16.64475805920867	5.14482786322399
H	17.20379429021785	17.64974730838524	5.48573808929231
C	10.57114244218407	16.85422825353454	6.22072416452309
H	11.59905136162179	16.48773775613125	6.24350647809659
C	14.46327677917226	5.47876041111955	8.11617425328836
H	14.51308376666707	5.90169545762710	7.11067757027480
C	7.47097819564631	14.72065164153689	6.70861868066599
H	6.40022371446529	14.80961882585264	6.56546442206972
C	16.43788955668529	14.03804070444388	4.30413059588587
C	16.17323723385963	12.61620623728656	3.86805934198475
H	15.09998392399238	12.43011441965821	3.95496392366023
C	9.86611357587526	7.05208606298942	7.62409907396682
H	8.94219077890396	6.53183126431440	7.42128843732902

C	11.12753568396690	14.61566787116968	2.14289238337610
H	11.26965659235304	15.02998561556742	1.15644405942221
C	14.64347975193222	8.69343153042400	10.96852486179636
H	14.66974023716325	9.49500998412977	11.69737182693144
C	12.12623302621145	4.08293417093032	4.06598045162317
H	11.10056949034134	4.42380186673876	4.22239223683800
H	12.36984786171822	3.36642872050630	4.85443472034761
H	12.15711346693671	3.55671398736764	3.10843866838833
C	9.95681947253756	14.07929716262405	2.68219508293087
H	8.99558540249675	13.99614853382473	2.19857087084601
C	10.25083665624151	13.67382614745123	3.98075711033472
H	9.58966202166899	13.23002039264684	4.70902695216756
C	15.64211351605344	4.52020818344482	8.26285105840358
H	16.59690795517354	5.03408859602727	8.13103216590057
H	15.58073567440213	3.72050775481373	7.52052076230999
H	15.64895722901369	4.06039097929135	9.25440067825320
C	9.67591918200470	15.69255661152576	6.58154968089285
C	17.25176322179962	7.71497509852900	4.43808342399676
H	18.26206409352725	7.40905846100924	4.66418063457444
C	14.86454067290883	8.99116969048760	9.62820778116836
C	10.30520089255944	17.36860879373080	4.81072697101903
H	10.36555261248100	16.56278351497172	4.07791614584939
H	11.04450254822355	18.12869849407114	4.54543626200653
H	9.31734604951612	17.82953415093469	4.72967774350240
C	12.24067048703526	10.96198708383822	2.12873644842346
H	12.25279872269599	10.94629993426265	1.03580221858002
H	13.26997890323416	10.89544452191867	2.48786904057641
H	11.84057381267307	11.92724632634350	2.44065031954319
C	14.53854838430269	17.22026459821154	5.28494165119915
H	13.61438139720857	16.63955886153324	5.31576418696333
C	15.13348976074408	10.41477544028478	9.20390578751564
H	15.11710424915427	10.45134224152456	8.11245634456286
C	9.94349036371495	12.09246995452357	7.88587775424982
H	11.02800037774523	12.11714364126083	7.75119921360858
C	10.45429223838970	17.97716000674667	7.24743631886022
H	9.43315852872040	18.36670674135492	7.28349943469686
H	11.12141397275051	18.80405653419362	6.99022454870884
H	10.71491877518920	17.63151610434359	8.25015944445455
C	14.54790846109363	4.73869020078468	3.94198931386224
H	14.68731570945758	4.15746846372252	3.02740332553203
H	14.78837236486238	4.08400487525758	4.78378968908206
H	15.26317978111107	5.56235853357437	3.93097255849952
C	16.55901068671291	12.40709806197663	2.40676229578106
H	16.01134135540786	13.08777294756017	1.75087563767458
H	16.33753124949064	11.38187467929409	2.09879588432020
H	17.62760935964019	12.58227588361351	2.25378938042853
C	9.93439924750232	9.96104645608749	2.26191848424951
H	9.54274180567598	10.94098621440879	2.54384311401039
H	9.31051289064674	9.19213430695128	2.72463843353990
H	9.84050959671329	9.85887372500400	1.17709927433565
C	14.69964362731282	17.88586486130822	6.64654960279354
H	15.56495987863114	18.55341033313312	6.66978305921399
H	14.81930692859119	17.14475172587530	7.43781622753352
H	13.81643806406806	18.48897085531471	6.87253569835570
C	16.87088124595599	11.60633456554924	4.77162315087774
H	17.95852665554853	11.70593738309974	4.72487140928764
H	16.61679061613817	10.59052271877674	4.46682316862576
H	16.55737140404814	11.72895535343484	5.81040548326698
C	9.43231028672759	10.89492270362880	7.09505102092096
H	8.35138683845754	10.77050063178790	7.19877827210962
H	9.90607049717864	9.98033837205545	7.45324682240850
H	9.66765131629409	10.99792462046346	6.03360983198125
C	9.67474419956332	11.92760421125152	9.37861780878355
H	10.10554685060707	12.75251757114941	9.95093646661194
H	10.11252211017067	10.99418924046532	9.74156958470138
H	8.60090578242421	11.90160782056757	9.58434762468375
C	14.39782321398747	18.26218410395109	4.17859523027073
H	13.57009560379281	18.94208453897819	4.39637685209997

```
H    14.20673924410158   17.79517947645727    3.21012233955102
H    15.30999508708407   18.85862371947335    4.08858817204304
C    14.04972358301023   11.36803596608956    9.69271312799729
H    13.07250146677928   11.08449199763306    9.29613633868335
H    14.26541163422481   12.38217412058888    9.35475065437543
H    13.98876232420122   11.38940620894303   10.78394033811497
C    16.51621662805895   10.86890530266259    9.66300193129587
H    16.59373861180925   10.85588498828271   10.75379589350650
H    16.70399840322267   11.88996663097841    9.32333376428431
H    17.30106324556486   10.22068032897201    9.26518401422885
```

[(pyrNdipp)$_2$Ta=P=Ta(pyrNdipp)$_2$] (S = 3/2)

```
Eh = -3532.967301645271
Ta    13.38806793781128    8.83575780742462    5.99154967767507
Ta    12.92731640880640   13.42800338658899    5.83632775691604
P    13.04107849069790   11.13891905801556    5.75894771878731
N    11.96984679973976    7.98315913601309    4.61354095627917
N    14.26519002276036   14.19344441440683    7.38430216347327
N    11.82728176094901    8.04950294322068    7.21505095555055
N    14.98441812291051    8.27910545797683    7.42009400605887
N    11.61312780568237   14.19299713086997    7.38918705946902
N    14.06270485017457   14.38825900290372    4.25708339532362
N    15.10115710642617    8.33132743486901    4.78541157310646
N    11.47606901486742   13.85331201303244    4.27201101122832
C    10.91107512694758    7.39932226878228    5.14232574496997
H    10.20830493186073    6.86088460640432    4.51377457574606
C    10.77315485968654    7.46294254352063    6.53603113626163
C    16.09847889662730    7.86596658488924    6.87316901771330
H    16.89703478124374    7.46130622182613    7.49215809090324
C    12.21490914731858   14.78400518660679    8.39563872908065
H    11.62452299779603   15.27631461210657    9.16569408283662
C    13.60718788671831   14.78917842255433    8.44466983302300
C    16.22517961564393    7.93206921990027    5.47939533288403
C    12.39608875056218    8.28386117644841    0.95226965302950
H    12.23242183047801    9.00501020014551    0.15997852998062
C    12.27167010685936    7.67025527229339    3.25871611066854
C    13.36037683785657   14.80047488633043    3.22270035217632
H    13.84617497600273   15.36394923303742    2.42886439598404
C    11.50292967778892    8.01643551246647    8.53177896220908
H    12.17279784518060    8.40768868272280    9.28171107706635
C    14.23735533081445    7.49389402705802   11.45803279495578
H    14.03476642208317    7.29344432462920   12.50372700116071
C    11.99924744578581   14.52027356693958    3.17873355234408
C    12.05973946573536    8.62399867143414    2.25831645528729
C    14.27251450416401    6.45458191148723   10.54855606824995
H    14.08196227068729    5.44215508784933   10.88588506668833
C    10.20457002650582   14.34990887366880    7.25524182566872
C    12.93171774063895    7.04567033720164    0.64733048856230
H    13.18637065506923    6.80104172545860   -0.37747144280967
C    15.57546015835570   14.32441811795496    7.63800180854991
H    16.31630626099237   13.95791769473909    6.94333769756028
C    15.40750181805961   14.83449765563192    4.38582252416998
C     9.36809812520258   13.25308150203278    7.48722698953664
C    16.77780675306922    7.98084134446975    3.32094867763549
H    17.30721188746639    7.91666318782071    2.38316883492283
C    11.46667265688330    9.97715431070853    2.56512999586328
H    11.47033962032123   10.10067108209266    3.65009300375318
C    10.26317844672953    7.41789192435436    8.70915465088494
H     9.76638728108514    7.26261859379273    9.65446782360474
C    13.14999782369520    6.12300435021412    1.65484693175946
H    13.58630526945016    5.16069216298128    1.41291686249873
C    17.75401245809141   14.37541553982426    4.39851339100388
H    18.57758993761893   13.68090947032819    4.27954130660949
C    15.78683473966931   14.98642271660494    8.85363417039857
H    16.74659029753405   15.21708726969473    9.28900534653496
C    15.64922221672132   16.17457184050150    4.72366507742965
```

C	14.52471127826559	6.68597182579269	9.20092402821247
C	12.83252951243151	6.41458707073909	2.97489751748439
C	14.53340830738135	15.29057243827333	9.36792335222397
H	14.30395606282495	15.81269016696476	10.28453371074012
C	7.99855724934202	13.43668555929788	7.33543625456788
H	7.33092407680907	12.60382127515460	7.52185308687771
C	14.76537885399951	8.00642179576444	8.79621544174758
C	8.32397131366405	15.71974984313717	6.69506447819441
H	7.91071642221197	16.66809561230228	6.37118812244221
C	15.44515958542288	8.35493551444429	3.48272054192554
H	14.72766117039207	8.62543445440477	2.72269426431129
C	13.11078131429559	5.39460685196691	4.05862951094220
H	12.99517247031489	5.89178317851769	5.02538322155103
C	13.16955537429868	4.76319890660782	8.29389711068140
H	13.04548366161877	4.26407589404579	9.25794865667578
H	13.14789100999364	3.99259601806489	7.51879091253035
H	12.31550827536617	5.42543650143973	8.14530102597494
C	18.01606337953330	15.69840186876475	4.70460038696505
H	19.03878111880679	16.03728331554304	4.82244021644009
C	16.96814688850842	16.58481000899840	4.87137380212354
H	17.17636780773306	17.61582717665618	5.13385319897340
C	10.59988003779427	16.75707683066058	6.49600483444732
H	11.63295004413224	16.40269662571302	6.50572861871987
C	14.48753051408797	5.52910869629627	8.22534965938485
H	14.56562961030482	5.93403430178878	7.21469365831175
C	7.47573349241237	14.65801330877554	6.95095406247212
H	6.40447665244536	14.78012979875702	6.84034569323270
C	16.45204798493571	13.91405274707873	4.24194485164557
C	16.20159775921761	12.45961061158289	3.92386013457715
H	15.12773122950108	12.27434813997520	4.00256697265266
C	9.79710495037126	7.05343619900678	7.44044751949605
H	8.86813094401900	6.55959726600807	7.19858088083477
C	10.99279798856429	14.75316898656910	2.22960765222607
H	11.11119139098757	15.25838853867391	1.28305733111190
C	14.44200130345304	8.79512513013248	11.02902079639972
H	14.39153198406870	9.60625197356631	11.74450755958329
C	12.09988953537372	4.25151056305557	4.01042904686979
H	11.07638247592170	4.61384412455728	4.12209860030072
H	12.29442176629728	3.53263740812283	4.81039437397726
H	12.16427387018166	3.72236103651295	3.05594328086436
C	9.83407613665089	14.20817957045620	2.76315934349653
H	8.85149353283532	14.19599519751694	2.31764979396396
C	10.17441456635331	13.67363484820274	4.01423403127337
H	9.52936248112270	13.18041519602935	4.72620571262773
C	15.67351392067772	4.59052449945621	8.43035568221736
H	16.62594161393673	5.11525157579466	8.32711970506235
H	15.65086387843603	3.77949943801302	7.69808806213324
H	15.64929720289410	4.14456939401512	9.42800046037037
C	9.69990029390054	15.58803804557935	6.83194043032942
C	17.27587826784698	7.69784515536657	4.59321676425447
H	18.26999947304406	7.36597365220678	4.85245827020389
C	14.70256793229267	9.07884565868857	9.69510291913804
C	10.33011709662578	17.30165399383419	5.09711383243657
H	10.38991024904875	16.51363769811610	4.34511935782732
H	11.06446021620317	18.07247257294430	4.84844190113914
H	9.33984715344265	17.75838631160268	5.02950032693143
C	12.30494976987946	11.10934875073917	1.98593455347706
H	12.35713547334932	11.06249833059389	0.89485635252783
H	13.32239322630056	11.08098478684350	2.38242246028958
H	11.86798457632801	12.07132553891827	2.25484068161419
C	14.53227485588961	17.16632094516961	4.96590343283635
H	13.58318184627745	16.62905258416179	4.91955410885762
C	14.93083033673092	10.49216117284048	9.22012865816215
H	14.61047983563018	10.54493119524840	8.17364770375865
C	9.91325675595093	11.90787015026140	7.90085258362074
H	10.99528084177048	11.92157794759711	7.75066663224969
C	10.48661897708399	17.86297515318489	7.54159288676090
H	9.46721648564340	18.25542342177354	7.58050743840288

H	11.15763667911463	18.69138592135054	7.30077460535654
H	10.73898296985852	17.50195118680949	8.54096069166226
C	14.53518372263999	4.85298738981184	3.99989503108542
H	14.70009769028772	4.25319305065531	3.10148854314754
H	14.72192157878390	4.20621964460318	4.86149713881101
H	15.27078549739507	5.65809338282607	4.00753185399481
C	16.62269298135621	12.13029299873950	2.49506645678237
H	16.08712186418458	12.74954832128532	1.77144041630466
H	16.41192502203543	11.08140745172857	2.27345051373347
H	17.69398583992902	12.29687657398852	2.35126711833719
C	10.01949546665542	10.06063427621743	2.08846123335091
H	9.59569203929134	11.03326721093473	2.35034799397013
H	9.40576386961659	9.28244347265513	2.54857856335238
H	9.95149469523794	9.94258900052582	1.00301648208833
C	14.62240344334644	17.79199063709757	6.35418580445873
H	15.52004806417597	18.40583973056694	6.46005536687630
H	14.64543187457944	17.02809378441629	7.13271175226291
H	13.75813520059691	18.43801813498147	6.53049713510659
C	16.88117568181034	11.53907697790778	4.93052686762559
H	17.96782276159198	11.65747942100491	4.92081476326739
H	16.65926667809081	10.49837356767923	4.69589440974607
H	16.51967238865906	11.73365848365871	5.94250178307343
C	9.36708424870604	10.78034060293510	7.03551383002057
H	8.28106163179728	10.68879831423140	7.11939775900178
H	9.80119886336146	9.83006891600510	7.34477079529387
H	9.62284765326075	10.93563389512811	5.98505417533031
C	9.65862798110516	11.64514963844241	9.38234330902966
H	10.10973983067860	12.42021883773945	10.00719252318947
H	10.08370005295904	10.68020990885410	9.66887400027970
H	8.58721712774400	11.62144971702604	9.60101911474732
C	14.49690690931134	18.23942567754795	3.88162512962818
H	13.66881247047186	18.93185429534482	4.05296319781175
H	14.37450704307681	17.80192557003320	2.88836790605225
H	15.42370721694397	18.81870815213605	3.87498462717662
C	14.10795497306966	11.51968092779183	9.98070716305273
H	13.04623582910828	11.26179352061347	9.96954056194940
H	14.22303101488937	12.49815783316139	9.51490146536078
H	14.42740906431530	11.61691551896233	11.02187046364169
C	16.41676560769845	10.84062433038382	9.25928416756595
H	16.80196969686177	10.77960636476684	10.28120426410173
H	16.57535023935212	11.85851722498378	8.89551832085930
H	17.00361267379120	10.16117509076633	8.63708545163076

[(pyrNMe)$_2$V=P=V(pyrNMe)$_2$] (S = 1/2)

Eh = -4678.011515633115

V	13.39159062385484	8.98743512742121	5.99748337269607
V	12.92262651421188	13.42054257427399	5.85498246960841
P	13.16482160157306	11.09558185242691	5.92929214298233
N	12.26360876253779	8.15540832240279	4.43468558214340
N	14.48829763326753	13.79038796238444	7.18628991401423
N	11.70476132965584	8.36852436499340	6.99713360732773
N	14.65899308800354	8.50588562445831	7.60082890742655
N	11.88692934441844	14.14180109126403	7.45169598231026
N	13.80915322627028	14.23700185456001	4.21322028939029
N	15.17781076916848	8.67968401454054	5.02715112308682
N	11.32359066781575	13.39804749964182	4.51120859211789
C	11.10330085044925	7.70489421512661	4.81630877289388
H	10.42529618569094	7.24733792397731	4.09654956003236
C	10.75171889237297	7.82220171418448	6.16703014021749
C	15.88942394695257	8.28164229178642	7.23894552251620
H	16.64236500405523	8.01481936515896	7.97996526222086
C	12.63786971689862	14.44314143585619	8.48676716366810
H	12.17832648463837	14.80474197862153	9.40562518791674
C	14.01807961142482	14.28719125378903	8.38341057406752
C	16.21788488689380	8.39067749063578	5.88169173738858
C	12.85848187682819	8.86870050342083	0.86550987602332

H	12.66561026398028	9.65667522051387	0.14553359439393
C	12.65340472087334	7.99829296881078	3.08237866292945
C	13.02120844123602	14.32007389830315	3.16525118209463
H	13.40760692531127	14.70727154144152	2.22341375908038
C	11.15662877736781	8.41838986068629	8.22080409142658
H	11.70181879938860	8.81573882622553	9.06351883001595
C	13.45064927427934	8.04184347034458	11.57354830433059
H	13.10944739058956	7.92593807971701	12.59565810036701
C	11.69758171949994	13.90283466609119	3.28403010497936
C	12.38550038150557	9.01831535522455	2.16522860310456
C	13.45225548428583	6.95490382895414	10.71547069693041
H	13.10795297583139	5.98706879569376	11.06293292652506
C	10.47505745567206	14.19722980235802	7.54691187396221
C	13.55967766662546	7.73764405988900	0.48442799246365
H	13.92222896197106	7.64027274491298	-0.53226779832936
C	15.81186140930205	13.70805550035943	7.30459409770717
H	16.42314220887696	13.34546011988568	6.48985627756569
C	15.18412100120067	14.55793256926757	4.10347839096298
C	9.78943547163384	13.14253765603312	8.15736194397151
C	17.08748422391020	8.53542311052227	3.83745517907064
H	17.75573806993598	8.54292457319711	2.99007093056570
C	9.85747660904340	7.90439668185651	8.20394201468753
H	9.19650853524589	7.82322557417022	9.05320552690255
C	13.79403425504024	6.72899899433505	1.40390271943089
H	14.34383838536177	5.84187026187149	1.10950157231777
C	17.41610651486728	13.93105766220615	3.52117724383210
H	18.10956017398713	13.21159411668045	3.09992120739604
C	16.23275350678048	14.15147712908677	8.57173843665268
H	17.25186110144645	14.18897218572715	8.92419405810682
C	15.63863803806756	15.76657806649004	4.63652416030272
C	13.87225848150440	7.08785587873913	9.39850497102632
C	13.35029285040634	6.84310909181692	2.71481097297719
C	15.08991145113736	14.52256956580396	9.26121218300406
H	15.02224596540823	14.91351395203751	10.26558137179381
C	8.39912988494344	13.18498561267741	8.16235770822931
H	7.85235564020655	12.36911200637261	8.62210829978547
C	14.30100366078183	8.34473631281251	8.96127507962108
C	8.41470725685283	15.27308761201906	6.98376339053779
H	7.88012249532504	16.09623135145685	6.52273684912188
C	15.71046975283914	8.76951930587933	3.79912015026250
H	15.09896076918167	8.99530764413657	2.93869462651553
C	17.88530180656634	15.12628790496680	4.03788751485511
H	18.94601358911036	15.34730679532606	4.01547843976662
C	16.99921157756640	16.03737347088977	4.58854866789516
H	17.36525288023157	16.97131788750172	5.00047107944395
C	7.71447867822894	14.23889452002877	7.58259844860495
H	6.63090049542493	14.25273825733220	7.59344994355657
C	16.05987548047825	13.62322952479093	3.54255782922600
C	9.59665491611302	7.51883068760299	6.89067848446868
H	8.69262006986970	7.07641478132199	6.49944729342368
C	10.60739473183485	13.87221716207578	2.39808485386001
H	10.60585719354370	14.20731721886386	1.37150413982156
C	13.88845837168066	9.27573399546570	11.12407055626421
H	13.89573798544771	10.12725558612515	11.79588204630411
C	9.55241891617300	13.33001498618524	3.11396911797635
H	8.54782833973538	13.14886165665025	2.76436351349166
C	10.04140363480089	13.05474547494100	4.40399604027064
H	9.50444113470069	12.63263193758866	5.24208686280183
C	9.80228230713444	15.26633733823111	6.95006097520964
C	17.41467823038700	8.28953607143314	5.16935921162664
H	18.38819626298599	8.06612515736645	5.57971661607053
C	14.32750056408698	9.45053618110826	9.81565769233068
C	14.68115458047627	16.72326373129464	5.27366393831716
H	13.83861751466404	16.95298828176136	4.61778990449405
H	14.26846401409725	16.29862821141790	6.19458478365358
H	15.18199588764844	17.65653788806286	5.53177148658746
C	15.55932288506842	12.31481425850316	3.01620508257372
H	16.39124090973654	11.66072078194141	2.75697886026439

```
H    14.94190240118593   11.80052394185714    3.75906552722000
H    14.93818635368531   12.44298647716343    2.12509362406387
C    10.56570180547330   16.35333514617840    6.26200689921874
H    11.32686574275579   16.79047794384289    6.91211011153856
H    11.08036027739906   15.96081735333428    5.37927970964992
H     9.89567666699515   17.14780816028748    5.93299365745858
C    10.52504459184479   11.98442778438657    8.75475915255020
H     9.82900720677000   11.20449676917766    9.06225029266536
H    11.22751930842160   11.54855106135593    8.03774593795948
H    11.11125812585906   12.27978612956382    9.62944665052038
C    11.60909256509490   10.22881600788085    2.56897413576847
H    11.59283003780713   10.96915593172605    1.76970791113730
H    10.57340319127195    9.97653469844051    2.81145031431069
H    12.03080827751578   10.68967597151768    3.46642509246729
C    13.64606898541166    5.77876951872878    3.72398867597892
H    12.73515438987540    5.37993317293274    4.17655611166766
H    14.18932816486895    4.95288942101755    3.26458918797808
H    14.25984087219402    6.18086289712373    4.53527482776052
C    13.82990871279914    5.92684524643630    8.45582238418834
H    14.81516517086428    5.70117250980677    8.04064808090295
H    13.46100486870892    5.03450459226275    8.96172227997442
H    13.16604635840732    6.13974461469483    7.61293740205550
C    14.81622286881955   10.77767190021465    9.33553813726601
H    15.87971683371556   10.74557487101893    9.08511747676091
H    14.29650767501237   11.08363003371518    8.42337125335424
H    14.67534156276257   11.54650448502421   10.09447498675251
```

[(pyrNMe)$_2$V=P=V(pyrNMe)$_2$] (S = 3/2)

```
Eh = -4678.020505156271
V    13.39829483358817    8.88012836162757    5.99190063809573
V    12.94842703983691   13.28322431154612    5.87053139148421
P    13.18194478720651   11.07950173860690    5.94344334976681
N    12.32489235650709    8.23262709914954    4.27320431704460
N    14.57058719974995   13.80789683127458    7.08070709503844
N    11.65534955486726    8.24224845798739    6.82955997284278
N    14.56817034552334    8.54162226999674    7.73635115063959
N    11.98396766889802   14.01967036103587    7.52273459029974
N    13.74961144605522   14.09761763054198    4.16684625261703
N    15.23346519723109    8.53962447377353    5.17706343825180
N    11.26251125649912   13.36923522158711    4.63888501859073
C    11.13962328011251    7.78849873670861    4.56599292159306
H    10.48240195559761    7.40625577834998    3.78520335327783
C    10.72640925319481    7.80849542673126    5.90729540493254
C    15.81618014172896    8.31058413769035    7.45867342667568
H    16.53189151120804    8.10377218525822    8.25412770625590
C    12.78410821147324   14.44186586267130    8.47194258986744
H    12.36956482692687   14.84999533492445    9.39298482616067
C    14.16142082769719   14.35151450219862    8.27795928902564
C    16.22254810174531    8.33237255226304    6.11543468751224
C    13.18674117722334    9.10797454361923    0.79352892747102
H    13.09565894353315    9.94856978309333    0.11426179107860
C    12.78187858889760    8.12847415076365    2.93616654381853
C    12.88358092406065   14.28066223622399    3.19939488392588
H    13.20740924161700   14.70911382340772    2.25156250841355
C    11.03595941744018    8.22810638940705    8.02465869061582
H    11.54608661971218    8.53983892732338    8.92277048449350
C    13.16410358149365    8.24487641863659   11.66231546587210
H    12.77300868464957    8.17032894465677   12.67025864193842
C    11.55544964207482   13.91300609955925    3.40815964022681
C    12.64902729345226    9.21844266544030    2.07149430706039
C    13.25462756972630    7.11433598494493   10.86791670800847
H    12.93140029234123    6.15267645016662   11.25094510705251
C    10.58023489598135   14.06202026101655    7.70973854724781
C    13.82241458007269    7.94953931329398    0.38097426408742
H    14.23762827202532    7.88402832899975   -0.61790052259475
C    15.90210761056529   13.77875564936191    7.11381731299470
```

H	16.47296190129597	13.39486919316636	6.28006242177314
C	15.11246518288444	14.42875452662138	3.96496609587582
C	9.94432517232697	12.99484135802673	8.35083763233347
C	17.22220958038312	8.38481546332782	4.12656022612977
H	17.94778479566821	8.36762269571626	3.32797551850943
C	9.72274518975073	7.78178955236003	7.89472480184822
H	9.00798174473840	7.67365724133247	8.69593395464141
C	13.92088637767069	6.87242686500526	1.24535097776053
H	14.41639963726284	5.96224645255431	0.92591463127269
C	17.31539838250499	13.80887399440253	3.27453593824613
H	17.99310710771521	13.08229433865316	2.83994923640245
C	16.38652670428134	14.30486101177114	8.32452124463245
H	17.42424761028002	14.39898398835479	8.60478927402272
C	15.57755726286403	15.66909942531181	4.40942841953021
C	13.74038011726356	7.19368647857008	9.56909051479548
C	13.40618124583767	6.94338654158110	2.53349139750904
C	15.27642206245442	14.67256369064381	9.06910271415231
H	15.25842749821399	15.11493722279074	10.05406177527095
C	8.55713406726493	13.02540002195486	8.44854207104937
H	8.04824900109564	12.19993546342047	8.93403661644111
C	14.14095878841187	8.44271419014369	9.08365398452191
C	8.47975889730527	15.13306470283562	7.30826778250668
H	7.91018741494056	15.96036487191404	6.89943421951201
C	15.85013245484088	8.57590540530594	3.98088638744925
H	15.29561673886347	8.74118570912300	3.07026491718392
C	17.79551942610787	15.03218410769250	3.70863783733570
H	18.84930256417455	15.26630643713878	3.61250717352745
C	16.92836535719885	15.95618445739291	4.26748530241267
H	17.30259905767800	16.91379729293853	4.61232595710419
C	7.82780768023835	14.08216299031087	7.93182695022557
H	6.74721282740164	14.08684579271221	8.01437311329949
C	15.96788531517230	13.48392842577572	3.39079261466471
C	9.52238166276877	7.50952563125709	6.54008447190345
H	8.62218609054795	7.14748495609781	6.06619373728018
C	10.40348903065209	13.94095097955596	2.60562030962287
H	10.33463665603802	14.31560632561725	1.59516794852981
C	13.58031802548742	9.46915867605004	11.16847320945183
H	13.52332379735274	10.35397511769994	11.79314752583271
C	9.39140313645285	13.39214418843238	3.37793940057953
H	8.35941127884858	13.24646332832418	3.09857078357511
C	9.96760477949679	13.05619729934580	4.61577857704881
H	9.48565459566237	12.61086935898946	5.47472381958700
C	9.86231437803268	15.13865153748148	7.18275936219817
C	17.46357633708749	8.22579263945225	5.49258200367036
H	18.41285346938369	8.06023277582190	5.97986632310671
C	14.08311102097338	9.59189493152647	9.87746832934872
C	14.64437334625686	16.64317422764261	5.05631959221365
H	13.78203611220958	16.86498780781731	4.42339379258628
H	14.25894665045615	16.23780971745104	5.99698675719410
H	15.15717204154083	17.57920486269682	5.27817738139165
C	15.45574285043406	12.15478357032874	2.93430463030253
H	16.27555379009346	11.52255313309285	2.59377096700771
H	14.93282424976245	11.63548521689325	3.74284265543228
H	14.74168618584875	12.25549167345988	2.11226425986521
C	10.57006341892139	16.24815635165531	6.47111676690578
H	11.34632717116604	16.70058423535936	7.09265919068959
H	11.05821599502812	15.87491018681741	5.56582057388146
H	9.86713481572229	17.02814541746667	6.17824061809876
C	10.72843479131164	11.84140402278120	8.89170284693698
H	10.06334967792496	11.08273747365175	9.30362008852365
H	11.33531801628574	11.37779428405245	8.10789220325423
H	11.41981739916697	12.15201551218582	9.67958461836515
C	11.93982553417172	10.45982137942414	2.50264178232517
H	11.95851731694464	11.21370271871319	1.71622089162879
H	10.89427446063395	10.26193705076680	2.75055549544220
H	12.39171952106228	10.88564477751991	3.40247459106249
C	13.55260924791474	5.79646495709940	3.48381665827369
H	12.58501719439171	5.42681427336802	3.83148107631039

H	14.08167356256816	4.96995223249539	3.00932042623846
H	14.11854016788653	6.09878382907081	4.36956025333159
C	13.79297831339355	5.98152606437820	8.69220262594337
H	14.80673932647611	5.77297182935318	8.34219601670969
H	13.43072425975136	5.10425475383650	9.22819051774548
H	13.16818148412712	6.12108660319601	7.80545265920138
C	14.56089677289017	10.90875828534680	9.35943842596077
H	15.62571266484447	10.88288509694260	9.11559898909049
H	14.04336915469044	11.18605410193645	8.43704862501588
H	14.40537989473169	11.69879595885873	10.09341106871122

[(tBuPhN)$_3$Cr=P=Cr(NtBuPh)$_3$] (S = 1/2)

Eh = -5093.169294705745

Cr	2.86522822594953	10.30955749067753	2.10663598000013
Cr	-2.60353142498826	10.70248148004314	-1.98368634846211
P	-0.83139038638533	10.59982699939535	-0.81612932519201
N	2.60856925876899	8.51534485576638	2.48259100190929
N	2.24027386513106	11.58560716217947	3.29054548623919
N	4.07960153194194	10.80079111036947	0.80047049158659
C	3.17429983014451	8.18593341093516	3.73513231039922
C	4.55764065875537	8.05959561980980	3.87558114339107
H	5.18295807781047	8.15888155703365	2.99666659745485
C	5.12759508036506	7.84527458565339	5.12055944709662
H	6.20570956691152	7.77223666949872	5.20656993869939
C	4.32806369156229	7.73512994064101	6.24786393208290
H	4.77401365908706	7.57113176283131	7.22183530228352
C	2.94958445814049	7.84648058158567	6.12060767062658
H	2.31607582065380	7.77548994927383	6.99763467759488
C	2.37934585834507	8.07317882656913	4.87936629702296
H	1.30820744132028	8.20268693435429	4.78456263710596
C	2.04069329540149	7.44223482903897	1.65772811849961
C	1.68947849981693	8.04237980762151	0.30527941151312
H	2.58058923835907	8.44228447177887	-0.18351138794658
H	0.96565482448381	8.85591577538707	0.41352830300384
H	1.24429265172973	7.28716961018572	-0.34646021709544
C	0.77699893914084	6.86003871832321	2.29139835859604
H	0.99598472418184	6.40471231292575	3.25876975158499
H	0.35292238209347	6.08674251222557	1.64547920831034
H	0.02402162699575	7.63865976991389	2.43408949411016
C	3.31816511689917	12.18161455177436	3.98384776940371
C	3.82805144489753	13.42385737731389	3.59576539548760
H	3.35414176489770	13.94913266617364	2.77580358894726
C	4.94837806399140	13.95546105884844	4.21166210210445
H	5.33656016705398	14.91391547723889	3.88572658549409
C	5.58884233215680	13.25455998652147	5.22520461804658
H	6.47378317121824	13.66526433136845	5.69700598597219
C	5.09190922052302	12.02229548371524	5.62097575788500
H	5.58780379441259	11.45894930056408	6.40311341758618
C	3.96364952744973	11.49407647305438	5.01359869365600
H	3.58252920666455	10.52526376138164	5.31260540849501
C	0.86722468758065	11.95417422674589	3.65552923511675
C	-0.06937341751276	11.04433980014260	2.87580401544033
H	0.12305863667671	9.99559805186877	3.11372713308018
H	0.06582646577491	11.18058107198438	1.79829713978319
H	-1.11187797720836	11.27000763501192	3.11039961019913
C	0.64708623743707	11.74661187306350	5.15248125473354
H	1.30285712549288	12.39389290806758	5.73825709360136
H	0.85798331382071	10.71169526741719	5.42956750687632
H	-0.38684126192835	11.97634209927338	5.42166079001043
C	5.35851924477863	10.28013745177733	1.10196202808760
C	6.13754307766084	10.85681559662287	2.10723135041508
H	5.77141622194936	11.74658572705014	2.60484466760938
C	7.34144674183661	10.28558785235424	2.48776847416114
H	7.91730724462784	10.74004705956775	3.28581514296258
C	7.80106207040212	9.13703396779687	1.86165733915124
H	8.74007286304209	8.68736984401991	2.16238608285245

C	7.04270433832925	8.56047119340085	0.85105715012163
H	7.38632022073292	7.65491301628968	0.36375237423372
C	5.83394640805189	9.12337301650692	0.47763679505890
H	5.22158467503017	8.65155583203397	-0.28084178465372
C	3.92757553394363	11.69538731550385	-0.35380914510665
C	2.52426640855285	12.27930080249031	-0.29556636475279
H	2.38934681913768	12.86837892099980	0.61400602819527
H	1.77123126430230	11.48512705230242	-0.30259086554608
H	2.33667713921823	12.92132269561430	-1.15928916869323
C	4.10263027468645	10.93871864727387	-1.67018658203217
H	5.10505639005232	10.51432914910288	-1.74927518404591
H	3.95648085813507	11.61318047337810	-2.51804866884050
H	3.37333648620873	10.12893896489740	-1.74676954544988
C	3.06793750351338	6.32828618963542	1.46158863442400
H	3.33662125445585	5.86863569601737	2.41480483860071
H	3.97835744834774	6.72476183795704	1.00738939584756
H	2.66351363481298	5.54935778338647	0.81050916478609
C	0.56851440969050	13.40878238452313	3.29317256597413
H	1.21089196857839	14.09317535216159	3.85006420059957
H	-0.46982918980421	13.65428483412085	3.53112892305222
H	0.72445750821407	13.57461746138667	2.22475978110743
C	4.94753639995961	12.82976821360719	-0.27779632118766
H	5.96803087787475	12.44688570742582	-0.34135594111817
H	4.84561625485539	13.36730321613506	0.66709401860851
H	4.79587764199672	13.53412432604180	-1.09934394519909
N	-3.03260528482401	12.49906720302801	-1.82488726742237
C	-3.70207372890726	12.92933397486756	-3.00120223919031
C	-5.07746526277670	12.75384144043918	-3.13206925289350
H	-5.62719695263946	12.29548097929711	-2.32180570620622
C	-5.73822528323844	13.15855442228468	-4.28221675768067
H	-6.80729994505278	12.99965652774541	-4.36382662656373
C	-5.03999293185361	13.75722658374683	-5.31776404484007
H	-5.55562848866943	14.07240382071453	-6.21723560999873
C	-3.66893592913080	13.94910984479545	-5.19418202023751
H	-3.11038409322563	14.41308848100934	-5.99916825316779
C	-3.00826291300131	13.53689305265071	-4.05117703889390
H	-1.93816458942169	13.66638385322410	-3.96529614995109
C	-2.68186098852844	13.56837270055611	-0.84938417315631
C	-2.75906859644539	13.01490109261814	0.56776775492574
H	-3.77013388946365	12.67079022208802	0.78603755716276
H	-2.07438383318858	12.17935356308094	0.71286687167855
H	-2.49462472246544	13.79541882007758	1.28474161561972
C	-1.27542482624646	14.10282651895785	-1.11310140668246
H	-1.21224001887150	14.57730021280909	-2.09360782543527
H	-1.00488923672269	14.84790094107658	-0.36051960811447
H	-0.54319341189318	13.29331877680303	-1.07133015068147
C	-3.68001688463106	14.72300467263419	-0.94043123748329
H	-3.64361057694641	15.22104288265564	-1.90978453919089
H	-4.70046083124373	14.37128936543213	-0.77523170754895
H	-3.44164739787310	15.45917083558951	-0.17023496195829
N	-1.96166413627825	10.14911685698794	-3.63110703287637
C	-2.99513729403574	9.48373496300521	-4.34300061260805
C	-3.13115812983146	8.09389061701986	-4.29642215947514
H	-2.44236211300692	7.52097253390182	-3.69072937747195
C	-4.14306976964414	7.45698435963775	-4.99203163068401
H	-4.23483236783754	6.37813778294904	-4.93829114399659
C	-5.04790813154058	8.19617468476946	-5.74486485767606
H	-5.84588446177917	7.69699139602919	-6.28182374293009
C	-4.92152709365982	9.57450647236071	-5.79854984542756
H	-5.62124601198406	10.16558255903778	-6.37806767058198
C	-3.90103495800808	10.21235081668646	-5.10970109678391
H	-3.79941176593968	11.28756128049467	-5.15934904293304
C	-0.61179686682551	10.10871621772955	-4.25835759140704
C	0.14328221390234	11.39419157918948	-3.94199073739978
H	-0.40014117983208	12.25632755681527	-4.32917052949538
H	0.28299095916624	11.53193091944645	-2.86913377308316
H	1.13048577885901	11.36580832646166	-4.40864796787973
C	-0.73391683845711	10.02025502429756	-5.78001032931897

H	-1.20791067118389	9.09201546749284	-6.10042714828873
H	-1.31802963140712	10.85476125445762	-6.17316435941529
H	0.26417300365495	10.06193428285916	-6.22062225274532
C	0.18450657410740	8.90859856107125	-3.74978464396053
H	-0.29108521651324	7.96941511807156	-4.03680202911937
H	1.19358519015634	8.91694689789395	-4.16986315657534
H	0.26730112389883	8.93828673961302	-2.66090922772699
N	-3.68795584184462	9.50777219382446	-1.07056570381259
C	-5.04573636920370	9.90050220904845	-1.21178580561705
C	-5.78455428907174	9.49131934993365	-2.31918289695330
H	-5.31185303669502	8.86346610284251	-3.06177955472058
C	-7.10619747062781	9.88163169150012	-2.47398324889732
H	-7.65569613644084	9.55960096177837	-3.35100211934687
C	-7.71823872676671	10.67723669808491	-1.51953336992131
H	-8.75035250048700	10.98422584267083	-1.64100147269548
C	-6.99521001971790	11.08178808277105	-0.40360995895667
H	-7.46181974808274	11.70806534468958	0.34825845447671
C	-5.67410691056664	10.70047905280625	-0.25384231626118
H	-5.10554256275317	11.03515515327625	0.60276136926419
C	-3.43260045013636	8.38708806396773	-0.12311361909319
C	-2.26367316410880	7.54541702250217	-0.62127779873924
H	-2.49263383147603	7.11936057046034	-1.59828799145194
H	-1.34814174923046	8.13079060253367	-0.70933005867076
H	-2.07491922466802	6.72877904157189	0.07906647235360
C	-3.12075634217966	8.91988869454473	1.27384216515081
H	-3.97007361069456	9.46794537601742	1.68474165698219
H	-2.89181841020368	8.09501318161206	1.95354358628197
H	-2.25784744444387	9.58863703613863	1.24635337042346
C	-4.64996692264158	7.46487363179631	-0.04551004216575
H	-5.52432024270681	7.97083205953076	0.36519321432259
H	-4.91204478781018	7.07957255163267	-1.03299562196285
H	-4.41304680535101	6.61757570106652	0.60100920622372

[(tBuPhN)$_3$Cr=P=Cr(NtBuPh)$_3$] (S = 3/2)

Eh = -5093.191238868577

Cr	1.96224469764033	10.36645851432341	1.44141453806284
Cr	-1.96249218118205	10.64610551941308	-1.44132942948933
P	-0.00022312719502	10.50615777626696	-0.00016657703185
N	2.20597077473792	8.51708785236777	1.72891198409705
N	1.48520962486314	11.32456238107036	2.99463427172844
N	3.33662041063364	11.18045049805361	0.43882051812378
C	2.76781699041965	8.25561568919021	3.00810412011847
C	4.14878637035058	8.25397285656193	3.20318504923207
H	4.79834718867777	8.46608918900098	2.36654935754664
C	4.68963029884999	7.99240956868355	4.45141767660569
H	5.76612791600685	8.00968353516801	4.57776554740564
C	3.86358604495358	7.72161965330853	5.53137050738625
H	4.28588009328662	7.52253805499423	6.50925655193166
C	2.48751408383894	7.71067049451399	5.34855705532245
H	1.82959204426612	7.50302990663854	6.18492985214661
C	1.94771530311111	7.97312535358137	4.10045483167600
H	0.87457319464840	7.97466928808132	3.95816923385405
C	2.07405160714704	7.29515226988073	0.89692594690768
C	1.66269588846357	7.67243349878645	-0.51189707875509
H	2.35322186383123	8.40010237007341	-0.94273222932466
H	0.66093778853991	8.10238250952706	-0.53117441976792
H	1.66134534133088	6.78218177546224	-1.14383686992221
C	1.01915559523843	6.36264365777106	1.49395621836940
H	1.33373786029420	5.98259132955457	2.46713216685353
H	0.86506502326002	5.50554205691650	0.83350883428470
H	0.06983387720769	6.88259255118056	1.61312887465416
C	2.60986518519096	11.95716690425520	3.59136883243302
C	2.91873204800468	13.28422690902251	3.29312165000518
H	2.29401155687635	13.81569209940383	2.58678247123751
C	4.00851892945794	13.91099464174080	3.87394847294902
H	4.23407260755333	14.94054084000676	3.61970963291383

C	4.81430677590033	13.22275216293338	4.77052292283717
H	5.67077326589326	13.71093051344506	5.22043745510721
C	4.51263906905677	11.90580312158360	5.08153664627036
H	5.13392484666330	11.35277207018751	5.77675994857671
C	3.42137419849292	11.28005218665794	4.50126222985681
H	3.19453059070065	10.25300540359469	4.74700737062416
C	0.24532953460169	11.46809648009822	3.79738643647555
C	-0.85289084016851	10.60222804848341	3.21206976177183
H	-0.52722251870293	9.56499423182066	3.11056570477737
H	-1.15887395352497	10.95962644016652	2.22843002551760
H	-1.72575339807848	10.62670065973804	3.86742179613927
C	0.45707803939592	11.03738472462009	5.25184896501784
H	1.18569888743243	11.66908908068617	5.75982311585465
H	0.79484870455218	10.00351023062987	5.31450483337071
H	-0.49190369265082	11.12359242085040	5.78595382515552
C	4.60175271400970	10.58852878894300	0.70005077309985
C	5.40106843872271	11.02350145781250	1.75721377660296
H	5.04444138472814	11.81635918348908	2.39905119776923
C	6.63597092736645	10.44339881441882	1.99796651327401
H	7.23206803428504	10.79255651982435	2.83337959228830
C	7.10138485553034	9.41940066463310	1.18743375526950
H	8.06488151007876	8.96223712327780	1.37953202715087
C	6.31883832944342	8.98278952398106	0.12730211846932
H	6.66895804977013	8.18071958441009	-0.51268060377322
C	5.08457870034360	9.56293155087796	-0.11288512034551
H	4.46837874668989	9.21832855013634	-0.93356726835098
C	3.42178991934610	12.36389025593977	-0.45293577792944
C	2.07113884233313	13.04715249296635	-0.53080820755923
H	1.68531590705270	13.27114036058598	0.46564783192981
H	1.33903368796646	12.42314270752684	-1.04436734104559
H	2.16483469376566	13.98386816337957	-1.08355900654236
C	3.84926202573364	11.93701729147160	-1.85754531245436
H	4.86733017825234	11.54472298456241	-1.85723334633269
H	3.82148537371141	12.79620967380313	-2.53255354406368
H	3.18139229285482	11.16971717966642	-2.24664850215450
C	3.39275457450464	6.52073741942381	0.80795807925264
H	3.70904553841033	6.14609746489451	1.78139711296687
H	4.19117318854197	7.14094192879936	0.40149436920301
H	3.25230401105949	5.66165576778202	0.14798114872724
C	-0.21683546145850	12.92574613016434	3.79866406369806
H	0.49988148829369	13.56655319463695	4.31439770881438
H	-1.17539785889795	13.01115661464114	4.31676444294352
H	-0.34090199297653	13.28985849699928	2.77976480720803
C	4.43829960393198	13.38826763724362	0.05988195707875
H	5.44954472128004	12.98192845089202	0.07776211277673
H	4.18742450123314	13.72834739057519	1.06392689627904
H	4.43705418434316	14.25270223521989	-0.60819923941663
N	-2.20645886334207	12.49544939068088	-1.72854260823188
C	-2.76811090359868	12.75703157721071	-3.00779235147539
C	-4.14903272067462	12.75863590179925	-3.20310748081888
H	-4.79875085559453	12.54649077052442	-2.36660069262740
C	-4.68965676247895	13.02022226236966	-4.45143589000764
H	-5.76614140521450	13.00296508000715	-4.57795878059214
C	-3.86341663473178	13.29099138865514	-5.53124661654219
H	-4.28553344784905	13.49007208397516	-6.50921161602528
C	-2.48737707428558	13.30190720316904	-5.34818631036044
H	-1.82929551408102	13.50952496955107	-6.18443949284474
C	-1.94781522464146	13.03943876299144	-4.09998689852006
H	-0.87470528308553	13.03789603084808	-3.95750238492367
C	-2.07460742345216	13.71729866071871	-0.89644824122740
C	-1.66265663833851	13.34002716666535	0.51220595096010
H	-2.35282240311583	12.61212169900669	0.94322647115655
H	-0.66075160592360	12.91038509154713	0.53110975299566
H	-1.66136145761146	14.23022650052076	1.14422997537932
C	-1.02021320464133	14.65015808816582	-1.49378571973073
H	-1.33526931172516	15.03017840611564	-2.46681478814480
H	-0.86613933006261	15.50726359520853	-0.83333412156372
H	-0.07078735912050	14.13049350548876	-1.61333590970558

C	-3.39352763381355	14.49128630835883	-0.80697703040124
H	-3.71019615734989	14.86600150814878	-1.78026556085739
H	-4.19165306695091	13.87076440673336	-0.40041888150963
H	-3.25317336018946	15.35029555649563	-0.14688605078470
N	-1.48497342179531	9.68847643554465	-2.99466609942420
C	-2.60929906324392	9.05549834542975	-3.59156373420608
C	-2.91781806828169	7.72842966104826	-3.29315743708999
H	-2.29312519579503	7.19731948533967	-2.58655031481864
C	-4.00722683601188	7.10121110074023	-3.87418532702660
H	-4.23253971333340	6.07164365877838	-3.61983325023350
C	-4.81300074695861	7.78904697112726	-4.77109059846708
H	-5.66919589442708	7.30052116822164	-5.22114277218733
C	-4.51168222287094	9.10604692745020	-5.08224556136580
H	-5.13295229430147	9.65878125218590	-5.77773600066657
C	-3.42076230852101	9.73224000386401	-4.50176981614382
H	-3.19420538715615	10.75932849975292	-4.74761245475788
C	-0.24502393463363	9.54527843102046	-3.79726478285117
C	0.85307074027349	10.41106065724149	-3.21159160237731
H	0.52722690809522	11.44818491466513	-3.10960777878881
H	1.15914125011583	10.05327983058526	-2.22813307917104
H	1.72591242495677	10.38703998520580	-3.86698280312247
C	-0.45664856587715	9.97641689452411	-5.25160726158561
H	-1.18516687375762	9.34483031890144	-5.75986668667818
H	-0.79447896581204	11.01028300267544	-5.31399052806739
H	0.49239761392878	9.89044397711227	-5.78563184533516
C	0.21731755857795	8.08768541477568	-3.79894669715789
H	-0.49921052823806	7.44695381066050	-4.31501327980236
H	1.17597818955584	8.00257369143637	-4.31690562442002
H	0.34125962715237	7.72326172313855	-2.78015209663801
N	-3.33685872430130	9.83186395074082	-0.43893778745020
C	-4.60195771701845	10.42381197009471	-0.70030394245226
C	-5.40096708008186	9.98913067152767	-1.75782086049057
H	-5.04412447143729	9.19650241700123	-2.39982364735131
C	-6.63580436735978	10.56932729932764	-1.99874862691435
H	-7.23166756464738	10.22042792899434	-2.83444135313704
C	-7.10143216129045	11.59311573180166	-1.18806097660517
H	-8.06485119669476	12.05037630745926	-1.38033077582276
C	-6.31920434503464	12.02940082396248	-0.12755951087289
H	-6.66948973447247	12.83129023924096	0.51256605872498
C	-5.08503177471441	11.44917588748173	0.11280348661579
H	-4.46905384477316	11.79354848557164	0.93375687750668
C	-3.42215426816940	8.64843049784444	0.45283871430152
C	-2.07157847641316	7.96501447749867	0.53071716875533
H	-1.68579182864382	7.74091762904935	-0.46572488413564
H	-1.33939788879055	8.58898534499215	1.04422585434633
H	-2.16537820243799	7.02835471511785	1.08354220414531
C	-3.84954432075745	9.07535063873460	1.85746792713931
H	-4.86756445113795	9.46778270291309	1.85720614164286
H	-3.82186146449302	8.21613226667241	2.53244955936191
H	-3.18153533842636	9.84254723910406	2.24657564529039
C	-4.43882867723154	7.62420951506961	-0.05997978714058
H	-5.45001271180547	8.03071147277278	-0.07783833733942
H	-4.18800632085026	7.28412045555939	-1.06403150657316
H	-4.43769923890306	6.75977333180843	0.60809942349970

[(tBuPhN)$_3$Mo=P=Mo(NtBuPh)$_3$] (S = 1/2)

Eh = -3141.169316352663

Mo	1.76466605146496	10.34795971289095	1.32268496411077
Mo	-1.76439235412672	10.66493487821714	-1.32292191358792
P	0.00019525672904	10.50634964158233	-0.00020552004042
N	2.12217187863596	8.41576546881475	1.53096691732215
N	1.30691103521640	11.32129209756110	2.98747531843946
N	3.21526377548269	11.27711009283795	0.34521379770703
C	2.66968266485123	8.20905977179966	2.83249493297541
C	4.04639144634227	8.23840891546011	3.04963317458091
H	4.70483953008701	8.44281187966552	2.21801268921632

C	4.57067834362849	8.01030661267299	4.31224228671910
H	5.64421675333759	8.04946471573307	4.45636104135790
C	3.73081430798966	7.74337422412098	5.38159278299266
H	4.13998011031491	7.56691897696362	6.36941966918516
C	2.35669096918639	7.71185074359803	5.17918101420922
H	1.68942873554853	7.51284883304103	6.01007589228754
C	1.83377093904278	7.94237977303279	3.91891994365560
H	0.76310917323836	7.92823918456560	3.75702017614319
C	2.00016775766218	7.15973875945647	0.74573936806756
C	1.51937102087512	7.48849921504837	-0.65042892369353
H	2.20494439129717	8.17845431040939	-1.14684446979963
H	0.53320255566741	7.95004842716006	-0.63437637265598
H	1.46973295262325	6.57160304359121	-1.24141931164627
C	1.01567682766405	6.19671774364424	1.40668635010717
H	1.38221185781266	5.85661802152701	2.37658155415379
H	0.88057907942436	5.31581353822880	0.77420852532450
H	0.04654206975688	6.67180504801760	1.54626671575001
C	2.46875293995889	11.93247791988341	3.54376528240075
C	2.79284124778833	13.25166522317941	3.22313545587309
H	2.16821169153837	13.77784994238432	2.51231473981290
C	3.89362999390764	13.87081467546496	3.78842309424063
H	4.13131726782439	14.89432884661531	3.52175277177934
C	4.69762354690025	13.18036363780247	4.68716552233723
H	5.56225905678442	13.66372785235738	5.12667625530721
C	4.38953149327766	11.86805978937112	5.00796924274986
H	5.01439794423651	11.31337519733167	5.69851334189268
C	3.28450259838714	11.25006377823603	4.44323563929981
H	3.04694368752703	10.22705712965079	4.69650184248676
C	0.09563072193704	11.52778166302559	3.82740946828283
C	-1.06403632648566	10.74921783731563	3.24660155957791
H	-0.83036546825208	9.68385135474765	3.19454186917347
H	-1.30809845554687	11.08725861328804	2.24135070868862
H	-1.94021637176458	10.87615324742884	3.88577315735965
C	0.32745431244829	11.02190684296339	5.25352308647999
H	1.10295412260210	11.59051415195476	5.76696997396367
H	0.61145349934565	9.96965168438869	5.25581659997460
H	-0.59947674052113	11.13054979738929	5.82135207045083
C	4.47260647102219	10.68061392505667	0.66786226285422
C	5.25014328693694	11.13617091544462	1.73003667678794
H	4.88847397148036	11.95357151090325	2.33571791584873
C	6.47733028108949	10.55596174006915	2.01263949352148
H	7.05630215467632	10.92292416038798	2.85233992937520
C	6.95812938308449	9.51624938141414	1.23334787090008
H	7.91706659549785	9.06259114881572	1.45475900374177
C	6.19604669714660	9.05579031393001	0.16689141061082
H	6.55706241083725	8.23776633663914	-0.44618942766209
C	4.96830194457395	9.63086312805738	-0.10848839481071
H	4.36332092094669	9.26508689557649	-0.92837901369905
C	3.35536552139142	12.38378391067383	-0.64135998975975
C	2.01790122629594	13.06241599570854	-0.84164691911452
H	1.62814297314714	13.43007923613723	0.10959154101162
H	1.27549568562943	12.38863594025206	-1.26647049508139
H	2.14643827453140	13.91341775138989	-1.51422802235199
C	3.88120393470307	11.87963967330288	-1.98412601824805
H	4.90958316269862	11.52487916633265	-1.90190113969540
H	3.86692664893147	12.69143404856003	-2.71560122432215
H	3.26193829362913	11.06861071023747	-2.36084085928319
C	3.35276279383539	6.45312091409871	0.62589735631476
H	3.72939079735839	6.12652590947483	1.59549773401121
H	4.09836716181705	7.10311005233742	0.16794494961953
H	3.23501558269656	5.56761675536822	-0.00289796694157
C	-0.27403032279863	13.00832684208203	3.90150667276047
H	0.49396748741172	13.58544539532936	4.41855069761903
H	-1.20862753297773	13.12775488740982	4.45512425054291
H	-0.40927396969293	13.42181122928677	2.90437903466712
C	4.33062457317224	13.44108715704121	-0.11376240756339
H	5.34108792601328	13.04687354499739	-0.00501159849571
H	4.00491513430360	13.82525355479400	0.85334698567358

H	4.37077012446142	14.27346347773724	-0.82025385467673
N	-2.12195903353323	12.59715284358538	-1.53081389787439
C	-2.67053775329062	12.80390385380756	-2.83187310991041
C	-4.04735680175680	12.77310055353054	-3.04812343177361
H	-4.70502182964520	12.56730630054677	-2.21621733876676
C	-4.57271078214084	13.00133496015266	-4.31027137827653
H	-5.64627941440310	12.96099303905070	-4.45377589983030
C	-3.73385892596445	13.26996666050238	-5.37998873772934
H	-4.14387260417837	13.44662380287035	-6.36742836288779
C	-2.35965107971053	13.30296508537153	-5.17844545643111
H	-1.69316585830374	13.50336355243065	-6.00962716581382
C	-1.83563726565365	13.07219183938892	-3.91869030835213
H	-0.76489111538580	13.08763453306557	-3.75745491193695
C	-1.99955159054907	13.85309564335819	-0.74556461843308
C	-1.51861952826704	13.52409613031410	0.65048274232594
H	-2.20440716733227	12.83444929028016	1.14703124662901
H	-0.53265530899785	13.06211514051897	0.63423767147187
H	-1.46846601266859	14.44096007180744	1.24147935309317
C	-1.01488526495792	14.81580855111411	-1.40668922420571
H	-1.38137563577884	15.15581775039833	-2.37663692603210
H	-0.87959103011187	15.69678855434238	-0.77436143290225
H	-0.04584606308140	14.34050606144556	-1.54621506192442
C	-3.35190303424039	14.56012849326969	-0.62548540362975
H	-3.72860005718619	14.88687290148736	-1.59501232049090
H	-4.09761733795112	13.91037417651055	-0.16740028293797
H	-3.23374518957159	15.44559208524503	0.00329243282636
N	-1.30680883084590	9.69195800944022	-2.98794535945789
C	-2.46870440074368	9.08085241727758	-3.54431126229539
C	-2.79213480447777	7.76127066690498	-3.22464091349327
H	-2.16710945059899	7.23480506259596	-2.51437528475043
C	-3.89257365816353	7.14193294926368	-3.79039523364091
H	-4.12961680527965	6.11804317605679	-3.52459685866571
C	-4.69688509243125	7.83257953810335	-4.68867774340223
H	-5.56110110971737	7.34897115110946	-5.12875015191013
C	-4.38959868814012	9.14534826443754	-5.00836582043492
H	-5.01474256424567	9.70019585774466	-5.69852587527555
C	-3.28492744938068	9.76355677463757	-4.44315279076237
H	-3.04799711171472	10.78691089017351	-4.69563554709770
C	-0.09551152614923	9.48573855020401	-3.82797771054450
C	1.06379014630284	10.26517391102438	-3.24761524286395
H	0.82957613002035	11.33044007238492	-3.19596862991887
H	1.30812680643006	9.92767881250223	-2.24225487763347
H	1.93997994572535	10.13841836689879	-3.88680861734739
C	-0.32770723954473	9.99080023973662	-5.25432367781093
H	-1.10314367742410	9.42169611338726	-5.76730804891967
H	-0.61201154729697	11.04296373090776	-5.25714295981342
H	0.59914880195193	9.88209694960974	-5.82227008596755
C	0.27504763882189	8.00537308372006	-3.90137500592915
H	-0.49259231195932	7.42751314080788	-4.41811995770208
H	1.20971195117296	7.88625029648247	-4.45494783618692
H	0.41057681269342	7.59246993097136	-2.90403654525856
N	-3.21470707619262	9.73532006902304	-0.34544016517063
C	-4.47233916658000	10.33131699871985	-0.66789974091146
C	-5.24995815516075	9.87542508539820	-1.72988793998901
H	-4.88787276785492	9.05855094568691	-2.33605870255967
C	-6.47790922070842	10.45454997562587	-2.01152277376521
H	-7.05708663257164	10.08731495538736	-2.85095681179326
C	-6.95938983504094	11.49334444299768	-1.23143509697162
H	-7.91902981571372	11.94595888008266	-1.45195305101666
C	-6.19714161784478	11.95420092222559	-0.16528687063706
H	-6.55872147911546	12.77150606007468	0.44842544234376
C	-4.96867986127832	11.38023635010319	0.10916416049985
H	-4.36374715001514	11.74605284212758	0.92907878953865
C	-3.35452289603679	8.62839764219465	0.64086936851767
C	-2.01698283462225	7.94983805252612	0.84083825969221
H	-1.62721250880466	7.58255055298757	-0.11053680622764
H	-1.27465250854191	8.62357262238472	1.26584649625785
H	-2.14536836476016	7.09860275988101	1.51315118627254

C	-3.88020642279160	9.13217289055042	1.98383999163638
H	-4.90864953409801	9.48681365876753	1.90187926880573
H	-3.86570199096879	8.32020020210840	2.71511004731686
H	-3.26099027793385	9.94318576162452	2.36068260132787
C	-4.32989752535179	7.57122486091084	0.11321830328184
H	-5.34033601623602	7.96555055245396	0.00456867588491
H	-4.00429804169706	7.18710014289256	-0.85393401851232
H	-4.37009206592114	6.73881151921768	0.81966450788597

[(tBuPhN)$_3$Mo=P=Mo(NtBuPh)$_3$] (S = 3/2)

Eh = -3141.151156932854

Mo	1.81230942725561	10.29734547424225	1.32196784475720
Mo	-1.77343674806930	10.68452578757149	-1.43154914523286
P	0.22148992569875	10.35077633326672	-0.37056484971809
N	2.11055666101011	8.37441552528441	1.71704580856784
N	1.36375762656991	11.33653442779688	2.93994085419073
N	3.34236348897905	11.21398014466320	0.44600149146216
C	2.59716925077778	8.21315226880275	3.04814712280600
C	3.96299601474705	8.24501169591787	3.32659921945211
H	4.65971613705813	8.43214596752615	2.52228514661104
C	4.42854826940105	8.04442621104806	4.61592818951277
H	5.49463231922140	8.08316662754809	4.80773475567089
C	3.53948578091010	7.80874399851126	5.65308910271954
H	3.90263925062692	7.65581865989276	6.66254266431664
C	2.17637610316762	7.77787941430974	5.38925620670563
H	1.47105977937971	7.60179023383195	6.19348971848646
C	1.71234912933466	7.97610681810884	4.10001228513087
H	0.65062644313078	7.95435941608865	3.88791440723323
C	2.07380325518554	7.08335980186998	0.96487716448737
C	1.68553899794472	7.33071336540174	-0.47752352135348
H	2.37452090805053	8.03103176401529	-0.95440273679175
H	0.68557055517164	7.75053599126031	-0.55492587226960
H	1.71514717720332	6.38832034272968	-1.02826179787604
C	1.06793897701480	6.12741692291633	1.60429084160696
H	1.38423973966774	5.82970348182765	2.60515609234438
H	0.98219317989754	5.22245280257356	0.99790555862689
H	0.08562018089199	6.59059513999615	1.67343389924413
C	2.48943696111679	11.94321873507555	3.56656303051851
C	2.87572993181054	13.23925189281491	3.22246445339072
H	2.31558929100973	13.76254370516481	2.45888740506613
C	3.96144722319339	13.84412897890462	3.83145771876413
H	4.24746690151314	14.84938118623155	3.54341241865666
C	4.68651504791599	13.16381941760033	4.80140912891134
H	5.54042436025979	13.63422195473802	5.27455297455570
C	4.30914037565739	11.87764115096615	5.15377782459980
H	4.86803678309127	11.33132116751747	5.90498480486505
C	3.21961063462142	11.27408809269189	4.54569187278324
H	2.93132779051701	10.27130490818364	4.82465770440930
C	0.08886553340704	11.56775318370289	3.66913303649440
C	-1.01813030205560	10.78205602113793	2.99758928983988
H	-0.78377989008930	9.71439935371696	2.99032560074288
H	-1.17246568610649	11.09553139689449	1.96633963724982
H	-1.94764370963719	10.92153048784775	3.55368045814467
C	0.18919773454754	11.09370337199268	5.12052685875378
H	0.92807900250618	11.66466573076479	5.68322578977873
H	0.45558045399071	10.03849715605845	5.17440387328432
H	-0.78012457837142	11.22937356056648	5.60620445243549
C	4.56312024500596	10.57282307500035	0.79617653701460
C	5.31892588680833	11.01084126133797	1.88341480157863
H	4.95605159904397	11.83888172379276	2.47447926238227
C	6.52050598302202	10.40074589334772	2.20426868683985
H	7.08470994613327	10.75605999028975	3.05894198134787
C	6.99532609905901	9.34284347936965	1.44388778764362
H	7.93458600617510	8.86461851095593	1.69580712721829
C	6.25398190493521	8.89773778712255	0.35773546929634
H	6.61101381663667	8.06642514524686	-0.23965540466973

C	5.05104459722953	9.50529887105089	0.04005753395951
H	4.46224282587568	9.15127637328026	-0.79664111437927
C	3.50217457567124	12.29499558383803	-0.55926652652647
C	2.23110709579656	13.11980640061054	-0.63739785389815
H	1.94671807808452	13.49634944292427	0.34669665575740
H	1.40066643065277	12.53165201630028	-1.02364913688521
H	2.38881626665155	13.97268400600564	-1.30067459246171
C	3.79773908147954	11.69089059660102	-1.93193748213638
H	4.78534193877995	11.22730564679988	-1.95374712144541
H	3.77302688710816	12.46534363716534	-2.70301849298276
H	3.04988831078677	10.93545836735604	-2.17520561311711
C	3.44409569789263	6.40158113928162	0.96617769915577
H	3.75721696295373	6.11218346734224	1.96907478278413
H	4.20831261301221	7.04998332666424	0.53881044847473
H	3.38433259296375	5.49423740391196	0.36060563936780
C	-0.26175875643654	13.05359551898555	3.67847500485155
H	0.46145070629452	13.62542842218495	4.26188439816684
H	-1.24683529273550	13.20176479718103	4.12807647030027
H	-0.28202678830575	13.44913515804648	2.66579474203070
C	4.64499508347099	13.23478702035103	-0.17162692431418
H	5.60654493083353	12.72128643854354	-0.14895694172466
H	4.46936872208339	13.68012826350937	0.80923352995580
H	4.71129031425596	14.03928276940909	-0.90790756327362
N	-2.17349130564608	12.61360492526042	-1.71087992915549
C	-2.93155490657281	12.84293919645268	-2.89704089953172
C	-4.32146408666047	12.76159950740524	-2.89100952066991
H	-4.82702073815579	12.52275201053947	-1.96849093109441
C	-5.05603672436286	12.99135842092861	-4.04353040591239
H	-6.13629258312775	12.90957513258051	-4.00805081755247
C	-4.41802591649054	13.32231775207993	-5.22803766971801
H	-4.99107827559789	13.50029827090072	-6.13042736083404
C	-3.03310354985340	13.42555701175018	-5.24584274932718
H	-2.51821931173620	13.68592691235266	-6.16370026984305
C	-2.30150644591002	13.18833219036526	-4.09499123661425
H	-1.22427929863652	13.26319493518644	-4.11616358020780
C	-1.86877437002503	13.86018855364012	-0.96347299905937
C	-1.33761638240493	13.50748484424881	0.40881634664106
H	-2.06921154641072	12.91541222325410	0.96312798956527
H	-0.41309971174340	12.93552478407048	0.35197236260430
H	-1.15080346648552	14.42622801013950	0.96815282282237
C	-0.83732598373568	14.70460290793750	-1.70972249031559
H	-1.24615981498213	15.09573842110937	-2.64257998780985
H	-0.53479799493346	15.55722558659440	-1.09628605910608
H	0.04807841983051	14.11137353841622	-1.93707947739588
C	-3.13934671914777	14.69061114520252	-0.76581998219751
H	-3.54904427470916	15.04098968649325	-1.71345971773201
H	-3.90692198301346	14.10716066611718	-0.25420734457782
H	-2.90642039826641	15.56541703640631	-0.15434331284256
N	-1.39710583795004	9.90786770276861	-3.18971029415308
C	-2.52618378658307	9.20061011210392	-3.69344346021774
C	-2.69075303039414	7.84102340261373	-3.42871394132277
H	-1.96440350263479	7.34016519965504	-2.80121947313144
C	-3.77362447817615	7.14576499462798	-3.93936143774011
H	-3.88774744392737	6.09094564469652	-3.71671481826847
C	-4.71710000131781	7.79797307474058	-4.72164204627793
H	-5.56766494872280	7.25449757465151	-5.11591288890492
C	-4.56226508927445	9.14930429036405	-4.99010549668117
H	-5.29316754485621	9.67388331875752	-5.59464210427875
C	-3.47575757932336	9.84408466750692	-4.48476249978111
H	-3.35538497254436	10.89574933825736	-4.70023250701540
C	-0.17362437539834	9.81580445781253	-4.04197788332657
C	0.70729645973245	11.03135717386361	-3.80454845784124
H	0.19459675579555	11.94624907015612	-4.09656378718130
H	0.98255563914747	11.10999519433933	-2.75527088197640
H	1.62127793569000	10.94482926481525	-4.39582159042039
C	-0.54531933552477	9.78833610290954	-5.52314156240965
H	-1.11564148064589	8.89812196668552	-5.79084437693645
H	-1.12928523797813	10.66831964091285	-5.80023699620211

H	0.37469412880894	9.79016448021935	-6.11169099143941
C	0.60362787804994	8.55321491084342	-3.68755118234571
H	0.01698348155371	7.65692729678327	-3.89651740092823
H	1.52422074239236	8.49688924757039	-4.27426294912662
H	0.86432486562222	8.57085314935000	-2.62992778413146
N	-3.15702977848560	9.70731282076447	-0.40213235569890
C	-4.42679624834509	10.33201028468904	-0.56419589160665
C	-5.30290064072693	9.92692304404546	-1.57043081824224
H	-4.98875906980612	9.15884069163101	-2.26370150373888
C	-6.55468517813083	10.50782541354191	-1.69408477738819
H	-7.21606246528197	10.18323957005545	-2.48921169373601
C	-6.95368964097427	11.50648710096685	-0.81910271667408
H	-7.93090149431054	11.96402543739040	-0.91874569119894
C	-6.08571079654238	11.92482982263341	0.18166376759267
H	-6.38294798329473	12.71187388510006	0.86526830879839
C	-4.83727057367478	11.34022064904440	0.30891662265158
H	-4.15487994961240	11.66075316487819	1.08671569556456
C	-3.23739850559508	8.47066431594287	0.42934715907007
C	-1.87862267950034	7.81346867968555	0.50595604617616
H	-1.50186347883038	7.57681813598096	-0.49098823663529
H	-1.14833131421718	8.45713972857154	0.99462677189118
H	-1.96390862335056	6.88158114502769	1.06756030662944
C	-3.71495252585689	8.80342127237190	1.84148788347732
H	-4.73352166110175	9.19393029507116	1.83486169041546
H	-3.70828250312815	7.89900791838014	2.45494963184706
H	-3.06298272463642	9.53938648177207	2.30626411107744
C	-4.21472751270937	7.45829854463535	-0.17389828579209
H	-5.24067795103334	7.82587818683683	-0.16477107999648
H	-3.94486248621558	7.21062249547280	-1.19970232459674
H	-4.18475613774661	6.54282136568716	0.42163883776915

[(tBuPhN)$_3$W=P=W(NtBuPh)$_3$] (S = 1/2)

Eh = -3138.963176399952

W	1.77345835193423	10.34274113751822	1.32189763939373
W	-1.77307643968402	10.67043778328471	-1.32217781935091
P	0.00022617601594	10.50650961030468	-0.00020952339130
N	2.14352218726898	8.40570316147886	1.54621010433466
N	1.32585370220185	11.31653561624268	3.00015468594728
N	3.23314008184772	11.28138675162816	0.34941444185061
C	2.69347074476472	8.19661823924912	2.84918525208449
C	4.06999029204335	8.22892296387800	3.06500199211211
H	4.72760220212110	8.43580115325083	2.23299074372201
C	4.59491155383098	8.00161900000782	4.32756891669378
H	5.66834423384072	8.04279288997469	4.47182852060250
C	3.75551741397087	7.73294588759155	5.39692223412927
H	4.16526607320708	7.55696956808483	6.38460974020494
C	2.38130742753712	7.69967498866616	5.19524675434413
H	1.71464265670197	7.50012895390822	6.02644948763440
C	1.85757074022930	7.92884972063335	3.93495709199302
H	0.78690918871506	7.91362678217279	3.77286877992066
C	2.02422516171505	7.14922363992039	0.75607917918305
C	1.54595670667605	7.48062096621713	-0.64106681613514
H	2.23182029437255	8.17178911181098	-1.13605920490239
H	0.56040224018130	7.94357542048488	-0.62492361292478
H	1.49843616330629	6.56420762939085	-1.23314762078665
C	1.03491295410871	6.18966665555588	1.41343591570536
H	1.39378766129102	5.85198376325423	2.38709208319886
H	0.90304067287359	5.30675408562893	0.78311851440456
H	0.06537279496184	6.66729830676497	1.54378122648769
C	2.48968959454619	11.92310696745984	3.56544904072790
C	2.81925385304975	13.24156956320672	3.24859786526145
H	2.19986893889723	13.77171357243096	2.53615410076695
C	3.92121368588639	13.85476328758065	3.81844055768592
H	4.16387636984119	14.87786366440523	3.55480756168402
C	4.72027727372708	13.15839818130099	4.71687527497559
H	5.58579136416675	13.63702961410670	5.15985767334549

C	4.40676657794825	11.84617038652518	5.03264547986094
H	5.02807119060849	11.28692491599518	5.72269328498412
C	3.30009521588634	11.23452180699554	4.46412995529461
H	3.05721167077235	10.21181186881005	4.71386411441655
C	0.10846580263682	11.53691095192042	3.83357579723139
C	-1.05416021648314	10.75917017001234	3.25594541492903
H	-0.82510949333335	9.69240424835880	3.20926601888027
H	-1.29555147313184	11.09085522335366	2.24805503705687
H	-1.92918267870709	10.89385228053006	3.89551419712737
C	0.33486382336764	11.04178314048443	5.26364555037285
H	1.11181721594450	11.60998123099538	5.77545070388902
H	0.61359706465545	9.98779848326353	5.27439278897468
H	-0.59270045327350	11.15874985380843	5.82863324717111
C	4.49569586466136	10.68844595678670	0.67256884714379
C	5.26976961604213	11.14245809713363	1.73776407234450
H	4.90634577177939	11.95839695202447	2.34456744874964
C	6.49656845045396	10.56238660170463	2.02244038737031
H	7.07310767630398	10.92807678666915	2.86437061677733
C	6.98046793497707	9.52496745842509	1.24196299879089
H	7.93936386125379	9.07188118858939	1.46477421582564
C	6.22143547454088	9.06595267934694	0.17280804173260
H	6.58450373099051	8.24937195828059	-0.44095010873693
C	4.99403592455499	9.64096613510941	-0.10489563206412
H	4.39128579924769	9.27581617643374	-0.92683695611202
C	3.37101362644745	12.39256085442999	-0.63721841970258
C	2.03280551790811	13.07256798556581	-0.83378864651931
H	1.64092822679951	13.43419316611245	0.11924748555255
H	1.29049547455437	12.40096799375892	-1.26244866356399
H	2.16389904975201	13.92836223135069	-1.50007925870363
C	3.88818435484014	11.88566189441674	-1.98162387178925
H	4.91461606068164	11.52394618739170	-1.90479998982784
H	3.87621317491193	12.69774933271096	-2.71284438677460
H	3.26068227903045	11.07931708917181	-2.35587225804490
C	3.37671622714675	6.44353678708785	0.64000697812582
H	3.75057679598893	6.11447732077279	1.60991104918225
H	4.12364832094093	7.09520652457163	0.18601263850888
H	3.26208985986111	5.55950913197123	0.00862680875065
C	-0.25398595182452	13.01946333001614	3.89024856952328
H	0.51353691480036	13.59943926909377	4.40475897372874
H	-1.19076328321682	13.14950789702582	4.43772065554347
H	-0.38301071114650	13.42183700520188	2.88746271182616
C	4.35013804238449	13.44593771762012	-0.11131179718058
H	5.36045761911430	13.05010566257843	-0.00618802679807
H	4.02809539004705	13.82876707710788	0.85783086027958
H	4.39007034233324	14.28012535976975	-0.81556860815845
N	-2.14305046036545	12.60752510004932	-1.54617402093323
C	-2.69515681598338	12.81624653744014	-2.84829310426660
C	-4.07204402289763	12.78367561330767	-3.06176677167049
H	-4.72821727222771	12.57716849364773	-2.22852709053655
C	-4.59911762747033	13.01002044721755	-4.32360034623974
H	-5.67278789762654	12.96855443119815	-4.46601082875699
C	-3.76156763954858	13.27800763208592	-5.39456248791834
H	-4.17300679973226	13.45312390093881	-6.38169605168107
C	-2.38702453746471	13.31166101174955	-5.19520570760440
H	-1.72179574117923	13.51069993606071	-6.02767887244907
C	-1.86111439957328	13.08341397470232	-3.93564484186539
H	-0.79017301915794	13.09877901664806	-3.77539388577246
C	-2.02349230676532	13.86406019608994	-0.75610857824899
C	-1.54691128094252	13.53263150526839	0.64161634507938
H	-2.23386176462734	12.84218310282446	1.13610711509364
H	-0.56172069734654	13.06887980990158	0.62666967448085
H	-1.49927365124052	14.44917869931977	1.23348097046513
C	-1.03274760796929	14.82271862457961	-1.41265217016587
H	-1.39028676887866	15.16034237745774	-2.38681734487058
H	-0.90096405562253	15.70573905804234	-0.78246467543411
H	-0.06339061496888	14.34435902568177	-1.54174608994884
C	-3.37546839786946	14.57098923964032	-0.64162582787504
H	-3.74801419473023	14.90013843147315	-1.61200838798640

H	-4.12349399146250	13.92017437074862	-0.18822593626961
H	-3.26066806084204	15.45509745757098	-0.01039370953682
N	-1.32553448850301	9.69700178988272	-3.00072293519763
C	-2.48944769733295	9.09066877325074	-3.56614669894129
C	-2.81866539799387	7.77188507622415	-3.25023579412808
H	-2.19911055986712	7.24137003639499	-2.53821649543633
C	-3.92026234884367	7.15868558292832	-3.82077568645202
H	-4.16254244927573	6.13527463516903	-3.55799558957998
C	-4.71934252491422	7.85535635402533	-4.71895671036870
H	-5.58447964994643	7.37665495986853	-5.16260278975949
C	-4.40626811615213	9.16792986051609	-5.03371879251818
H	-5.02753587495200	9.72740485351028	-5.72361905876953
C	-3.29992122607792	9.77956651358886	-4.46455072659674
H	-3.05721653743553	10.80244700919192	-4.71373067473490
C	-0.10815173284113	9.47631528095986	-3.83412167071804
C	1.05488127059431	10.25312831871328	-3.25607817186342
H	0.82649979358744	11.32002638531653	-3.20913569060291
H	1.29591849387716	9.92099912465037	-2.24824932076067
H	1.92991271492011	10.11808382589561	-3.89555677361017
C	-0.33406168956297	9.97214013964486	-5.26403128889950
H	-1.11129518039225	9.40462521850942	-5.77616244101237
H	-0.61214547022966	11.02630089137445	-5.27440754757323
H	0.59349711311307	9.85482094180685	-5.82895645593415
C	0.25334333993794	7.99354028499824	-3.89132956233306
H	-0.51457093895623	7.41424771360232	-4.40604011397930
H	1.19002787196947	7.86307018648687	-4.43886400951565
H	0.38211450311305	7.59073062951759	-2.88868077928312
N	-3.23236566277202	9.73114475114329	-0.34971345025465
C	-4.49528724470745	10.32381768423615	-0.67190726552763
C	-5.26939960419572	9.87054604938587	-1.73739496167033
H	-4.90557701683421	9.05563366047163	-2.34535635018236
C	-6.49671095383836	10.45009147663802	-2.02095927949935
H	-7.07321255777971	10.08508362211825	-2.86321164976417
C	-6.98110503548857	11.48617870963066	-1.23902204318748
H	-7.94034118793998	11.93894033032970	-1.46102624357950
C	-6.22208407488092	11.94433786447868	-0.16949563321361
H	-6.58555796836652	12.75985052154266	0.44543714820489
C	-4.99414914988028	11.36992216576836	0.10704564174824
H	-4.39145085506111	11.73439746124624	0.92932092809165
C	-3.36946482541172	8.61938662112215	0.63632777012034
C	-2.03091657746671	7.93973523375351	0.83184435702399
H	-1.63944411400332	7.57863624367537	-0.12156245596376
H	-1.28858541378770	8.61140240061261	1.26036338193218
H	-2.16131166986454	7.08361818910549	1.49785889062769
C	-3.88611134372686	9.12541146332609	1.98126189403981
H	-4.91271960271223	9.48679315590425	1.90518605295238
H	-3.87345103081658	8.31297344019306	2.71207720916379
H	-3.25869110040484	9.93179293450217	2.35556948360645
C	-4.34849781585079	7.56600958353517	0.11030112608042
H	-5.35896025932072	7.96161992921923	0.00573130728239
H	-4.02669974839798	7.18372119205028	-0.85913402277896
H	-4.38795277703353	6.73148692965758	0.81418746135583

[(tBuPhN)$_3$W=P=W(NtBuPh)$_3$] (S = 3/2)

Eh = -3138.943140680591

W	1.83373603642603	10.37352171054917	1.34409816871855
W	-1.83443174189218	10.63840736416865	-1.34371783448396
P	-0.00059829462235	10.50560543543986	0.00054552038519
N	2.20105498055778	8.44480608852715	1.62085979214151
N	1.37309618286245	11.29624490207762	3.03833660348119
N	3.31012908681230	11.29008263332467	0.38868538226207
C	2.78277870992946	8.23072450149947	2.90771491932473
C	4.16380005468790	8.25118653152667	3.09345741862378
H	4.80541868010707	8.45662571352988	2.24929250548582
C	4.71293539287540	8.01250144985443	4.34220460969976
H	5.78968889961584	8.04474483103655	4.46247214571550

C	3.89571483797601	7.73971630033283	5.42959883319939
H	4.32623877874199	7.55409891398892	6.40652607163109
C	2.51833742215074	7.71396982095043	5.25627785786960
H	1.86766314185359	7.51047526078639	6.09922567715413
C	1.96879348832024	7.95770315129339	4.00889008328573
H	0.89463899522541	7.95260340804900	3.87249623320802
C	2.02629158249603	7.18532326266436	0.84004757716118
C	1.56892492290486	7.50797233976354	-0.56728044255289
H	2.26967042383513	8.18378518398095	-1.06328315903833
H	0.58935261779678	7.98282117285610	-0.57169441870408
H	1.51703835538092	6.58409816577260	-1.14716865569119
C	1.00010662283465	6.27120412741144	1.50516770284317
H	1.34502989061657	5.92922480027919	2.48200462143698
H	0.83335795526538	5.38874642832110	0.88244044303785
H	0.05015841571033	6.78828850101963	1.62989455370934
C	2.53397893379485	11.90097893765587	3.61028932830564
C	2.86060431633296	13.22267293654973	3.30036389404706
H	2.24064324497514	13.75839934064781	2.59257392785852
C	3.96274778765569	13.83559763993112	3.87192081312332
H	4.20175653589558	14.86074488356246	3.61252356323958
C	4.76378173699935	13.13842752753366	4.76631941171269
H	5.62868419970346	13.61636797088112	5.21098908537563
C	4.44953985212897	11.82370250505814	5.07838504204070
H	5.06970959348975	11.26307909005299	5.76831671010136
C	3.34478665379832	11.21178184634345	4.50990034487679
H	3.10389728179192	10.18894565783633	4.75897826032783
C	0.13719017689783	11.52182393983979	3.84402784387204
C	-1.01033253585181	10.71726887726991	3.26853508778460
H	-0.75898584653772	9.65516239418180	3.21734120239224
H	-1.26537786406155	11.04674792374072	2.26297143685698
H	-1.88511133371540	10.83115718744682	3.91219373896626
C	0.35477588236383	11.06322284228915	5.28711569079678
H	1.12337922708077	11.64944508250865	5.79084429814029
H	0.64249583084093	10.01196389768259	5.32525402298034
H	-0.57831192234083	11.18578260334190	5.84157892824866
C	4.56174769922957	10.67649425538430	0.69951744774975
C	5.34151422137796	11.11095382810528	1.77007437965245
H	4.97956078104216	11.91297424085250	2.39664035634528
C	6.56694233844503	10.52307593236721	2.03612881351734
H	7.14996034284396	10.87312483213066	2.88020447498051
C	7.04229358242266	9.49355052788804	1.23681342035599
H	8.00046965930493	9.03342631929618	1.44743516220988
C	6.27475341014797	9.05283406018223	0.16717550215208
H	6.63039088995397	8.24348178634910	-0.46036992124284
C	5.04842697465350	9.63859143831677	-0.09771491791760
H	4.44130669764513	9.29017824002088	-0.92395249219571
C	3.43685248429856	12.43102211890099	-0.56468149269118
C	2.10175029007176	13.13063693706238	-0.71652173885615
H	1.71777302607665	13.45820947442756	0.25249627316715
H	1.35465665696108	12.47958254509477	-1.16754311477527
H	2.23113738747594	14.01183962101658	-1.34817818406831
C	3.91018084869877	11.94858576323159	-1.93360431524525
H	4.92291911980848	11.54588993043217	-1.88638779257785
H	3.91589030800406	12.78435712088326	-2.63767292346672
H	3.24483633600008	11.17849372633485	-2.32086706941715
C	3.35536957659638	6.43391770536940	0.73998903837013
H	3.71116808266447	6.10127898110316	1.71511314672658
H	4.12565981838459	7.05897380312466	0.28658514429771
H	3.21556011053445	5.54988712775244	0.11397076369255
C	-0.24257556049849	13.00049639630741	3.85503652479284
H	0.51090616918695	13.60170063618252	4.36561302396258
H	-1.19026134236142	13.13520209853927	4.38236525463661
H	-0.35916451246855	13.37307780600778	2.83865282869750
C	4.44277005816620	13.45356597194606	-0.03253295069749
H	5.44797585656194	13.03849056541666	0.04051538878801
H	4.14767771253873	13.81582634808272	0.95287116368308
H	4.48075261329195	14.30449499853716	-0.71649674345864
N	-2.20147053130480	12.56727739392052	-1.62042891014755

C	-2.78119142888326	12.78199213500128	-2.90806342188456
C	-4.16197666531708	12.76296215056485	-3.09575748997198
H	-4.80499519678985	12.55809293667593	-2.25250823497408
C	-4.70907558682910	13.00226539792583	-4.34527091870420
H	-5.78568396631267	12.97106538989999	-4.46710191489502
C	-3.89002009573839	13.27414994724929	-5.43150894019984
H	-4.31895396133785	13.46004504490254	-6.40907823776003
C	-2.51286399401716	13.29857216446335	-5.25620746447860
H	-1.86078270761269	13.50136528676751	-6.09823598647598
C	-1.96534902866978	13.05431182961940	-4.00803792335323
H	-0.89138593643623	13.05831708133168	-3.87008822324406
C	-2.02679672787106	13.82661260829472	-0.83937354600857
C	-1.56839566241240	13.50381583271700	0.56757305783463
H	-2.26845355557028	12.82745690603755	1.06381080984867
H	-0.58854020625645	13.02953592191492	0.57127730732458
H	-1.51670850665414	14.42755429214536	1.14769345556515
C	-1.00151725902199	14.74141213915873	-1.50494031165683
H	-1.34720398046646	15.08330589153293	-2.48153701266475
H	-0.83496550680243	15.62388328194806	-0.88218259182080
H	-0.05133439811363	14.22492918462683	-1.63026623977194
C	-3.35619080444267	14.57730947481010	-0.73826613169172
H	-3.71276132745908	14.91007024690245	-1.71306641163961
H	-4.12589993943710	13.95175117501476	-0.28455632670168
H	-3.21641065267572	15.46122551643185	-0.11207789401451
N	-1.37345208026909	9.71549789534120	-3.03781418766372
C	-2.53445813021635	9.11187796513513	-3.61061941987999
C	-2.86263301119320	7.79049141428286	-3.30100019741354
H	-2.24351573056413	7.25397975984890	-2.59306411714635
C	-3.96552079253274	7.17903049831032	-3.87272558181373
H	-4.20594712385645	6.15420837544587	-3.61334227140985
C	-4.76571778581277	7.87739106277866	-4.76697031220945
H	-5.63143690596684	7.40076447811037	-5.21144902764672
C	-4.44967427108377	9.19168701968949	-5.07900393392523
H	-5.06913808577487	9.75322826758708	-5.76883076459006
C	-3.34415859338736	9.80208561302769	-4.51043092575357
H	-3.10195059830121	10.82463137462266	-4.75936110232394
C	-0.13718388208820	9.48890902598437	-3.84256387395085
C	1.01042265115197	10.29279472656155	-3.26633973709760
H	0.75970173968953	11.35507031522322	-3.21557799029263
H	1.26448295852914	9.96338214169396	-2.26050481377646
H	1.88563030337575	10.17821958559092	-3.90929182026272
C	-0.35333482059207	9.94747384141334	-5.28587842524117
H	-1.12199642755540	9.36176816623177	-5.79011559503119
H	-0.64024093461444	10.99895131270572	-5.32436163048922
H	0.58007958895547	9.82416502109010	-5.83962722974489
C	0.24144812597994	8.00993818107951	-3.85292824516122
H	-0.51197124279567	7.40923724585311	-4.36420044246572
H	1.18959177040900	7.87436964108211	-4.37920984547107
H	0.35663305580321	7.63744991510978	-2.83633756050582
N	-3.31117292445542	9.72213100551848	-0.38880399149426
C	-4.56236413788860	10.33654385982748	-0.69981470795761
C	-5.34224299881091	9.90250939135177	-1.77045143646195
H	-4.98093217966038	9.09991899601289	-2.39662754609275
C	-6.56700598896503	10.49145211916256	-2.03709229183526
H	-7.15004718928691	10.14163161925332	-2.88125880748584
C	-7.04160210079417	11.52170074266832	-1.23825341465752
H	-7.99929681409847	11.98265130314876	-1.44926463236211
C	-6.27390677112275	11.96210141100462	-0.16859404750077
H	-6.62891885219308	12.77205827790128	0.45852303575267
C	-5.04820357837447	11.37527025104368	0.09685084996409
H	-4.44088959649907	11.72353369009132	0.92300643466178
C	-3.43849502713453	8.58101812669940	0.56433082956934
C	-2.10375504762713	7.88063174911495	0.71568853218525
H	-1.72018585804806	7.55305238928240	-0.25348999170905
H	-1.35622181488340	8.53123788811926	1.16662596358876
H	-2.23341846582817	6.99939386663751	1.34723833600329
C	-3.91113257214707	9.06337925420454	1.93352932269437
H	-4.92368655212234	9.46658485845546	1.88676653261568

```
H    -3.91700768751062    8.22744388897913    2.63739955769310
H    -3.24526547896021    9.83305335792969    2.32074319014855
C    -4.44509011982296    7.55910250149963    0.03224035095821
H    -5.45010573418825    7.97469698618875   -0.04042692678706
H    -4.15045858514034    7.19691133833087   -0.95332768427956
H    -4.48330490206468    6.70803583390768    0.71602180342254
```

[(ArO)(nacnac)V=P=V(nacnac)(OAr)] (S = 1/2)

```
Eh = -5790.364462313758
V     3.47387348580362   15.73056247288726    7.12509329836030
P     3.53474784831889   15.58568272149672    4.85007592475748
O     2.04189426951645   16.46239678057785    7.94345036077303
N     3.83320503677104   14.00491283315836    8.13169752548161
N     5.17578412642655   16.53721067915707    7.92997116941069
C     4.35183308897200   14.03714552498658    9.35742238164477
C     5.04432833685813   15.12441834274387    9.87662553313203
H     5.35292149101859   15.04312222915038   10.90785461430594
C     5.49526661086576   16.25123352800547    9.19002149738691
C     3.38421583794394   12.71151540201230    7.71685957655438
C     2.04916370331657   12.33309660808006    7.89694898901912
C     1.68332300231866   11.02474970351728    7.60023416769760
H     0.65337924330954   10.71726808499870    7.73931462177572
C     2.61190356830142   10.11060046359166    7.14292387965669
H     2.31147034810809    9.09218642474121    6.92417428828900
C     3.92936618794978   10.49921491544411    6.96852546425747
H     4.65505700656994    9.77718675786826    6.61588434707139
C     4.34268559653846   11.79263990604139    7.25951841407128
C     1.01637752419351   13.30400903490952    8.41170443711161
H     1.54389937659912   14.18868981478748    8.76768793924004
C     0.10476730860239   13.74490138650262    7.27526500879341
H     0.68320005991724   14.16450604781349    6.44972792383671
H    -0.60038291766970   14.50536906695536    7.61660723527518
H    -0.46570565431700   12.89694971746735    6.88796253964155
C     0.20342920809289   12.74579573652621    9.57347173813841
H    -0.41179955727474   11.89681161549215    9.26578363299510
H    -0.47014545592515   13.51373238576984    9.96125421261473
H     0.84335301636182   12.41470177818300   10.39458111548294
C     5.79292797257962   12.18858947677054    7.10252091507851
H     5.98904852420299   13.01531681971253    7.78805866643448
C     6.04159265787326   12.68965985562801    5.68642480587549
H     5.81526811054317   11.90531606156251    4.96657796599687
H     7.08852315738444   12.97352569785162    5.55581538454208
H     5.41352706901992   13.55058335029326    5.44917015491340
C     6.76259539302387   11.06689838324355    7.45272447922720
H     6.55550014909194   10.63835151976222    8.43661430890390
H     7.78461947910113   11.45284905177465    7.45949877955033
H     6.72958450812909   10.25911830919438    6.71761556934493
C     4.23936398184669   12.83953401415243   10.25667685196879
H     3.21981848125090   12.45425354479585   10.28027098387877
H     4.55488888343780   13.09607804193310   11.26590844422452
H     4.87089790086613   12.02636138957649    9.89151536625172
C     6.39027480153845   17.17092208331221    9.97340192779318
H     7.33906982355484   17.34070126294912    9.46445199133021
H     6.58323014013058   16.76055438123114   10.96226144094237
H     5.91629806761016   18.14843823753698   10.08331483961219
C     5.96520090303325   17.59639133862203    7.37292753136713
C     5.59264212440724   18.93437145002633    7.54807327424846
C     6.49559584986424   19.92609175421611    7.18083745330135
H     6.22301211494396   20.96576561361927    7.31427459532765
C     7.73445178217014   19.60825670425015    6.66201168465187
H     8.43256558287083   20.39336768695079    6.39600734039270
C     8.07107760089809   18.28184578698553    6.45811464783453
H     9.03585205693823   18.04073243667588    6.03019795236558
C     7.19658390648252   17.25533385384716    6.79014370164082
C     4.24393893211683   19.31908172412186    8.10139456632904
H     3.84320903245958   18.45770745137268    8.63844145776969
```

C	3.28729082562967	19.62746762169967	6.95444818772669
H	3.66243392727459	20.46085702299831	6.35416800202718
H	2.30029011976248	19.88810108527526	7.33924152325633
H	3.18166045243657	18.76430320917779	6.29380069681510
C	4.29413790704997	20.48824112669708	9.07604493857390
H	5.02910976648088	20.32992570930657	9.86947562376126
H	3.31294517563499	20.61928179964240	9.53742100358392
H	4.54495982610079	21.42488644849956	8.57264137810295
C	7.58351265180810	15.81055438585107	6.55709554530576
H	6.65563950870739	15.27045265409882	6.35236518560007
C	8.49701630838112	15.64017646170493	5.35170569671756
H	8.09474586692445	16.12557570529680	4.46383060565804
H	8.61690450090187	14.57857398651443	5.12905561528435
H	9.49609510192024	16.04175151883364	5.54074234308673
C	8.25021567438549	15.16836630429899	7.77333718555594
H	9.13658783752267	15.73696922489368	8.06951503017453
H	8.57363118634585	14.15387686231946	7.52507179067603
H	7.58427164654726	15.09675810717610	8.63213872713005
C	1.27494023131903	17.43811400187005	8.46295078372217
C	1.49130167872123	17.86122344614992	9.78969453538545
C	0.75137827661779	18.92797865858591	10.27621520304210
H	0.92613097019097	19.27316729999653	11.28823068055205
C	-0.20436393999446	19.55857315129091	9.49702061421401
H	-0.76257720066657	20.40057998318032	9.88891990791261
C	-0.45685105453658	19.08041338439324	8.22660019735494
H	-1.22846464326076	19.54565798425876	7.62347065658998
C	0.25568680854475	18.01236209685801	7.69327504130854
C	2.45405795358553	17.11578615283672	10.67287664347471
H	3.23628707376800	16.71455962859471	10.03184589124259
C	3.12642265558214	17.97181577808865	11.73475532857235
H	3.59703095288553	18.85694865150038	11.30336759846170
H	3.89833447891809	17.38980191511211	12.24470467322703
H	2.41955308551859	18.30491830340688	12.49890288335566
C	1.75412938673194	15.92242129168401	11.31971011397742
H	0.98836738699782	16.26290270005758	12.02229276707534
H	2.47009376321708	15.30260312632366	11.86675019170727
H	1.26475941985234	15.29897809645312	10.57145002191614
C	-0.09902277280478	17.48946017968822	6.32666421473882
H	0.49856842792307	16.59390807103845	6.14600894305883
C	0.23663488098712	18.48870753443739	5.22903178998335
H	-0.32006280362992	19.42125019460205	5.36132369639214
H	-0.02224879032681	18.07863863970242	4.25417287522851
H	1.29967245410841	18.72565373691848	5.22428706957283
C	-1.57529936124213	17.10748604951475	6.27607763555240
H	-1.83040740968896	16.39289696842252	7.06169637752791
H	-1.82093771150536	16.66852350416728	5.30940407954066
H	-2.21471800199784	17.98398137565536	6.40846998978644
V	3.20352157323917	15.35058525392608	2.38344349747213
O	4.41393412931271	16.33231699704547	1.44263848641359
N	3.47595853556167	13.49841967777041	1.63799342565505
N	1.37089773407119	15.46526826298029	1.52412444389194
C	2.98608014389246	13.18057605198251	0.44232178675197
C	1.98880879251093	13.90584049361545	-0.20471822821261
H	1.74338733149916	13.58032949457795	-1.20433858010375
C	1.19012787735119	14.91693929477265	0.31808997695468
C	4.32468412426666	12.49378717139914	2.20963844903399
C	3.71117526475971	11.40504278245547	2.84930073838605
C	4.50679591258803	10.33870978407216	3.24300380873332
H	4.05137816055589	9.48411303416534	3.72634202824044
C	5.87302654455641	10.34373265949107	3.01486045981474
H	6.47725880757985	9.49686652565671	3.31953804140488
C	6.46395408301478	11.43697677382238	2.41370365419977
H	7.53520793371087	11.44546260595158	2.25044844745634
C	5.70617610363899	12.52713366056304	1.99886332814370
C	2.22160303396031	11.40406722697103	3.09771777971025
H	1.73867389329124	11.89628076135248	2.24878264642637
C	1.61854078424141	10.01242934559829	3.22209960708373
H	1.87726503595969	9.37645938123530	2.37199079949174

H	0.52992642712656	10.08820292752541	3.27072340279840
H	1.94726459904895	9.51401971171396	4.13682855734107
C	1.92353687845029	12.22068759840328	4.34861842051210
H	2.44814524589914	11.80125802519182	5.20436234902391
H	0.85541078470568	12.22067549907279	4.57139242503988
H	2.25632565059323	13.25754687224330	4.25108998143629
C	6.37767929766756	13.70157302482517	1.33317458160129
H	5.59651256516049	14.33959725570555	0.92193630114132
C	7.30032444372574	13.29213135030061	0.19120433379982
H	8.14976550015672	12.70516605805617	0.54863512746737
H	7.70138828643667	14.18035491651603	-0.30274789174543
H	6.77623302294000	12.69806382802792	-0.56107673697247
C	7.12825650011397	14.52252778516462	2.37364481514787
H	6.47021045067297	14.80704529816252	3.19725988327318
H	7.53075558511157	15.43455558579591	1.92690615555847
H	7.95951257365682	13.94922675183679	2.79205841566663
C	3.47977836084680	11.95862695115573	-0.27685389157719
H	4.56914801041985	11.93461981800718	-0.31179382674797
H	3.08345009821786	11.93195807807531	-1.28968877719817
H	3.16529669826337	11.05206068960647	0.24518612604162
C	0.08012728929768	15.40021329127960	-0.57264248814217
H	-0.89012775777089	15.35019024347019	-0.07969799126813
H	0.04705338739163	14.81356790684407	-1.48818070705751
H	0.24535467330221	16.44813492751527	-0.83312527625561
C	0.25475332710451	16.26301923863159	1.95257857860944
C	0.17711059952454	17.62046024981823	1.61959687586580
C	-1.01537407061875	18.29392046268103	1.85810635963413
H	-1.09089124375834	19.34299482464592	1.60009078328288
C	-2.10439545718289	17.64758205822173	2.40712360929863
H	-3.03061025982305	18.18471228425805	2.57439418052631
C	-2.00058087585832	16.31542591687103	2.76397414648383
H	-2.85254561124041	15.81905148780642	3.21119287119431
C	-0.82671199267777	15.60223530634389	2.55784022870318
C	1.34974841879736	18.36089233755301	1.03186943597017
H	2.02317049149646	17.62537050025129	0.58859824241451
C	2.11007232738283	19.07109453541024	2.14615335542207
H	1.46501080814960	19.80098272008842	2.64239340114453
H	2.98627850812116	19.58513967121628	1.74937062448506
H	2.44146901269649	18.36203162882609	2.90869716744343
C	0.96762270432882	19.34838795357028	-0.06242991951348
H	0.34504114450351	18.88582771525467	-0.83269448298756
H	1.87369458949992	19.73208411639376	-0.53548739590598
H	0.42005103391408	20.20474060867625	0.33834017141928
C	-0.74908170637153	14.13916208361028	2.93904185717085
H	0.30153421768702	13.92278987081916	3.14183565096495
C	-1.54333105795918	13.82511160386085	4.19905279567980
H	-1.28655907571851	14.49467731476907	5.01825333206172
H	-1.33804194958289	12.80176945498006	4.51971165272836
H	-2.62032468498632	13.89305038010801	4.02599392183390
C	-1.20691718193853	13.20790391077391	1.81666911529997
H	-2.23385793306028	13.43874961848008	1.51938019748636
H	-1.18379109068515	12.17114100736589	2.16308292896713
H	-0.57121170965461	13.27220337416360	0.93490780190747
C	4.74315155640766	17.48226340153375	0.82153566278083
C	4.38249852239505	17.67759068304152	-0.52635129740741
C	4.67586843576045	18.89251831719684	-1.12636908994279
H	4.38281657481491	19.05670380028710	-2.15630638343266
C	5.33466809424210	19.89651462379086	-0.43640371139017
H	5.54055822339290	20.84693638741797	-0.91423035863822
C	5.75646388977910	19.65567388358628	0.85587188845026
H	6.30822761708139	20.42197443056887	1.38828898403809
C	5.49360284162813	18.45076304046432	1.49860075137109
C	3.75562241174107	16.55391368597299	-1.30600058433825
H	3.13652988109056	15.99071265564205	-0.61084331040927
C	2.86951256118350	17.00433830273364	-2.45649658605657
H	2.12748129596932	17.73712218639453	-2.13410854310255
H	2.34142507518850	16.14484251349768	-2.87700169395450
H	3.45252329226438	17.45017688707663	-3.26639977208876

```
C     4.83598185541246   15.59965295974528   -1.81051704908939
H     5.48201508521488   16.10548380864453   -2.53317380347933
H     4.38973202328408   14.72963439311856   -2.30091144830004
H     5.46207903854780   15.24691009913702   -0.99132527538876
C     6.03934151234868   18.20837113020951    2.88034625502068
H     5.88346338964786   17.15407379265969    3.12059772491159
C     5.30844936297856   19.02842185336757    3.93298580464827
H     5.38736952814138   20.09938338829379    3.72381360854829
H     5.73274393475656   18.84307122237190    4.91762480160250
H     4.25138561296284   18.76667583507604    3.97069518041335
C     7.53918290756055   18.48432035485715    2.91422521089405
H     8.06876658102993   17.88842642210359    2.16716387496137
H     7.94317978735845   18.25295710766742    3.89886554598047
H     7.75683129023950   19.53655358027248    2.71570083858633
```

[(ArO)(nacnac)V=P=V(nacnac)(OAr)] (S = 3/2)

```
Eh = -5790.37105693426
V     3.59682303530985   15.76517000358779    7.11639153351048
P     3.42430775801706   15.48943197305554    4.75659655382091
O     2.15367130225771   16.40729059930168    8.02351950227331
N     3.92016827377756   13.97316020726892    8.02612171365475
N     5.29244491569833   16.52015558808083    7.90640121556622
C     4.43032285857184   13.97620161700986    9.25182295820026
C     5.12281843103469   15.05388862899658    9.80826395205982
H     5.41131675592749   14.94234902543874   10.84250438821893
C     5.58498121424192   16.19702989401873    9.17218998971667
C     3.45866462918830   12.68787011385887    7.59253316771409
C     2.12830235573664   12.30819934004098    7.79589747051691
C     1.76429377358793   10.99631229858724    7.51177327053946
H     0.73826722114356   10.68492127464571    7.66995709600470
C     2.68710140362988   10.08596657466017    7.03669081978474
H     2.38662503408048    9.06616927611305    6.82493307273410
C     3.99839340552023   10.48038566600180    6.83033437197300
H     4.71856121817434    9.76115353325912    6.46252488705810
C     4.41201384875113   11.77490551366013    7.11446943131764
C     1.09703599591124   13.27343345706590    8.32375712073083
H     1.61506470030201   14.17941586252829    8.63587756105101
C     0.13436055831442   13.66456319293599    7.21147448815290
H     0.67254291349984   14.07672536895688    6.35548595782851
H    -0.57543247993358   14.41578120524593    7.56343105539769
H    -0.43144806079277   12.79561703823752    6.86686377395855
C     0.33992089430940   12.73010823110252    9.53025961769497
H    -0.26107831997528   11.85511255643367    9.27107652449620
H    -0.34003821518279   13.49248081762295    9.91780871207697
H     1.01599169333961   12.44328471888534   10.33897457093516
C     5.85641890681362   12.18384195214866    6.94289066824313
H     6.09070499001794   12.91306233837632    7.72287896816234
C     6.04501151288938   12.86746050523600    5.59630494413920
H     5.75922012668596   12.19449484296858    4.79104749027577
H     7.09010427490127   13.14730404226528    5.44663033478339
H     5.43053361127248   13.76483640547496    5.50535085596544
C     6.83809390860931   11.03019546085118    7.09466674455310
H     6.67529582684848   10.47248433207522    8.02040171595144
H     7.85912617304616   11.41825719360128    7.10739658266650
H     6.77272973219437   10.32897910917060    6.25887198399604
C     4.32314340058980   12.75798069084643   10.12423923313950
H     3.30541288648760   12.36880586017644   10.14005961999159
H     4.63851581804485   12.99311218423589   11.13856113349608
H     4.95719842330130   11.95513322815442    9.74074518447478
C     6.44442376237481   17.11007336405157    9.99943077698683
H     7.41452396019424   17.28338662887727    9.53395829231895
H     6.59338747780759   16.69418104391207   10.99354803070551
H     5.96437837300823   18.08701474052422   10.09139010740307
C     6.10225313943541   17.58732393983272    7.38920351713374
C     5.69472727768766   18.91880413245327    7.52701186787085
C     6.58645893548336   19.92691643469593    7.17753781797463
```

H	6.28327236442631	20.96159525589541	7.28116885684843
C	7.85162009957082	19.62936334333561	6.71288496388242
H	8.54074695478505	20.42641319062135	6.45914433107006
C	8.22446033762224	18.30864548830463	6.54188810205681
H	9.20274816976083	18.08328993681183	6.13715594207677
C	7.36118334101158	17.26750114029590	6.85573082113948
C	4.30797103673823	19.27416495617218	7.99446083914732
H	3.90152428368938	18.41248695272489	8.52569336267464
C	3.42201925649477	19.53280979242061	6.77987386850020
H	3.78154704256327	20.40571416742618	6.22838163034066
H	2.38989019002070	19.71172480620245	7.08226050824024
H	3.43932661428675	18.68519204073212	6.08714104051572
C	4.26257656730626	20.45819249837304	8.95048468439882
H	4.94846539062496	20.32494924726018	9.79064831659959
H	3.25100789754883	20.56751169441599	9.34640806463738
H	4.52423100392982	21.39397504664007	8.45109612187002
C	7.77474200422580	15.83405933901934	6.60556151876697
H	6.85924681106218	15.28517392593087	6.37339976280189
C	8.71164042998762	15.70526628846940	5.41081917017513
H	8.33244683694228	16.23688965943059	4.53810280339941
H	8.82651070821242	14.65271929036224	5.14418921871818
H	9.70991534471026	16.08720785389877	5.64007003647563
C	8.42641441135727	15.17508106599411	7.82068388941436
H	9.29845273348408	15.74982183895227	8.14572544193250
H	8.76873764118816	14.17005367546709	7.55944603640886
H	7.74431894753798	15.07780593039108	8.66441429727094
C	1.41314024645966	17.41638386100209	8.51833475811974
C	1.65971535345147	17.88088679226651	9.82629279942970
C	0.94143822022375	18.97125922940121	10.29264545995312
H	1.13995814679051	19.34761960055734	11.28873710443208
C	-0.02102970091609	19.58953177033576	9.51163558067832
H	-0.56009375591526	20.45169920840515	9.88592000916767
C	-0.30765419992988	19.06932485725651	8.26552965796907
H	-1.08950343947918	19.52053891162035	7.66497335409234
C	0.37695914184603	17.97090786092881	7.75669819777843
C	2.64073345566974	17.15552304399907	10.70627035644716
H	3.46008211582375	16.82593758477881	10.06984815410864
C	3.23352018139836	18.00350132058505	11.81976904040073
H	3.65713067841177	18.93404400809764	11.43663887237987
H	4.02874083478734	17.44949883430424	12.32500423340027
H	2.48868105823813	18.25808010074118	12.57817311912266
C	2.00009070079539	15.89183189846334	11.27594998329095
H	1.14988937041482	16.14683694401782	11.91424451094151
H	2.72070454713675	15.32767898866737	11.87468947224404
H	1.64116442515483	15.24347769060288	10.47645297393477
C	-0.03480599424401	17.38443426639130	6.43233871246465
H	0.52584472930253	16.45851606565205	6.28953394974944
C	0.29573856909453	18.30278923434664	5.26558735782607
H	-0.22139950671039	19.26252471469290	5.35658776707149
H	-0.01200346109581	17.84508706096473	4.32669969363181
H	1.36563799026686	18.50162021215106	5.21116419046905
C	-1.52251535147836	17.04757189943716	6.44794857078764
H	-1.77143584403478	16.38095221724632	7.27660562202906
H	-1.81356957996714	16.57105682443392	5.51250465439938
H	-2.13207949490552	17.94821636310196	6.55523521975219
V	2.92279811085946	15.49662323031820	2.47607273864144
O	3.78208295114221	16.74999580323457	1.47943246005523
N	3.31226993614959	13.64764223943722	1.69496118246394
N	1.09300804872809	15.52222914664964	1.59813879393611
C	2.84507281885279	13.35092210655650	0.48589697019186
C	1.81073901507877	14.03885582911663	-0.15080319515438
H	1.59396312968279	13.72402718355199	-1.16027999775669
C	0.95556478929044	14.99544818118752	0.37998571804171
C	4.22321432533835	12.66944860700712	2.21236540749255
C	3.68126006722116	11.50401555126018	2.77944729226683
C	4.54510641040305	10.47366132706759	3.12418414338837
H	4.14444698575715	9.56516168541456	3.55443802012378
C	5.90787153108780	10.57821555806122	2.90197394301732

H	6.56605491650026	9.75683160124334	3.16154241453296
C	6.42515531193155	11.73671145652086	2.35831608742761
H	7.49291627073116	11.82014356720674	2.19440801862302
C	5.60056233224509	12.80072810185699	2.00690794876716
C	2.18971579447088	11.36989039326712	2.98150579856731
H	1.69370436490634	11.84856643428206	2.13229540579168
C	1.70485861899774	9.92838662738459	3.03709494986350
H	2.04218336030360	9.34787200915393	2.17484185256493
H	0.61272065243126	9.90966232034149	3.05202539042767
H	2.04766248698521	9.42487660660267	3.94435249832029
C	1.77734607297869	12.11361951372099	4.24396877668637
H	2.30669873673069	11.71058938521950	5.10448283410792
H	0.70575983799766	12.00716744618523	4.42610062285689
H	2.01591236617206	13.17695658568557	4.18499942276965
C	6.20705178848713	14.04630236974060	1.41128918266992
H	5.39936192516580	14.64692170649558	0.99125017970494
C	7.20077913154801	13.75206712773712	0.29449342384394
H	8.09288945935124	13.24965579073084	0.67609103331596
H	7.52191405639597	14.68959811219632	-0.16486354036504
H	6.76888157344864	13.11736189927381	-0.48278964684641
C	6.87613587331091	14.86904237196390	2.50517418233339
H	6.17657126715261	15.07788417334861	3.31705902095482
H	7.23728641422377	15.81841584632712	2.10347595480484
H	7.72613673803746	14.32251571601336	2.92098057431825
C	3.43055665114852	12.20576318921799	-0.29217260727656
H	4.51611869266859	12.29355420416537	-0.35480080279677
H	3.01382362188975	12.18040074318313	-1.29665343933661
H	3.22329384468857	11.25240705558979	0.19793925654547
C	-0.18068517087516	15.42277214441547	-0.50437364742083
H	-1.13965889576348	15.35673317943674	0.00869860518394
H	-0.21279161409435	14.81187570488356	-1.40406512924443
H	-0.04923002773097	16.46636453768802	-0.79526849447396
C	-0.03354296180209	16.28900140154967	2.04243856181347
C	-0.15174967702697	17.64665992978416	1.71855996866198
C	-1.33636912387958	18.30355112442559	2.03520762398696
H	-1.43866538428289	19.35429315333083	1.79071864763324
C	-2.38045913531767	17.64133737831508	2.64634599653991
H	-3.30036309883834	18.16564632508458	2.87721645178868
C	-2.24045857593841	16.30578310612212	2.98181192081521
H	-3.05754434781831	15.79660347233550	3.47663813324030
C	-1.07276658913042	15.60900759938930	2.70241351852672
C	0.96070069221715	18.41849374881975	1.05210887260070
H	1.71808702350204	17.70115793067747	0.73635471239658
C	1.62329416711226	19.36093292794998	2.05161288565699
H	0.91245213466750	20.10578671209054	2.41963320866492
H	2.45764121563122	19.88497721648373	1.58204389136844
H	2.00707504129558	18.81544201453952	2.91333977135400
C	0.49535344805985	19.20105498695104	-0.17172986708940
H	-0.01791852002452	18.56825824776760	-0.89884191363150
H	1.35381151943605	19.65746348486956	-0.66989783114950
H	-0.18928155938473	20.00671339046102	0.10407327305548
C	-0.94280430481086	14.14753858116548	3.07575423400616
H	0.11017762470237	13.97954383334974	3.31231250369498
C	-1.76499766340239	13.77494276579666	4.30107087955070
H	-1.57333514623553	14.43970669085102	5.14203611432155
H	-1.51759218548054	12.75818125581479	4.61236692323845
H	-2.83698002937825	13.79012613276922	4.08747887919257
C	-1.31827380255310	13.20504638376896	1.93148861572870
H	-2.34406266956217	13.39164944393588	1.60094121818763
H	-1.26047590650396	12.16827952888982	2.27402088971804
H	-0.65642577315948	13.30122922582738	1.07284072896109
C	4.82677682408079	17.34519300442797	0.87864243453496
C	5.15964330858741	17.00157301327739	-0.44709822850473
C	6.26226881013558	17.60477757299347	-1.03323190431964
H	6.53501032644922	17.33343649150613	-2.04612459357021
C	7.01322376107097	18.55110704047658	-0.35634327606330
H	7.87630474655844	19.00538356204279	-0.82853587999580
C	6.63247599825169	18.92680394409448	0.91725212173544

H	7.19820509284167	19.68915544787047	1.44116998180261
C	5.53882506371866	18.34862600905084	1.55028565473636
C	4.28027285315054	16.06605753540714	-1.23334891341881
H	3.83843536423561	15.36552241668509	-0.52724189848779
C	3.12884870283907	16.84343912348139	-1.86818493687130
H	2.55786804369265	17.38953167267823	-1.11779006026750
H	2.44705705648421	16.16746238691387	-2.39167320520938
H	3.51125885314705	17.56958176111627	-2.59090703575339
C	5.00699265657852	15.25865572393523	-2.29784550664463
H	5.34172116498478	15.88693867119229	-3.12755015554404
H	4.33081642032679	14.50875206016776	-2.71671124018238
H	5.87819426782894	14.74328127714741	-1.89356709631236
C	5.15172625999539	18.78854444730511	2.93584118800230
H	4.16142307065350	18.38100654290643	3.14480557758738
C	5.07786352975418	20.30638683432396	3.06298016957892
H	6.06897562724413	20.76290328512698	2.99944922359849
H	4.65786271882899	20.57814834692197	4.03392994738130
H	4.45489552337047	20.74941430861888	2.28388059771205
C	6.11030052457272	18.21789682391159	3.96550802083921
H	6.05910004493142	17.13018855153985	3.98863424461187
H	5.86288114528054	18.58663167513588	4.95875025880317
H	7.13841676846996	18.51550731014656	3.74436744080114

OCPPCO (S = 0)

Eh = -908.94309736012

C	-0.30550459916729	-0.15465181787131	1.36051511206267
O	-1.31191731034545	-0.21006162202636	0.80874673709899
P	1.20014516510178	-0.13951835467465	2.12310633789396
P	0.57443295990641	1.75779173882980	3.14166272826101
C	-0.08119641287517	0.80788387295854	4.37329667633552
O	-0.53736980198415	0.21070562435126	5.24255732645339

CO (S = 0)

Eh = -113.229132257373

C	0.00000000000000	0.00000000000000	-0.15804232202835
O	0.00000000000000	0.00000000000000	-1.28195767797165

P_4 (S = 0)

Eh = -1365.006160439567

P	-0.00011270595724	-0.00000004987753	1.34318733659499
P	1.26625141915039	-0.00000004950167	-0.44816730030498
P	-0.63356941386212	1.09695275919307	-0.44801005896124
P	-0.63356929933103	-1.09695265981387	-0.44800997732877

[(pyrNdipp)$_2$VP] (2) (S = 0)

Eh = -2823.810550188603

V	8.04024792063550	7.70488444578031	12.26418554344962
N	8.92573227121109	6.99061646824763	10.53303146513980
N	6.38438455761410	7.27906849760781	11.12235366086461
C	6.70944049247139	6.77798791210286	9.88120268104465
C	8.07304909795831	6.61105596620013	9.62347470727302
H	8.42664417041440	6.17545731174213	8.69095230504537
C	10.31011878447652	6.74075051993319	10.34399127898895
C	9.95574633471160	4.47569196368778	11.42795029798575
H	9.05440498137830	4.98684824358583	11.77504772581109
C	4.49940572831082	7.04163138256539	9.91200391792812
H	3.45067062174903	7.06045283969605	9.65982679058742
C	5.55338099412856	6.61817683128474	9.11051476200413
H	5.50699886053783	6.24640824984940	8.09807738437849
C	10.83750407589412	5.51987671888761	10.78374715549762
C	5.05628779890813	7.44371061764059	11.13135977351712
H	4.54429856840815	7.82147393743949	12.00350564880937

C	11.11361668375841	7.73784809216604	9.78502704730488
C	10.53012625282152	9.05468055772057	9.33394952095524
H	9.51648535731028	9.11846760514690	9.73496566863350
C	12.19681113757437	5.29943929961472	10.61196649098903
H	12.63075270769232	4.36371684511907	10.94300183776113
C	9.52224520651792	3.42198233339220	10.41256400094898
H	8.97762879868064	3.86718204036499	9.57723714900966
H	8.87151732658333	2.68038016159100	10.88325736311781
H	10.39103418986761	2.89958450961929	10.00346134498948
C	13.00934112743854	6.26438388447078	10.04137347322148
H	14.06954600159129	6.07568460419373	9.91887569630270
C	12.47089037577975	7.47241309777825	9.63789770127080
H	13.11492466972025	8.22653827467510	9.20065674941026
C	10.59754260959167	3.83036885080812	12.64890489771118
H	11.45796076915385	3.21318022614636	12.37843666144006
H	9.87456402925877	3.17888519457425	13.14566841914784
H	10.92616536266792	4.58454786890915	13.36534254631223
C	11.30517104317300	10.24557954470941	9.88433770371951
H	11.34973836742709	10.21309008465178	10.97480802810698
H	10.81306643497696	11.17755608715989	9.59737982578742
H	12.32792200621475	10.27964266506273	9.50049091962939
C	10.43767976057768	9.10983802365535	7.81137609853007
H	11.42939198027724	9.04825387497669	7.35463896891201
H	9.97430236744439	10.04567020352480	7.48960268586569
H	9.84063781387110	8.28333307351660	7.41892481162805
P	8.03756169413290	9.70223873142051	12.25981928220542
N	7.15479541992818	6.99024685641832	13.99673582529802
N	9.69553725100825	7.28608416161645	13.40812768597115
C	9.37163385428561	6.78365677073401	14.64901161009213
C	8.00831387778825	6.61298338515329	14.90635251524759
H	7.65574171954133	6.17626485435753	15.83877122351479
C	5.77119694318847	6.73657514848556	14.18594390180649
C	6.13172367143849	4.47091789629942	13.10504816942935
H	7.03180809428566	4.98406299295153	12.75761339675059
C	11.58079310742987	7.05486721649104	14.61910962699368
H	12.62931904863397	7.07723892464345	14.87188189143874
C	10.52784714402479	6.62771399205339	15.42012743793128
H	10.57515002674329	6.25601378307364	16.43254690145135
C	5.24735211498784	5.51336048850941	13.74855657203223
C	11.02312857234033	7.45534087005861	13.39968447909995
H	11.53400517337301	7.83511907388203	12.52774816291129
C	4.96478976127040	7.73246691888428	14.74284957422391
C	5.54407095080350	9.05254504279764	15.18998716667519
H	6.55814732825223	9.11772033276421	14.79032364369113
C	3.88880718054171	5.28903108234927	13.92154055570944
H	3.45759533256550	4.35130369512238	13.59259044364144
C	6.56767615380278	3.41861052859600	14.12078103188872
H	7.11131921045427	3.86535488555577	14.95593014725866
H	7.22005656609813	2.67834332157360	13.65029235191452
H	5.70015252196458	2.89430444891778	14.53014140419021
C	3.07355990423250	6.25253051644283	14.49068563091707
H	2.01404467351987	6.06070263644634	14.61426605619534
C	3.60841013586098	7.46315845588054	14.89119108866223
H	2.96221072995260	8.21627793584545	15.32700126171625
C	5.49145657304764	3.82361117667991	11.88427948159902
H	4.63182628223842	3.20539273592463	12.15489243266675
H	6.21565199698105	3.17281845068698	11.38836903976886
H	5.16196208357750	4.57664167256757	11.16704054345395
C	4.76641501038526	10.23901831952458	14.63370982800077
H	4.72413009847748	10.20241727514922	13.54328012291455
H	5.25499747892993	11.17361215675109	14.91818970365259
H	3.74279320881717	10.27114808815745	15.01538828321276
C	5.63421324061660	9.11375655027388	16.71242761546616
H	4.64213157347733	9.05129258864750	17.16822832452365
H	6.09471576398413	10.05200251247813	17.03128754530724
H	6.23294588823903	8.29027461603546	17.10865133154409

11. References

(1) Manxzer, L. E.; Deaton, J.; Sharp, P.; Schrock, R. R. *Tetrahydrofuran Complexes of Selected Early Transition Metals.* in *Inorganic Syntheses, Vol. 21*, Fackler, J. P., Ed.; Wiley, **1982**, pp. 135–140.

(2) Heift, D.; Benkő, Z.; Grützmacher, H. Coulomb Repulsion versus Cycloaddition: Formation of Anionic Four-Membered Rings from Sodium Phosphaethynolate, Na(OCP). *Dalton Trans.* **2014**, *43*, 831–840.

(3) Ottmers, D. M.; Rase, H. F. Potassium Graphites Prepared by Mixed-Reaction Technique. *Carbon* **1966**, *4*, 125–127.

(4) Mu, J. S.; Wang, Y. X.; Li, B. X.; Li, Y. S. Synthesis of Vanadium(III) Complexes Bearing Iminopyrrolyl Ligands and Their Role as Thermal Robust Ethylene (Co)Polymerization Catalysts. *Dalton Trans.* **2011**, *40*, 3490–3497.

(5) Bain, G. A. & Berry, J. F. Diamagnetic Corrections and Pascal's Constants. *J. Chem. Educ.* **2008**, *85*, 532-536.

(6) *CrysAlis PRO*, Agilent Technologies Ltd, **2014**, Yarnton, Oxfordshire, England.

(7) Sheldrick, G. M. SHELXT – Integrated Space-Group and Crystal-Structure Determination. *Acta Crystallogr., Sect. A* **2015**, *71*, 3–8.

(8) Sheldrick, G. M. Crystal Structure Refinement with SHELXL. *Acta Crystallogr., Sect. C* **2015**, *71*, 3–8.

(9) Dolomanov, O. V.; Bourhis, L. J.; Gildea, R. J.; Howard, J. A. K.; Puschmann, H. OLEX2: A Complete Structure Solution, Refinement and Analysis Program. *J. Appl. Crystallogr.* **2009**, *42*, 339–341.

(10) Van Der Sluis, P.; Spek, A. L. BYPASS: An Effective Method for the Refinement of Crystal Structures Containing Disordered Solvent Regions. *Acta Crystallogr., Sect. A* **1990**, *46*, 194–201.

(11) Rogalev, A.; Wilhelm, F. Magnetic Circular Dichroism in the Hard X-Ray Range. *Phys. Met. Metallogr.* **2015**, *116*, 1285–1336.

(12) Perlepe, P.; Oyarzabal, I.; Voigt, L.; Kubus, M.; Woodruff, D. N.; Reyes-Lillo, S. E.; Aubrey, M. L.; Négrier, P.; Rouzières, M.; Wilhelm, F.; Rogalev, A.; Neaton, J. B.; Long, J. R.; Mathonière, C.; Vignolle, B.; Pedersen, K. S.; Clérac, R. From an Antiferromagnetic Insulator to a Strongly Correlated Metal in Square-Lattice $MCl_2(Pyrazine)_2$ Coordination Solids. *Nat. Commun.* **2022**, *13*, 5766.

(13) Rees, J. A.; Wandzilak, A.; Maganas, D.; Wurster, N. I. C.; Hugenbruch, S.; Kowalska, J. K.; Pollock, C. J.; Lima, F. A.; Finkelstein, K. D.; DeBeer, S. Experimental and Theoretical Correlations between Vanadium K-Edge X-Ray Absorption and K*b* Emission Spectra. *J. Biol. Inorg. Chem.* **2016**, *21*, 793–805.

(14) P. L. W. Tregenna-Piggott, D. Sheptyakov, L. Keller, S. I. Klokishner, S. M. Ostrovsky, A. V. Palii, O. S. Reu, J. Bendix, T. Brock-Nannestad, K. Pedersen, H. Weihe, H. Mutka. Single-Ion Anisotropy and Exchange Interactions in the Cyano-Bridged Trimers $Mn^{III}{}_2M^{III}(CN)_6$ (M^{III} = Co, Cr, Fe) Species Incorporating $[Mn(5\text{-}Brsalen)]^+$ Units: An Inelastic Neutron Scattering and Magnetic Susceptibility Study *Inorg. Chem.* **2009**, *48*, 128.

(15) R. T. Azuah, L. R. Kneller, Y. Qiu, P. L. W. Tregenna-Piggott, C. M. Brown, J. R. D. Copley, R.M. Dimeo, DAVE: A Comprehensive Software Suite for the Reduction, Visualization, and Analysis of Low Energy Neutron Spectroscopic Data *J. Res. Natl. Inst. Stan. Technol.* **2009**, *114*, 341.

(16) Neese, F. The ORCA Program System. Wiley Interdiscip. *Rev. Comput. Mol. Sci.* **2012**, 2, 73–78.

(17) Neese, F. Software Update: The ORCA Program System, Version 4.0. *WIREs* Comput. *Mol. Sci.* **2018**, 8, e1327.

(18) Neese, F. Software Update: The ORCA Program System—Version 5.0. *WIREs Comput. Mol. Sci.* **2022**, 12, e1606.

(19) Adamo, C.; Barone, V. Toward Reliable Density Functional Methods without Adjustable Parameters: The PBE0 Model. *J. Chem. Phys.* **1999**, 110, 6158–6170.

(20) Weigend, F. Accurate Coulomb-Fitting Basis Sets for H to Rn. *Phys. Chem. Chem. Phys.* **2006**, 8, 1057-1065.

(21) Weigend, F.; Ahlrichs, R. Balanced Basis Sets of Split Valence, Triple Zeta Valence and Quadruple Zeta Valence Quality for H to Rn: Design and Assessment of Accuracy. *Phys. Chem. Chem. Phys.* **2005**, 7, 3297–3305.

(22) Grimme, S.; Antony, J.; Ehrlich, S.; Krieg, H. A Consistent and Accurate Ab Initio Parametrization of Density Functional Dispersion Correction (DFT-D) for the 94 Elements H-Pu. *J. Chem. Phys.* **2010**, 132, 154104.

(23) Grimme, S.; Ehrlich, S.; Goerigk, L. Effect of the Damping Function in Dispersion Corrected Density Functional Theory. *J. Comput. Chem.* **2011**, 32, 1456–1465.

(24) Neese, F.; Wennmohs, F.; Hansen, A.; Becker, U. Efficient, Approximate and Parallel Hartree–Fock and Hybrid DFT Calculations. A ‘Chain-of-Spheres’ Algorithm for the Hartree–Fock Exchange. *Chem. Phys.* **2009**, 356, 98–109.

(25) Neese, F. The SHARK Integral Generation and Digestion System. *J. Comput. Chem.* **2023**, 44, 381–396.

(26) Tao, J.; Perdew, J. P.; Staroverov, V. N.; Scuseria, G. E. Climbing the Density Functional Ladder: Nonempirical Meta–Generalized Gradient Approximation Designed for Molecules and Solids. *Phys. Rev. Lett.* **2003**, 91, 146401.

(27) Staroverov, V. N.; Scuseria, G. E.; Tao, J.; Perdew, J. P. Comparative Assessment of a New Nonempirical Density Functional: Molecules and Hydrogen-Bonded Complexes. *J. Chem. Phys.* **2003**, 119, 12129–12137.

(28) Staroverov, V. N.; Scuseria, G. E.; Tao, J.; Perdew, J. P. Erratum: “Comparative Assessment of a New Nonempirical Density Functional: Molecules and Hydrogen-Bonded Complexes” *J. Chem. Phys.* **2004**, 121, 11507.

(29) Neese, F. Importance of Direct Spin−Spin Coupling and Spin-Flip Excitations for the Zero-Field Splittings of Transition Metal Complexes: A Case Study. *J. Am. Chem. Soc.* **2006**, 128, 10213–10222.

(30) Szabó, P. B.; Csóka, J.; Kállay, M.; Nagy, P. R. Linear-Scaling Open-Shell MP2 Approach: Algorithm, Benchmarks, and Large-Scale Applications. *J. Chem. Theory Comput.* **2021**, 17, 2886–2905.

(31) Lu, T.; Chen, F. Multiwfn: A Multifunctional Wavefunction Analyzer. *J. Comput. Chem.* **2012**, 33, 580–592.

(32) Marenich, A. V.; Cramer, C. J.; Truhlar, D. G. Universal Solvation Model Based on Solute Electron Density and on a Continuum Model of the Solvent Defined by the Bulk Dielectric Constant and Atomic Surface Tensions. *J. Phys. Chem. B* **2009**, *113*, 6378–6396.
